# Quality criteria and certification for paediatric oncology centres: an international cross-sectional survey

**DOI:** 10.1093/intqhc/mzae079

**Published:** 2024-08-09

**Authors:** Sarah P Schladerer, Maria Otth, Katrin Scheinemann

**Affiliations:** Faculty of Health Sciences and Medicine, University of Lucerne, Alpenquai 4, Lucerne 6005, Switzerland; Faculty of Health Sciences and Medicine, University of Lucerne, Alpenquai 4, Lucerne 6005, Switzerland; Division of Hematology-Oncology, Children’s Hospital of Eastern Switzerland, Claudiusstrasse 6, St. Gallen 9006, Switzerland; Department of Oncology, University Children’s Hospital Zurich—Eleonore Foundation, Steinwiesstrasse 75, Zurich 8032, Switzerland; Faculty of Health Sciences and Medicine, University of Lucerne, Alpenquai 4, Lucerne 6005, Switzerland; Division of Hematology-Oncology, Children’s Hospital of Eastern Switzerland, Claudiusstrasse 6, St. Gallen 9006, Switzerland; Department of Pediatrics, McMaster Children’s Hospital and McMaster University, 1200 Main St W, Hamilton, ON L8N 3Z5, Canada

**Keywords:** quality indicators, measurement of quality, certification/accreditation of hospitals, quality improvement, paediatrics, neoplasms

## Abstract

Quality criteria and certification possibilities for paediatric oncology centres vary between countries and are not widely used. An overview of the type and how quality criteria and certifications are used in countries with highly developed healthcare systems is missing. This international cross-sectional survey investigated the use of quality criteria for paediatric oncology centres and whether certification is possible. We sent an online survey to paediatric oncologists from 32 countries worldwide and analysed the survey results and provided regional or national documents on quality criteria and certification possibilities descriptively. Paediatric oncologists from 28 (88%) countries replied. In most countries, the paediatric oncology centres were partly or completely grown historically (75%), followed by the development based on predefined criteria (29%), and due to political reason (25%), with more than one reason in some countries. Quality criteria are available in 20 countries (71%). We newly identified or specified five quality criteria, in addition to those from a previously performed systematic review. Certification of paediatric oncology centres is possible in 13 countries (46%), with a specific certification for paediatric oncology in seven, and a mandatory certification in three of them. The use of quality criteria and certification possibilities are heterogeneous, with quality criteria being more frequently used than certifications. Our study provides an overview of country-specific documents and links with quality criteria, and centre certification possibilities. It can serve as a reference document for stakeholders and may inform an international harmonization of quality criteria and centre certification between countries with similar healthcare systems.

## Introduction

Paediatric cancer is a rare disease, where in 2022 over 250 000 children and adolescents aged 0–19 years were newly diagnosed with cancer globally, and 105 000 died of it [1]. For many of those who survive, cancer and its treatment increase morbidity to various degrees [2, 3]. Since preventive measures and screenings are rarely relevant for paediatric cancer, the primary means of addressing the burden of paediatric cancer overall are high-quality treatment and care. Quality of care can be assessed, and survival rates improved using quality criteria and certification in oncology centres [4–6].

Different factors can influence paediatric cancer treatment and care. Access to healthcare and financial coverage of diagnostic and treatment costs are such factors that differ by country and depend on the healthcare system [7]. In countries with less developed healthcare systems, paediatric cancer care faces different economic and infrastructural barriers, resulting in underdiagnosis and lower survival rates [8–10]. In contrast, in many countries with highly developed healthcare systems, 5-year survival rates over all diagnostic categories now reached ≥85% [11–13]. These survival rates reflect high-quality treatment, supportive care, diagnostics possibilities, and follow-up care.

However, survival rates cannot reflect all aspects of the quality of care provided by paediatric oncology centres. These additional aspects can be measured using quality criteria [14]. Quality criteria vary depending on cultures, environments, available healthcare system resources, and economic situations [15]. A systematic review recently summarized quality criteria for paediatric oncology in countries with highly developed healthcare systems [14]. The authors collected quality criteria from guidelines and publications for paediatric oncology from Canada [6, 16], the USA [17], and Europe [18–24]. In addition to quality criteria, certification of paediatric oncology is essential to measure the adherence to quality criteria, to officially attest standardized and high-quality care of treatment centres, and to make this information transparent for stakeholders. Certification is defined as an external assessment of compliance with given criteria by organizations recognized in the respective field [25]. In Europe, for example, cancer centres of different countries can be certified by the German Cancer Society (Deutsche Krebsgesellschaft, DKG) certification programme or the European Cancer Centre Certification Programme [26]. However, while certification is more common in adult oncology for specific cancer centres or diseases (e.g. breast cancer centre), so far only some paediatric oncology centres in Germany and Switzerland have been certified by the DKG [27].

While some countries with highly developed healthcare systems have published quality criteria for paediatric oncology [14], it is unclear to what extent hospitals have implemented them. Besides, national quality criteria and certification systems might be employed in more countries but are not publicly available. We therefore performed this international cross-sectional survey, covering countries with highly developed healthcare systems, to map quality criteria and certification possibilities used in paediatric oncology.

## Methods

### Eligibility

We selected Organization for Economic Cooperation and Development (OECD) countries with healthcare systems comparable to Switzerland. Their comparability was based on the categorization of 13 health and health system performance core indicators from the Health at Glance report [28]. The considered core indicators belonged to the following four dimensions: (i) ‘health status’ (life expectancy and avoidable mortality), (ii) ‘access to care’ (population and service coverage, and financial protection), (iii) ‘quality of care’ (effective primary secondary and preventive care), and (iv) ‘health system capacity and resources’ (health expenditure, and the number of practising physicians, nurses, and hospital beds) [28]. We selected this set of core indicators as it provides a good picture of the performance of countries’ healthcare systems. It shows how effectively healthcare systems meet the needs of their population in terms of timely delivery of health interventions (prevention, promotion, and rehabilitation), and reflects accessibility, effectiveness, and efficiency of resource utilization. Core indicators were rated in three categories: ‘better than OECD average’, ‘close to OECD average’, and ‘worse than OECD average’ [28]. We included countries if at least five core indicators were in the same or better category than Switzerland (Supplementary Table S1).

### Setting

We collected data between April and September 2023 using SurveyMonkey (https://de.surveymonkey.com/) by sending the survey to one paediatric oncologist (expert) per country, selected via purposive sampling. Whenever possible, we approached the chair of the respective national paediatric oncology society, who can be assumed to know all details regarding characteristics, certification practices, and quality criteria. We asked the experts to forward the survey to a colleague if they could not complete it. We sent reminders after 1 and 6 weeks. We contacted new experts if we did not receive feedback within 10 weeks. If the survey was returned incompletely or the answers were unclear, we recontacted the respondents for clarification.

### Measures

The survey asked about the number of new cancer cases in children and adolescents per year, the number of treatment centres, the availability of 14 specific types of care, and the existence and application of quality criteria and certification possibilities for paediatric oncology centres (Supplementary Table S2). The survey allowed uploading a document stating the country’s quality criteria or certification possibilities for paediatric oncology.

### Data handling

For the availability of the 14 specific types of care, answer options included: available in ‘every centre’, ‘selected centres’, ‘not available (sent abroad)’, and ‘not available (also not abroad)’. If experts from countries with one centre only selected ‘selected centres’, we changed it to ‘every centre’. If website links were provided, we searched the respective documents. If documents were provided, we added the website links, if available. We used Deepl (https://www.deepl.com) and the Microsoft Edge translation function to translate documents not provided in English, German, or French. We extracted the quality criteria from the provided documents (Supplementary Table S3). We excluded documents if they referred to sub-specialties in paediatric oncology or to general aspects of healthcare, hospitals/institutions, paediatrics, or oncology in general [14]. We excluded the information if the origin of quality criteria was local or hospital intern. We extracted and summarized quality criteria from included documents and websites following the procedure and inclusion and exclusion criteria described previously [14]. We extracted the number of children and adolescents aged 0–19 years per country from the World Population Prospects 2022 from the United Nations, Department of Economic and Social Affairs [29].

### Data analysis

We analysed the data descriptively and created charts using Microsoft Excel.

## Results

### Descriptives

We contacted representatives from 32 OECD countries with highly developed healthcare systems (Supplementary Table S1), of which 28 (88%) responded (Table 1). The expert-reported number of children and adolescents newly diagnosed with cancer per year ranges from 14 to 17 000, with 1–200 centres per country treating these patients. The upper age limit for patients in these centres lies between 16 and 21 years. Depending on the underlying diagnosis, some centres treat even older patients. In five countries, the upper age limit differs between centres (Table 1). The three most frequent answers on how treatment centres had developed included: (i) historically grown (75%), (ii) based on predefined criteria for paediatric oncology (29%), and (iii) because of a political reason (25%); 10 countries provided multiple answers (Table 1). The availability of the 14 different types of care varies among countries, and between centres within the same country. In 12 countries, all types of care are available in all or selected centres. Inpatient and outpatient chemotherapy, neurosurgery, orthopaedic surgery, and solid tumour surgery are available in all countries (Supplementary Table S4).

**Table 1.** Incidence and paediatric oncology centre information by included country, listed in alphabetical order.

Country	Population aged 0–19 (thousands)^a^	New paediatric cancer cases/year^b^	Age range case number represents (years)^b^	Number of treatment centres (n)^b^	Age range centres^b^	Development of paediatric oncology centres^b^
Australia	6264	1500	0–18	9	Up to 18 years	Historically grown
Austria	1717	300	0–18	5	Up to 18 years	Historically grown
Belgium	2585	450	0–18	7	Differs between centres/institutions	Historically grown, political reason, predefined criteria for paediatric oncology
Canada	8054	1000	0–17	18	Up to 18 years	Historically grown
Czech Republic	2173	400	0–18	2	Up to 19 years (18 years plus 364 days)	Historically grown, predefined/standardized accreditation process
Denmark	1290	180–200	0–18	4	Up to 18 years	Historically grown, political reason, predefined criteria for paediatric oncology
Estonia	285	50	0–18	2	Up to 18 years	Historically grown
Finland	1155	210	0–18	5	Up to 18 years	Political reason
France	15 120	2500	0–18	30	Differs between centres/institutions	Historically grown, predefined criteria for paediatric oncology, predefined/standardized accreditation process
Germany	15 433	2000	0–16	60	Up to 18 years	Historically grown
Greece	2017	350	0–16	7	Up to 18 years	Predefined criteria for paediatric oncology
Iceland	91	14	0–18	1	Up to 18 years	Historically grown
Ireland	1319	180–200	0–16	1	Up to 16 years	Historically grown
Italy	10 342	NA	0–19	40	Differs between centres/institutions	Historically grown
Latvia	384	50–70	0–18	1	Up to 18 years	Expert did not know
Lithuania	1002	60–70	0–18	2	Up to 18 years	Historically grown, political reason
The Netherlands	3735	600	0–18	1	Up to 21 years, especially in the case of paediatric cancers	Predefined criteria for paediatric oncology, parents and professionals decided that one centre would be the best for the patients
New Zealand	1288	160	0–18	2	Up to 18 years	Initially historically grown, then consolidated into 2 centres for expertise
Norway		200	0–18	4	Up to 18 years	Historically grown, political reason
Poland	7695	1200	0–18	18	Up to 18 years	Historically grown, political reason, geographic location
Portugal	1908	400	0–18	4	Younger than 18 years at diagnosis; remains in paediatrics until treatment ends	Historically grown
Slovakia	1129	180	0–18	3	Up to 18 years	Historically grown
Slovenia	418	70	0–18	1	Depends on diagnosis, ALL up to 22 years, other up to 18 years or even older if disease is more ‘paediatric’	Predefined criteria for paediatric oncology
Spain	9166	1400–1500	0–18	40	Differs between centres/institutions	Historically grown, political reason
Sweden	2448	350	0–18	6	Up to 18 years, some stay longer depending on the treatment and situation (e.g. maturity, diagnosis more adult or paediatric type)	Historically grown
Switzerland	1730	400	0–19	9	Up to 18 years	Predefined criteria for paediatric oncology
UK	15 634	1800	0–14	21	Up to 19 years in some, up to 16 years in others	Historically grown
USA	83 524	17 000	0–20	200	Differs between centres/institutions	Predefined criteria for paediatric oncology, predefined/standardized accreditation process

NA: Not available.

a Estimate 2021 from the UN (from https://population.un.org/wpp/Download/Standard/Population/) [29].

b Data self-reported by participating experts.

### Main results

**Figure 1:**
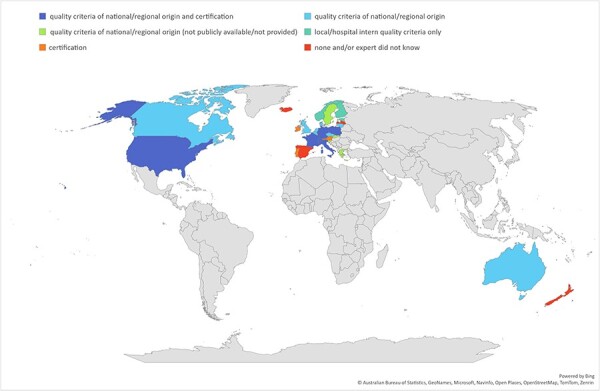
World map showing the availability of quality criteria and certification possibilities among countries, based on expert responses.

Experts from 21 countries (75%) stated that quality criteria for paediatric oncology centres are available, with no criteria in six countries (21%), and one expert being unsure (Supplementary Table S5). However, three documents included local/hospital criteria only, for three countries no document or website with quality criteria was provided or was not publicly available, and one document did not provide quality criteria for paediatric oncology. Fourteen countries remained with quality criteria of national and regional origin [6, 19, 21, 30–44] (Table 2, Fig. 1). Documents of 12 countries stated quality criteria explicitly for paediatric oncology, and three documents focused on oncology centres in general with separate criteria for paediatric oncology centres. Some countries provided more than one document [32, 36, 37, 44]. Quality criteria are implemented in daily practice in 11 of the 14 countries with available documents (Table 2).

**Table 2.** Information of the 14 countries providing information on quality criteria on regional or national level.

Country	Origin of quality criteria	Quality criteria implemented in daily practice?	Name of document or website with quality criteria (translated*) in English	Link to website with quality criteria
Australia	The leading region in the state of Victoria produced/ published statewide standards of care across all tumours, which are adopted nationally	Expert did not know	Victorian paediatric oncology care pathways: Providing optimal care for children and adolescents—Acute leukaemia, central nervous system tumours and solid tumours [30]	https://www.vics.org.au/pics-about-us
Belgium	National local/hospital intern	Never came into effect, currently under revision	Royal Decree establishing standards the specialized care programme for paediatric haemato-oncology and the satellite care programme for paediatric haemato-oncology must meet in order to be recognized [31]*	https://etaamb.openjustice.be/nl/koninklijk-besluit-van-02-april-2014_n2014024119.html
Canada	Provincial: Pediatric Oncology Group of Ontario	No	Measuring the quality of a childhood cancer care delivery system: assessing stakeholder agreement [6]	https://www.valueinhealthjournal.com/article/S1098-3015(13)01701-4/fulltext and https://www.pogo.ca/
Czech Republic	National	Yes	Criteria defining the status of comprehensive cancer centre [32]	https://www.linkos.cz/english-summary/national-cancer-control-programme/czech-cancer-centre-network/criteria-defining-the-status-of-comprehensive-cancer-centre/
Denmark	National	Yes	Danish childhood cancer registry, annual report for the period 01 January 2022 – 31 December 2022 [33]*	https://www.rkkp.dk/kvalitetsdatabaser/databaser/dansk_boernecancer_register/
France	National	Yes	Document paediatric oncology: accreditation criteria for cancer treatment of children and adolescents under 18 years of age [34]*	https://www.e-cancer.fr/Professionnels-de-sante/L-organisation-de-l-offre-de-soins/Traitements-du-cancer-les-etablissements-autorises/Les-autorisations-de-traitement-du-cancer#toc-les-six-mesures-transversales-de-qualit-
			Proposed changes to accreditation criteria for cancer treatment recommendations and facilities [35]*	
			Decree no. 2022–689 of 26 April 2022 on the conditions for setting up cancer treatment facilities [36]*	https://www.legifrance.gouv.fr/jorf/id/JORFTEXT000045668512
			Decree no. 2022–693 of 26 April 2022 on the technical operating conditions for cancer treatment services [37]*	https://www.legifrance.gouv.fr/jorf/id/JORFTEXT000045668609
Germany	National	Yes	Data sheet paediatric oncology: key data sheet, data deficits [38]*	https://www.onkozert.de/organ/kinder/
			Guideline on paediatric oncology, KiOn-RL [21]*	https://www.g-ba.de/richtlinien/47/
Italy	National	Yes	Checklist to apply for a new centre or updating the characteristics of an existing one; Associazione Italiana Ematologia Oncologia Pediatrica [39]*	https://www.aieop.org/web/wp-content/uploads/2023/04/ALLEGATO-3-Autocertificazione-Centri.pdf
Lithuania	National	Yes	Order on the approval of the special requirements for the provision of secondary and tertiary level inpatient personal healthcare services in paediatric onco-haematology [40]*	https://e-seimas.lrs.lt/portal/legalAct/lt/TAD/TAIS.281201/asr
The Netherlands	National	Yes	Responsible and safe care for children with cancer in the the Netherlands –Paediatric Oncology Standards [41]*	No website found
Poland	National	Yes	Standards of the department of paediatric oncology and haematology [42]*	https://onkologia-dziecieca.pl/aktualnosci/news/id/3008-standardy-oddzialu-onkologii-i-hematologii-dzieciecej
Switzerland	National	Yes	Swiss paediatric oncology group statutes [43]*	https://spog.ch/ueber-uns/ueber-spog/
			Quality criteria examined in the application process for highly specialized medicine service mandates	Not publicly available, will be available here: https://www.gdk-cds.ch/de/hochspezialisierte-medizin/bereiche/hochspezialisierte-paediatrische-onkologie
UK	National, National Institute for Health and Care Excellence (NICE)	Yes	Cancer services for children and young people [19]	https://www.nice.org.uk/guidance/qs55
USA	National	Yes	Optimal Resources for Cancer Care—2020 Standards [44]	https://www.facs.org/for-medical-professionals/news-publications/news-and-articles/cancer-programs-news/040722/coc/

A few hundred quality criteria were extracted from the provided documents (Supplementary Table S3). After summarizing and assigning them to the categories defined in the previously conducted systematic review [14], we newly identified or specified five quality criteria (Table 3). We specified the access to fertility clinics and the inclusion of dermatologists, nuclear medicine specialists, spiritual care, and neuropsychological services in the multidisciplinary teams if needed.

**Table 3.** Quality criteria for paediatric oncology centres by thematical categories from Schladerer *et al* [14], with newly identified criteria and specifications in italic.

**Facilities and networks**
Access to the following facilities
Pharmacy; laboratories, including haematology, haematopathology, clinical chemistry, transfusions; microbiology institute; pathology institute; ‘*fertility centre*’ [39]
Paediatric disciplines including: anaesthesia; cardiology; intensive care unit; nephrology; neurosurgery; radiology; radiation therapy; stem cell transplant unit surgery with its sub-specialties
Nuclear medicine; hospital hygiene; adult haematology and oncology
Childhood cancer registries
**Multidisciplinary team (MDT)**
MDT established, including regularly scheduled MDT conferences
Number of paediatric oncology disciplines with multidisciplinary staffing ratios for paediatric oncology
An MDT should consist of representatives from the following disciplines/expertise (disciplines involved depend on the patients’ needs):
Lead MDT Paediatric anaesthesiologist; cardiologist; critical care specialists; ‘*dermatologist*’ [41]; endocrinologist; gastroenterologist; infectious diseases specialists; nephrologist; neurologist; oncologists; oncology nurses; pathologist; ‘*physiotherapis*t’ [31, 42]; pulmonologist; radiologists; surgeons ‘*and additional sub-disciplines if needed*’ [32, 41]
Activity/play therapy staff; complementary and alternative therapies; dentists; dieticians; ear–nose–throat specialist; genetic specialists; laboratory technicians; long-term care (experts); medical secretaries and data managers; ‘*nuclear medicine specialist*’ [41]; occupational therapists; ophthalmologist; pain management experts; palliative care specialists; pharmacists experienced in chemotherapy preparation; psychosocial care/services ‘*incl. spiritual care and neuropsychological service*’ [30]; radiation oncologists; rehabilitation specialists; ward teachers
**Supportive care**
Central Venous Catheter (CVC)
Complication rates: incidence of CVC-associated infection and surgical complication rates (e.g. failure to insert the desired device or leaving the catheter tip in an unacceptable location)
Written policies/procedures for the management of CVC
Existence of supportive care guidelines including supportive care (guidelines) for:
Nausea, vomiting and bowel disturbance; nutritional assessment; fertility (preservation); pain relief, including local protocol for pain relief during procedures and adequate pain management
Dental care; palliative care (including bereavement); psychological or psychosocial care; (neuro-) rehabilitation; social care
School education; cancer education
Febrile neutropenia (F&N)
Guidelines on how to approach a child with F&N (availability, risk-stratified approach, escalation for fever persistence)
Number/proportion of clinical F&N episodes in which the patients with or without microbial focus are treated with first line antibiotics according to local guidelines
Number/proportion of clinical F&N episodes in which patients are admitted to intensive care unit and in which patients die
Number/proportion of fungal health care-associated infections
Time to antibiotic administration
**Treatment**
Number/proportion of patients presented in the interdisciplinary tumour conference (for solid and liquid tumours separately or combined), including its documentation
Protocol compliance (e.g. number of major clinical trial protocol violations)
Number/proportion of clinical trial participation
Number/proportion of refusal and failure to complete treatment
Delay in/wait time to start of: chemotherapy, radiotherapy, first therapeutic intervention, release of pathology results
Medication
Number/proportion of patient safety incidents related to chemotherapy prescriptions, of actual drug or dose errors identified for patients on active treatment, of potential drug or dose errors identified for patients on active treatment, and of elective paediatric oncology ambulatory procedures requiring anaesthesia that are deferred to the next day or beyond due to resource limitation(s)
**Long-term care**
Number/proportion of survivors of childhood cancer with a survivor care plan and of survivors who have their survivorship care plan reviewed 5 years after the end of treatment
Established follow-up and transition structure
**Volume and numbers**
Number of cases per year and provider/clinic

Certification of paediatric oncology centres was indicated to be possible in 13 of the 28 participating countries (46%), not possible in 12 countries (43%), and three experts were unsure (Fig. 1, Supplementary Table S6). After excluding certifications for subspecialties, general certifications for healthcare institutions, or general websites of national paediatric oncology groups, seven countries remained where specific certification for paediatric oncology centres is possible. In three countries, certification is mandatory, and the frequency of recertification varies between 1 and 5 years (Table 4).

**Table 4.** Information about certification specific to paediatric oncology centres by country.

Country	Possibility to certify paediatric oncology centres	Is a certification mandatory?	Name of the organization/programme where one can apply for the certification/accreditation	How often does the recertification/accreditation take place?
France	Yes	Yes	No actual certification but authorization by regional health agencies in agreement with Société Française de Lutte contre les Cancers et les Leucémies de l’Enfant et de l’Adolescent and The French National Cancer Institute (Institut National du Cancer)	Every 4–5 years
Germany	Yes	No	OnkoZert	Yearly
Greece	Yes	No	Ministry of Health	Every 5 years
Italy	Yes	Yes	Associazione Italiana Ematologia Oncologia Pediatrica	Expert did not know
Poland	Yes	Yes	Ministry of Health decides on the need for the new paediatric oncology centre, then the local authorities take care of finding trained staff, location and hospital builds procedures. All centres are associated in the Polish National Paediatric Oncology and Haematology Group.	Depends on the centre, if the hospital as a whole needs accreditation, then the whole process starts. I should say every 3–5 years.
Switzerland	Yes	No	OnkoZert, Highly Specialized Medicine	Yearly
USA	Yes	Expert did not know	Commission on cancer	Every 3 years

## Discussion

### Statement of principal findings

The availability, use, and knowledge about quality criteria and certification possibilities in paediatric oncology vary largely among the 28 surveyed countries with highly developed healthcare systems.

### Interpretation within the context of the wider literature

The experts from Canada, Germany, the UK, and the Netherlands named documents and quality criteria, that were identified in a previously published systematic literature review [14]. Experts from 16 countries provided additional links and documents, which highlights the benefit of questionnaires to gather information on this topic. Quality criteria extracted from these additional documents and websites mainly confirmed previously identified criteria [14], and five criteria were newly identified or specified.

The epidemiological and structural aspects, such as new cancer patients per year and number of centres might impact the perceived needs and awareness for quality criteria and certification. All representatives from the four countries with the largest number of newly diagnosed paediatric cancer patients per year reported having quality criteria and certification possibilities (Supplementary Table S7). One could assume that in countries with smaller patient numbers and fewer centres, the expertise might be higher or perceived higher because the patients are treated at a few places only. Stakeholders of centres with higher patient numbers may assume that they do not need certification due to larger experience and the volume effect [45]. However, Switzerland and Spain are two counterexamples. Although Switzerland is a relatively small country with many centres, three of the nine paediatric oncology centres are certified by the DKG [27]. Spain, with a relatively large number of new cancer patients and many centres, does not have national quality criteria or certification possibilities. Therefore, national regulations and awareness of the ministries of health most probably have a larger impact on the availability and implementation of quality criteria and certifications than the number of newly diagnosed cancer patients, the number of patients per centre, or the local engagement into this topic.

Another explanation for missing quality criteria or certification could be the historical development of paediatric oncology centres—nine countries with historically grown centres had no quality criteria, 17 had no certification possibility. This contrasts with countries whose centres developed based on predefined criteria or through an accreditation process, where these aspects still exist. Centres that have grown historically may be seen as ‘the’ paediatric oncology centres. Establishing quality criteria or certification in these centres could be perceived as questioning the excellence of a centre that has developed over decades. Germany is a counterexample where centres have grown historically, which has not hindered the establishment of centre certification [46].

In most countries with available quality criteria, the criteria are implemented in daily practice. However, this was not the case for Canada, Belgium, and Australia, but the respondents did not comment on the reasons. One reason for not having criteria in daily practice might be an administrative delay between developing and implementing quality criteria. Further, eventually, not all relevant stakeholders have been involved in developing criteria from the beginning, which is crucial to support the implementation by conveying the necessity and feasibility of such criteria. Lastly, tools might be missing to assess the fulfilment of the criteria.

While many countries have quality criteria, most do not have specific certifications for paediatric oncology centres. This could be because insurances, policymakers, or experts do not see a benefit in certifications specific to paediatric oncology beyond having quality criteria. Besides, they might argue that paediatric cancer is a rare disease [47] and certifications specifically designed for rare diseases would also apply to paediatric cancer. However, neither expert named a certification for rare diseases nor indicated that rare disease certifications are not applied to paediatric cancer centres. Lastly, developing quality criteria might be easier than setting up a certification system. Quality criteria can be developed and applied locally, while certification should occur at the national level, requiring higher infrastructural expenditures and independent experts who monitor it. Considering the relatively low incidence of childhood cancer, establishing an independent certification system for paediatric oncology in each country might not be cost-effective. However, costs could be reduced by leveraging existing resources and infrastructure through collaboration with adult oncology certification institutes or countries with similar healthcare structures. Certification by OnkoZert in Germany is an example where paediatric oncology centre certification is integrated into the certification structures of adult cancer centres, and which also conducts certifications in countries with similar healthcare structures, e.g. Switzerland [46]. Studies have shown better treatment outcomes and prognoses for adult patients treated in certified oncology centres [48–50]. Although these are adult studies, similar effects can be expected for the paediatric population. Our results also highlight that the interpretation and definition of certification possibilities for paediatric oncology are inconsistent, as several provided documents did not specifically cover paediatric oncology. This shows the need for education and raising awareness among healthcare professionals.

### Implications for policy, practice, and research

Defining and implementing transparent and internationally harmonized quality criteria and certification for paediatric oncology centres may help to ensure equal access to care for children and adolescents from different countries and influence cross-border care positively. As our data show, not all types of care are provided in every country. To provide this highly specialized care, not only the infrastructure and equipment are needed, but also experienced and trained healthcare professionals. While from a financial, ecological, and medical perspective, it is not reasonable to set up all types of care in every country, access should be enabled via cross-border collaborations. International quality criteria could help to inform decisions on cross-border care and reimbursement, thereby facilitating access to all types of care for all children.

Finally, an international effort to raise awareness on the topic of quality in the care of children and adolescents with cancer is needed. Through the International Society of Paediatric Oncology (SIOP) and its continental branches a large community could be reached. While the definition of a global set of quality criteria that covers all needs may not be feasible due to large differences between continents and regions, establishing a set of minimum quality criteria would be a great first step.

### Strengths and limitations

Though we surveyed experienced experts, self-reports inherently introduce the possibility of reporting bias. Some information might be missing as we provided the answer option ‘don’t know’ instead of a free text field. Strengths include the response rate of 88% and the international perspective by including Europe, North America, and Oceania. By making answers to all relevant questions mandatory and adding the answer option ‘don’t know’, we avoided nonresponse bias.

## Conclusions

In conclusion, the availability, application, and interpretation of quality criteria and certification in paediatric oncology vary widely between countries, with quality criteria being more frequently established than certifications. Our study offers a comprehensive overview by providing country-specific information on paediatric oncology centres, documents and links with quality criteria, and information on centre certification. It can serve as a reference document for different stakeholders and may inform an international harmonization of quality criteria and centre certification between countries with similar healthcare systems, enabling equal access and standardized cross-border care.

## Supplementary Material

mzae079_Supp
