# Supplementary material for: Quality criteria and certification for paediatric oncology centres: an international cross-sectional survey
Source: Int J Qual Health Care. 2024 Aug 9;36(3):mzae079. doi: 10.1093/intqhc/mzae079 (PMC11363956; doi:10.1093/intqhc/mzae079)
Supplement: mzae079_Supp [file mzae079_supp.zip › suppl_data/Supporting Information file 1.pdf]

Supporting Information file 1

Quality criteria and certification for paediatric oncology centres: An international cross-sectional survey

Sarah P. Schladerer[1], Maria Otth[1,2,3*], Katrin Scheinemann[1,2,4*]

[1]Faculty of Health Sciences and Medicine, University of Lucerne, Lucerne, Switzerland

[2]Pediatric Hematology-Oncology Center, Children's Hospital of Eastern Switzerland, St Gallen, Switzerland
[3]Department of Oncology, University Children's Hospital Zurich - Eleonore Foundation, Zurich, Switzerland
[4]Department of Pediatrics, McMaster Children's Hospital and McMaster University, Hamilton, Canada
*shared last authorship

Schladerer SP, Otth M, Scheinemann K. Quality criteria and certification for paediatric oncology centres: An international cross-sectional survey. 2024

Table of contents

Supplemental Table S1: Countries with health care systems comparable to Switzerland based on their scores in 13 core indicators of the Health at a Glance Report1 3

Supplemental Table S2: Questionnaire from the online survey 8

Supplemental Table S3: Quality criteria extracted from provided documents by countries, assigned to the quality criteria from the systematic review, or listed as newly identified quality criteria 10

Supplemental Table S4: Availability of different types of care by country 96

Supplemental Table S5: Detailed information about quality criteria by countries, reasons for exclusion marked in italic 98

Supplemental Table S6: Responses about certifications of paediatric oncology centres, including reasons for exclusion from the final results 102

Supplemental Table S7: Overview of epidemiological data and the availability of quality criteria and certification possibilities from the survey, listed by number of new paediatric cancer patients per year 103

References 105

Supplemental Table S1: Countries with health care systems comparable to Switzerland based on their scores in 13 core indicators of the Health at a Glance Report1

Color Legend	
	Better than/above the OECD average
	Close to OECD average
	Worse than/below the OECD average

Supplemental Table S1: Countries with health care systems comparable to Switzerland based on their scores in 13 core indicators of the Health at a Glance Report1

**Country**	**Dimension**					
	**Health Status**		**Access to care**			
	Life expectancy – years of life at birth	Avoidable mortality: Deaths per 100 000 population (age-standardised)	Population coverage, eligibility – population covered for core set of services (% population)	Population coverage, satisfaction – population satisfied with the availability of quality health care (% population)	Financial protection – expenditure covered by compulsory prepayment schemes (% total expenditure)	Service coverage – population reporting unmet need for medical care (% population)
Switzerland						
Australia						NA
Austria						
Belgium						
Canada						NA
Czech Republic						
Denmark						
Estonia						
Finland						
France						
Germany						
Greece						
Iceland						
Ireland						
Israel						NA
Italy						
Japan						NA
Korea						NA
Latvia				NA		
Lithuania						
Luxembourg						
Netherlands						

New Zealand						NA
Norway						
Poland						
Portugal						
Slovakia						
Slovenia						
Spain						
Sweden						
UK						
US						NA
Chile						NA
Colombia						NA
Costa Rica						NA
Hungary						
Mexico						NA
Turkey						

**Country**	**Dimension**						
	**Health system capacity and resources**				**Quality of care** (4th indicator: Safe primary care: no values for Switzerland)		
	Core Indicator (Selection of 13/24)						
	Health spending – total health spending (per capita, USD using purchasing power parities)	Doctors – number of practising physicians (per 1 000 people)	Nurses – number of practising nurses (per 1 000 people)	Hospital beds – number of hospital beds (per 1 000 people)	Effective primary care - Avoidable COPD admissions (per 100 people, age sex standardised)	Effective preventive care: Mammography screening within the past 2 years (% women aged 50-69)	Effective secondary care: 30 day mortality following AMI (per 100 000 admissions, age sex standardised)
Switzerland							
Australia							
Austria							
Belgium							
Canada							
Czech Republic							
Denmark							
Estonia							
Finland							
France							
Germany							
Greece		*			NA		NA
Iceland							
Ireland							
Israel							
Italy							
Japan					NA		
Korea							
Latvia							
Lithuania							
Luxembourg							
Netherlands							
New Zealand							
Norway							
Poland							
Portugal		*					
Slovakia							
Slovenia							

Spain							
Sweden							
UK							
US							
Chile		**					
Colombia						NA	
Costa Rica		**				NA	NA
Hungary					NA		NA
Mexico							
Turkey							

*Categorised as yellow due to note in the report "include all doctors licensed to practice, resulting in a large over-estimation"[1]
**Categorised as red due to note in the report "include all doctors licensed to practice, resulting in a large over-estimation"[1]

Assessment

Country	Number of core indicators in same or better category as Switzerland (n)	Inclusion (= categorised as similar) if n ≥ 5/13
Australia	7	yes
Austria	8	yes
Belgium	7	yes
Canada	6	yes
Czech Republic	8	yes
Denmark	6	yes
Estonia	6	yes
Finland	8	yes
France	7	yes
Germany	10	yes
Greece	5	yes
Iceland	9	yes
Ireland	6	yes
Israel	8	yes
Italy	9	yes
Japan	5	yes
Korea	6	yes
Latvia	5	yes
Lithuania	6	yes
Luxembourg	9	yes
Netherlands	10	yes
New Zealand	5	yes
Norway	11	yes
Poland	5	yes
Portugal	7	yes
Slovakia	6	yes
Slovenia	7	yes
Spain	10	yes
Sweden	7	yes
UK	6	yes
US	7	yes
Chile	4	no
Colombia	4	no
Costa Rica	2	no
Hungary	3	no
Mexico	1	no
Turkey	4	no

Supplemental Table S2: Questionnaire from the online survey

Question	Sub-questions	Answer options	
**Country and incidence**			
Which country do you represent?		○ Australia<br>○ Austria<br>○ Belgium<br>○ Canada<br>○ Czech Republic<br>○ Denmark<br>○ Estonia<br>○ Finland<br>○ France<br>○ Germany<br>○ Greece<br>○ Iceland<br>○ Ireland<br>○ Israel<br>○ Italy<br>○ Japan	○ Latvia<br>○ Lithuania<br>○ Luxembourg<br>○ Netherlands<br>○ New Zealand<br>○ Norway<br>○ Poland<br>○ Portugal<br>○ Slovakia<br>○ Slovenia<br>○ South Korea<br>○ Spain<br>○ Sweden<br>○ Switzerland<br>○ UK<br>○ US
How many children and adolescents are newly diagnosed with cancer in your country?	Age range the patient number represents (e.g. age 0 – 18 years)	Open-ended response	
	Number of cases per year in your country	Open-ended response	
How many centres/institutions treat childhood and adolescent cancer in your country? (please add a number)		Open-ended response	
**Paediatric oncology centres**			
Up to which age can children and adolescents be treated in paediatric clinics/institutions in your country?		○ Up to 16 years<br>○ Up to 18 years<br>○ Up to 20 years<br>○ Up to 22 years<br>○ Differs between centres/institutions<br>○ Other, please specify	
How have paediatric oncology centres/institutions developed in your country?		○ Historically grown<br>○ Political reason<br>○ Based on predefined criteria for paediatric oncology<br>○ Through a predefined/standardized accreditation process<br>○ Don't know<br>○ Other, please specify	
How are the following types of care distributed in your country?	Inpatient chemotherapy	○ Every centre<br>○ Selected centres<br>○ Not available (sent abroad)<br>○ Not available (also not abroad)	
	Outpatient chemotherapy	○ *Answer options as above*	
	Surgery for solid tumours (not CNS, not orthopaedic)	○ *Answer options as above*	
	Neurosurgery	○ *Answer options as above*	
	Orthopaedic surgery	○ *Answer options as above*	
	Autologous SCT	○ *Answer options as above*	
	Allogeneic SCT	○ *Answer options as above*	
	CAR-T therapy and other cellular therapies	○ *Answer options as above*	
	Radiotherapy (photons)	○ *Answer options as above*	
	Radiotherapy (protons)	○ *Answer options as above*	
	Brachytherapy	○ *Answer options as above*	
	New treatment (phase I/II trials)	○ *Answer options as above*	
	Any clinical trials	○ *Answer options as above*	

	Survivorship clinic	o *Answer options as above*

**Quality criteria**		
Are there quality criteria for paediatric oncology centres/institutions available in your country?		o Yes<br>o No<br>o Don't know
What is the origin of these quality criteria?		o Local/hospital intern<br>o National<br>o Don't know<br>o Other, please specify
Are these quality criteria implemented in daily practice?		o Yes<br>o No<br>o Don't know
Are these quality criteria publicly available?		o No<br>o Yes, please insert link here<br>o Don't know
If there is a document listing these quality criteria, please upload it here:		o Choose file<br>o No file chosen
**Certification**		
Is there a possibility to certify paediatric oncology centres in your country?		o Yes<br>o No<br>o Don't know
Is a certification mandatory?		o Yes<br>o No<br>o Don't know
What is the name of the organization/program where you can apply for the certification/accreditation?		Open-ended response
How often does the re-certification/accreditation take place?		Open-ended response

**Contact**		
If you are interested in the results of our study, you can leave your email address on the next page. We will send the link to the publication to you once the study is published.		o Yes, I want to receive the link to the publication with the results<br>o No, thank you
Please enter your email address:		Open-ended response

Supplemental Table S3: Quality criteria extracted from provided documents by countries, assigned to the quality criteria from the systematic review, or listed as newly identified quality criteria

Country	Document or website	Criterion	Category in document	Category from systematic review	Criterion from systematic review	Reason for exclusion	New criterion
**Denmark**	Danish childhood cancer registry, annual report for the period 01.01.2022 - 31.12.2022[2]	**Indicator 1:** Participation in international studies and international registries:		**Treatment**	Number/Proportion of clinical trial participation		
		**Indicator 2.** 5-year recurrence-free survival: The method of measuring 5-year recurrence-free survival has this year been changed from a raw proportion of patients who neither relapse/progress nor die within 5 years to Kaplan Meier estimated recurrence-free survival. This is to enable more real-time monitoring. The Kaplan Meier method does not require full 5-year follow-up on all patients, which is required for a crude proportion. For the entire population of pediatric cancer patients treated at a pediatric oncology department and diagnosed		Excluded in Review		does not measure quality (in single centres)	
		**Indicator 3.** 5-year survival: Similar to indicator 2, the calculation method for indicator 3 5-year survival has been changed to Kaplan Meier estimated survival. For the entire population of childhood cancer patients treated at a pediatric oncology department and diagnosed on June 1, 2016 and up to and including May 31, 2021		Excluded in Review		does not measure quality (in single centres)	
		As a supplement to indicator 3, supplementary **indicator 3a** is shown, where the patient population is the cancer types treated at all 4 departments.		Excluded in Review			
		**Indicator 5a.** Treatment is initiated within 14 days of the date of investigation start out of all childhood cancer patients		Treatment: Delay in/ Wait time to start of	First therapeutic intervention		
**The Netherlands**	Verantwoorde en veilige zorg voor kinderen met kanker in Nederland - Normering Kinderoncologie[3]	To ensure optimal subspecialty expertise 24 hours a day, 7 days a week, even during holidays/congresses, at least 3 medical specialists must be available for each specialty area of expertise.		Multidisciplinary team (MDT) and other experts	Number of paediatric oncology disciplines with multidisciplinary staffing ratios for paediatric oncology		
		An individual medical specialist must (co-)treat at least 20 patients per year within his own area of expertise.		Long-term care	Number of cases per year and provider/clinic		
		For shared care centers, specific basic requirements established by the SKION apply as well as the volume standard of 20 patients per year.		Volume and Numbers	Number of cases per year and provider/clinic		

10

		**1. Malignancies of the hematopoietic system: 20 patients per subcategory per year**<br>Acute lymphatic leukemia (ALL)<br>• Acute myeloid leukemia (AML)<br>• Chronic myeloid leukemia (CML)<br>• Juvenile myelomonocytic leukemia (JMML)<br>• Myelodysplastic syndrome (MDS)<br>• Histiocytosis (LCH, HLH)<br>• Hodgkin lymphoma<br>• Non-Hodgkin lymphoma	Volume standards for optimal pediatric oncology treatment	Volume and Numbers	Number of cases per year and provider/clinic		
		**2. Central nervous system (CNS) tumors: 20 patients per subcategory per year**<br>• Supra-tentorial tumors (hemisphere)<br>• Supra-tentorial tumors (midline)<br>• Brain tumors in the posterior fossa<br>• Brain tumors in the pons/medulla<br>• Tumors in the myelum/spinal canal<br>• Retinoblastoma	Volume standards for optimal pediatric oncology treatment	Volume and Numbers	Number of cases per year and provider/clinic		
		**3. Solid tumors outside the central nervous system: 20 patients per subcategory per year**<br>• Liver tumors<br>• Germ cell tumors<br>• Neuroblastoma<br>• Kidney tumors<br>• Malignant bone tumors<br>• Rhabdomyosarcoma<br>• Soft tissue sarcomas	Volume standards for optimal pediatric oncology treatment	Volume and Numbers	Number of cases per year and provider/clinic		
		**4. Pediatric intensive care & complications: 20 patients per subcategory per year**<br>Post-operative care after:<br>• Abdominal Surgery<br>• Thoracic Surgery<br>• Central nervous system (CNS) surgery<br>Supportive care:<br>• Infections<br>• Hemo-dynamic<br>• Respiratory<br>• Neurological	Volume standards for optimal pediatric oncology treatment	Volume and Numbers	Number of cases per year and provider/clinic		
		**5. Complex chemo/immunotherapy: 20 patients per subcategory per year**<br>• Complex chemotherapy (multi-day)<br>• Immunotherapy (neuroblastoma)<br>• Autologous stem cell transplantation<br>• Allogeneic stem cell transplantation	Volume standards for optimal pediatric oncology treatment	Volume and Numbers	Number of cases per year and provider/clinic		

		**6. Pediatric surgical treatment outside the CNS: 20 patients per subcategory per year**<br>• General surgery (biopsies, line placement)<br>• Liver Surgery<br>• Kidney Surgery<br>• Other abdominal surgery<br>• Orthopedics<br>• Thoracic Surgery<br>• Retinoblastoma	Volume standards for optimal pediatric oncology treatment	Volume and Numbers	Number of cases per year and provider/clinic		
		**7. Pediatric neurosurgical treatment: 20 patients per subcategory per year**<br>• Supra-tentorial tumors (hemisphere)<br>• Supra-tentorial tumors (midline)<br>• Brain tumors in the posterior fossa<br>• Brain tumors in the pons/medulla<br>• Tumors in the myelum/spinal canal	Volume standards for optimal pediatric oncology treatment	Volume and Numbers	Number of cases per year and provider/clinic		
		**8. Radiotherapy & -pharmacon treatment: 20 patients per subcategory per year**<br>• Conformation radiotherapy<br>• Total body irradiation<br>• Brachytherapy (aka AMORE)<br>• Involved Field technique<br>• 131I-MIBG for neuroblastoma<br>Precision radiotherapy<br>• IMRT<br>• Protons<br>• Rapid arch/tomotherapy<br>• Stereotaxy	Volume standards for optimal pediatric oncology treatment	Volume and Numbers	Number of cases per year and provider/clinic		
		**9. Clinicians: 3 ' dedicated' specialists per relevant (sub)specialty expertise**<br>• Pediatric hemato-oncology oncologist<br>• Pediatric oncologist for solid tumors<br>• Pediatric neuro-oncology oncologist<br>• Pediatric oncologic surgeon<br>• Pediatric neurosurgeon<br>• Pediatric radiotherapist<br>• Pediatric radiologist	Volume standards for optimal pediatric oncology treatment	An MDT should consist of representatives from the following disciplines/expertise (disciplines involved depend on the patients' needs):	Number of paediatric oncology disciplines with multidisciplinary staffing ratios for pediatric oncology<br>Paediatric oncologists<br>Paediatric surgeons<br>Paediatric radiologists		

		**10. Supporting specialties/consultants: 'dedicated' specialists per relevant specialties that guarantee continuous accessibility**<br>• Pediatric endocrinologist<br>• Pediatric cardiologist-cardiologist<br>• Pediatric hematologist<br>• Pediatrician-infectiologist<br>• Pediatric pulmonologist<br>• Pediatrician-Mead, Intestine, Liver (MDL)<br>• Pediatrician-nephrologist<br>• Pediatric neurologist<br>• Nuclear physician<br>• Ophthalmologist<br>• Dermatologist	Volume standards for optimal pediatric oncology treatment	An MDT should consist of representatives from the following disciplines/expertise (disciplines involved depend on the patients' needs):	Number of pediatric oncology disciplines with multidisciplinary staffing ratios for paediatric oncology<br>Paediatric endocrinologist<br>Paediatric cardiologist<br>Paediatric infectious diseases specialists<br>Paediatric pulmonologist<br>Paediatric nephrologist<br>Paediatric neurologist<br>Ophthalmologist		Nuclear medicine specialist and dermatologist specified in the MDT
		**11. Shared care: 20 patients per year**	**Volume standards for optimal pediatric oncology treatment**	Volume and Numbers	Number of cases per year and provider/clinic		
		1.minimum number of patients with a specific tumor to be treated at a pediatric oncology center	Formulate volume standards for pediatric oncology in the Netherlands.	Volume and Numbers	Number of cases per year and provider/clinic		
		2.minimum number of patients to be treated in the pediatric intensive care unit of a pediatric oncology center	Formulate volume standards for pediatric oncology in the Netherlands.	Volume and Numbers	Number of cases per year and provider/clinic		
		3.minimum number of patients with a specific adverse event or complication that should be treated at a pediatric oncology center	Formulate volume standards for pediatric oncology in the Netherlands.	Volume and Numbers	Number of cases per year and provider/clinic		
		4.minimum numbers of specific treatments to be performed in a pediatric oncology center	Formulate volume standards for pediatric oncology in the Netherlands.	Volume and Numbers	Number of cases per year and provider/clinic		
		5.minimum number of patients for whom a specific specialist should be responsible	Formulate volume standards for pediatric oncology in the Netherlands.	Volume and Numbers	Number of cases per year and provider/clinic		
		6.minimum number of patients to be in co-treatment in a shared care center	Formulate volume standards for pediatric oncology in the Netherlands.	Volume and Numbers	Number of cases per year and provider/clinic		

**US**	Optimal Resources for Cancer Care – 2020 Standards[4]	**STANDARD 2.2: Cancer Liaison Physician** PCP's CLP presentations utilize data relevant to the pediatric program with comparison to a national guideline					
		**STANDARD 6.5: Follow-Up of Patients**<br>A 60 percent follow-up rate is maintained for all eligible analytic cases from a rolling 15-year period from the most current year of completed cases or the program's first accreditation date, whichever is shorter. Annual follow-up information is obtained for eligible analytic cases until the patients reach the age of 26 years. Once patients reach the age of 26 years, follow-up attempts are to continue, but the data for the patients are excluded from the follow-up calculations.		Excluded in Review	Exclusion because follow-up for the program only		
		**STANDARD 2.1: Cancer Committee** Cancer committee includes either: Pediatric subspecialists with applicable specialty pediatric certification/licensure (or other documented training) to include:<br>• Pediatric surgical specialist<br>• Pediatric hematology/oncology<br>• Pediatric radiology<br>• Pediatric pathology<br>Pediatric oncology nurse OR create a pediatric subcommittee that reports to the cancer committee to include the disciplines listed above.<br>NOTE: Pediatric radiation oncology is not required under either structure as there is no board certification for this discipline.		An MDT should consist of representatives from the following disciplines/expertise (disciplines involved depend on the patients' needs):	Paediatric surgeons<br>Paediatric oncologists<br>Paediatric radiologists<br>Paediatric pathologist<br>Paediatric oncology nurses		
		**STANDARD 2.2: Cancer Liaison Physician** The CLP is not required to present NCDB data two times per year.					
		**STANDARD 2.3: Cancer Committee Meetings** If a pediatric subcommittee is developed to meet Standard 2.1, it must meet quarterly.		Multidisciplinary team (MDT) and other experts	MDT established, including regularly scheduled MDT conferences		

		**STANDARD 2.4: Cancer Committee Attendance** If a pediatric subcommittee is developed to meet Standard 2.1, the 75% attendance requirement must be met for both the cancer committee and the pediatric subcommittee. The attendance requirement is applied separately to each committee (in other words, the members of the cancer committee must attend 75% of the cancer committee meetings. Members of the subcommittee must attend 75% of the subcommittee meetings).			MDT established, including regularly scheduled MDT conferences		
		**STANDARD 2.5: Multidisciplinary Cancer Conference** For pediatric cancer conferences or for discussion of pediatric cancer patients, the following specialties must be represented:<br>• Pediatric surgical specialist<br>• Pediatric hematology/oncology<br>• Pediatric radiology<br>• Pediatric pathology<br>Radiation oncology with experience treating pediatric patients.		Multidisciplinary team (MDT) and other experts	MDT established, including regularly scheduled MDT conferences<br>Paediatric surgeons<br>Paediatric oncologists<br>Paediatric radiologists<br>Paediatric pathologist<br>Radiation oncologists		
		**STANDARD 3.2: Evaluation and Treatment Services** For pediatric cancer patient care there is a radiologist available to address radiation exposure to the pediatric population.		An MDT should consist of representatives from the following disciplines/expertise (disciplines involved depend on the patients' needs):	Paediatric radiologists		
		**STANDARD 4.1: Physician Credentials** Pediatric cancer patient management must be conducted by a multidisciplinary team including physicians with pediatric training in surgery, hematology/oncology, radiology, and radiation oncology. Physicians are either board certified or demonstrate ongoing pediatric cancer-related education by earning 12 cancer-related CME hours each calendar year.		Multidisciplinary team (MDT) and other experts	MDT established, including regularly scheduled MDT conferences		

		**STANDARD 4.4: Genetic Counseling and Risk Assessment** The process in place pursuant to evidence-based national guidelines for genetic assessment addresses pediatric malignancies such as hypodiploid acute lymphoblastic leukemia, choroid plexus tumor, hepatoblastoma, and anaplastic embryonal rhabdomyosarcoma. In creating the policy and procedure, programs can reference Druker H, Zelley K, McGee RB, Scollon SR, Kohlmann WK, Schneider KA, Wolfe Schneider K. Genetic Counselor Recommendations for Cancer Predisposition Evaluation and Surveillance in the Pediatric Oncology Patient. Clin Cancer Res. 2017 Jul 1;23(13):e91-e97. doi: 10.1158/1078-0432.CCR-17-0834. PMID: 28674117.		An MDT should consist of representatives from the following disciplines/expertise (disciplines involved depend on the patients' needs):	Genetics specialists		
		**STANDARD 4.6: Rehabilitation Care Services** Policies and procedures are in place to guide referral to appropriate pediatric rehabilitation care services on-site or by referral. The availability of pediatric rehabilitation care services is an essential component of comprehensive cancer care, beginning at the time of diagnosis and being continuously available throughout treatment, surveillance, and, when applicable, through end of life. Pediatric rehabilitation care services provide individualized therapies to pediatric and young adult patients appropriate to their developmental level and functional goals.<br>**Rehabilitation professionals** associated with cancer rehabilitation typically include, but are not limited to, pediatric trained:<br>• Physiatrists and advanced practice providers<br>• Physical therapists and physical therapy assistants<br>Occupational therapists and occupational therapy assistants<br>• Speech language pathologists.		Existence of supportive care guidelines including supportive care (guidelines) for	(Neuro-) Rehabilitation<br>Also:<br>Rehabilitation specialists		

		**STANDARD 4.7: Oncology Nutrition Services** Pediatric oncology nutrition services are provided, on-site or by referral, by Registered Dietitian Nutritionists (RDN) with knowledge and skills to address nutrition and hydration requirements and recommendations throughout the continuum of cancer care, including prevention, diagnosis, treatment, survivorship, and palliative care. In particular, nutrition services need to focus on the growing child and recognize all the phases of growth and development from infant to young adult.		Existence of supportive care guidelines including supportive care (guidelines) for	Nutritional assessment		
		**STANDARD 4.8: Survivorship Program** The Survivorship Program Coordinator develops a survivorship program team. Suggested specialties include pediatric physicians, advanced practice providers, nurses, social workers, psychologists, registered dietitians, physical therapists, and other allied health professionals. The survivorship program team determines a list of services and programs, offered on-site or by referral, that address the needs of pediatric cancer survivors.		Long-term care			
		**STANDARD 5.1: College of American Pathologist Synoptic Reporting** PCPs comply with this standard as written for eligible pediatric surgical resection pathology reports. Programs seeking the secondary pediatric accreditation category must complete two internal audits that both comply with the standard as written. One audit for eligible adult surgical resection pathology reports, and a second audit for eligible pediatric surgical resection pathology reports.		Excluded in Review		too specific - criteria for a specific discipline	
		**STANDARD 6.5: Follow-Up of Patients** PCPs and programs seeking the secondary pediatric category comply with this standard as written.		Long-term care	Established follow-up structure		
		**STANDARD 7.2: Monitoring Concordance with Evidence- Based Guidelines** One pediatric-focused study must be completed per standard requirements once each accreditation cycle.					
		**STANDARD 8.1: Addressing Barriers to Care** The barrier chosen is focused on pediatric cancer care. For programs seeking a secondary pediatric category—If a barrier identified by the adult cancer program applies to pediatric cancer care, the same barrier can be used for both adult and pediatric compliance.					

		**STANDARD 8.2: Cancer Prevention Event** Starting in 2026, a prevention event is offered for children and/or young adults that addresses topics such as HPV vaccination, obesity, smoking, sunscreen use, etc. If the prevention event offered by the adult cancer program is applicable to children and/or young adults, it can be used to support compliance for both the adult and pediatric compliance.		Excluded in Review		does not measure quality (in single centres)	
		**STANDARD 8.3: Cancer Screening Event** An event is not required. However, PCPs and programs seeking a secondary pediatric category must conduct screening and active surveillance of syndromic patients (Li-Fraumeni, Beckwith-Wiedemann, DICER1 among others) through an organized program in association with genetics.		Multidisciplinary team (MDT) and other experts	Genetics specialists		
		**STANDARD 9.1: Clinical Research Accrual** The number of accruals to cancer-related clinical research studies meets or exceeds the required percentage of fifty percent. This could include non-Children's Oncology Group (COG) studies. In addition, COG membership is required. The following standards are exempt for standalone Pediatric Cancer Programs (PCP):<br>• Standard 5.3: Sentinel Node Biopsy for Breast Cancer Standard 5.4: Axillary Lymph Node Dissection for Breast Cancer<br>• Standard 5.6: Colon Resection<br>• Standard 5.7: Total Mesorectal Excision<br>• Standard 5.8: Pulmonary Resection<br>• Standard 7.1: Quality Measures		Treatment	Number/Proportion of clinical trial participation		
**Australia**	Victorian pediatric oncology care pathways: Providing optimal care for children and adolescents - Acute leukemia, central nervous system tumors and solid tumours[5]	A family-centred care philosophy is required in the design, promotion, communication and delivery of all aspects of the care pathway for children and adolescents with cancer.	Principles of care: Family-centred care	Excluded in Review		not clear how to measure	
		It is a requirement that all children with a provisional cancer diagnosis be discussed at a paediatric oncology multidisciplinary meeting (MDM), with definitive diagnosis and prospective treatment planning forming the core themes.	Principles of care: Multidisciplinary care	Multidisciplinary team (MDT) and other experts	MDT established, including regularly scheduled MDT conferences		

		Core attendees of the MDM include all experts who are appropriate to the diagnosis.	Principles of care: Multidisciplinary care	Multidisciplinary team (MDT) and other experts	MDT established, including regularly scheduled MDT conferences		
		Documentation and dissemination of meeting outcomes are shared with key stakeholders including the family, the child or adolescent's GP and, if applicable, their paediatrician.	Principles of care: Multidisciplinary care	Treatment	Tumour conference		
		There should be a designated nurse within the MDT allocated to the child or adolescent with cancer with the responsibility to coordinate and communicate care.	Principles of care: Care coordination	An MDT should consist of representatives from the following disciplines/expertise (disciplines involved depend on the patients' needs):	Paediatric oncology nurses		
		The primary oncologist should provide direct clinical consultation at all critical time points during the child or adolescent's treatment. These time points include:<br>• at diagnosis<br>• following investigations measuring response to treatment prior to each new cycle of treatment defined by the protocol<br>• following any significant morbidities<br>• at the end-of-treatment and, if applicable:<br>• at relapse<br>during the transition to treatment with a primarily palliative intent<br>• during the transition to end-of-life care<br>• during bereavement	Principles of care: Consistency of care	Excluded in Review	Communication related	not clear how to measure	
		**Communication** with the child or adolescent with cancer and their family should be:1<br>• individualised<br>• candid and transparent<br>• consistent<br>in plain language (avoiding complex medical terms and jargon)<br>• culturally sensitiveactive, interactive and proactive<br>• ongoing<br>• delivered in an appropriate setting and context<br>offered in a variety of means such as printed and electronic media	Principles of care: Communication	Excluded in Review	Communication related	not clear how to measure	

		For the child or adolescent, information should also be tailored to their age and/or level of cognitive development. Medical play may support the needs of younger children, while opportunities for ‘time alone’ with the healthcare provider may benefit adolescents	Principles of care: Communication			not clear how to measure - too subjective	
		Adult health services managing patients with ‘paediatric□type’ cancers should have links to and advice from a level five or six paediatric cancer service and relevant MDTs	Principles of care: Place of care	Facilities and Networks	Adult hematology and oncology		
		Children and adolescents with ‘adult-type’ cancers should have links to and advice from an adult oncology service and relevant MDTs	Principles of care: Place of care	Facilities and Networks	Adult hematology and oncology		
			Principles of care: Adolescent care	Excluded in Review			
		**The model of care for transition** will also depend on the availability of resources, the risk stratification of the individual and the complexity of care required. This means that some patients will remain in the tertiary adult healthcare sector rather than with their GP. Regardless of risk, a model that incorporates the patient’s GP will reduce the potential for patients to be ‘lost in transition’ and is recommended.<br>Core principles for transitioning to survivorship programs should include the following:20<br>the survivorship healthcare setting should be appropriate to the patient’s age and cognitive development<br>common concerns of young adulthood should be addressed in addition to speciality care. These include fertility, sexual health, contraception, self-management, psychosocial and emotional risk factors and access to healthcare19<br>transition should promote autonomy, personal responsibility, self-reliance and a healthy lifestyle in young adults<br>transition programs should be flexible to meet the changing needs of the young adult<br>the process should be planned with the young adult and their family.	Principles of care: Transition from paediatric to adult care	Long-term care	Established transition structure		
		Eligibility for clinical trial enrolment should be considered for and offered to all children and adolescents diagnosed with cancer	Fundamentals of paediatric oncology practice: Evidence-based practice — research and clinical trials	Treatment	Number/Proportion of clinical trial participation		

		For children who do not meet eligibility criteria, where enrolment is declined, or where a clinical trial is not open, the patient should follow the most recently completed and published 'standard of care' treatment protocol offering the best possible outcome (this may not be the current open trial).		Excluded in Review	Refers to good clinical practice	given by good clinical practice	
		The cancer service should maintain a database of clinical trial enrolment for each diagnosis.		Treatment	Number/Proportion of clinical trial participation		
		Reasons why eligible patients are not enrolled and why patients come off study should be collated and any identified issues examined.		Excluded in Review		too specific	
		**Trials in other disciplines in child and adolescent cancer care**<br>Participation in clinical trials and research should be encouraged in areas other than primary treatment. These include:<br>supportive care – for example, infection control and prevention strategies, palliative care, complications of therapy, nutrition, antiemetic control and fertility21<br>epidemiology – for example, investigation of genetic causes to develop preventative measures22<br>behavioural science – for example, neurocognitive batteries and assessment, identification of at-risk families and children, and psychological and behavioural interventions23<br>nursing – for example, efficacy of patient and family education and reducing illness-related distress.24		Treatment	Participatin in trials		
		**Research and data collection**<br>Other initiatives that should be encouraged include participation in a state-wide approach to trials and participation in national and international cancer registries and survivorship registries.		Facilities and Networks	Childhood cancer registry (Also: Number/Proportion of clinical trial participation)		

		**Supportive care** is an umbrella term used to refer to services that may be required by those affected by cancer. Supportive care meets the needs across the following five domains:<br>physical needs – for example, symptom management, managing and preventing infection, the impact of therapy on growth and development, physiotherapy, occupational therapy<br>psychological needs – for example, the impact on cognition and education, managing stress and anxiety<br>spiritual needs – for example, meaning-making in the context of illness<br>social needs – for example, the child's access to their community, school and social networks<br>information and communication needs of both the child and family.	Fundamentals of paediatric oncology practice: Supportive care	Supportive care	Complication rates: particularly the incidence of CVC associated infection<br>Occupational therapists<br>Psychological or psychosocial care, including provision of/information about social care<br>Provision of school education<br>Provision of cancer education		Spiritual therapy specified in MDT members
		Health services are required to provide access to appropriate information for parents and caregivers to effectively participate in treatment decisions with the healthcare team.	Fundamentals of paediatric oncology practice: Supportive care	Existence of supportive care guidelines including supportive care (guidelines) for	Provision of cancer education		
		Supportive care assessments are shared with the MDT, documented and actioned at critical time points during and after treatment, including:<br>• at diagnosis<br>• following risk assessment<br>• during treatment<br>• at the end-of-treatment<br>• during the transition to survivorship<br>• during the transition to the adult healthcare sector<br>• at relapse<br>during the transition to treatment with a primarily palliative intent<br>• during the transition to end-of-life care<br>• during bereavement.	Fundamentals of paediatric oncology practice: Supportive care	Excluded in Review		given by good clinical practice	

		**Supportive care tools**<br>Recommended tools for supportive care assessment are evidence-based, validated and age-appropriate. Tools may include:<br>a validated psychosocial assessment tool for the patient and family (for example, PAT 2.0™)27<br>a pre-chemotherapy nursing assessment tool (for example, SISOM or the memorial symptom assessment scale)28<br>a performance status tool used prior to each treatment encounter (for example, the Karnofsky or Lanksy score)<br>survivorship guidelines in assessing late complications of therapy (for example, the Children's Oncology Group survivorship guidelines)29<br>• a visual analogue score for chemotherapy-induced nausea and vomiting (for example, the BARF™ scale)<br>• a visual analogue score for pain assessment (for example, the FACES™ pain scale)<br>• validated tools for assessing mucositis in children and adolescents (for example, ChIMES)30		Excluded in Review	Nausea, vomiting and bowel<br>disturbance<br>Pain relief, including local protocol for pain relief procedures and adequate pain management<br>Established follow-up structure<br>Psychological or psychosocial care, including provision of/information about social care	too specific	
		**Clinical practice guidelines**<br>The development and utilisation of CPGs in supportive care is essential to provide optimal care and reduce morbidity and treatment-related mortality.33 Paediatric cancer services should ensure they are following evidence-based supportive care CPGs and should aim to promote national and international collaboration in their development.33	Fundamentals of paediatric oncology practice: Supportive care	Excluded in Review		given by good clinical practice	
		**Neuropsychological demands**<br>Access to neuropsychology services should be risk-adapted and when required, be performed routinely at diagnosis and again at completion of therapy. Neuropsychology assessments should continue to be undertaken in survivorship.	Fundamentals of paediatric oncology practice: Supportive care	Existence of supportive care guidelines including supportive care (guidelines) for	Psychological or psychosocial care, including provision of/information about social care		

		**Psychosocial standards of care**<br>Psychosocial standards for paediatric oncology care are summarised below.35<br>Patients and their families should receive routine psychosocial assessments.<br>Patients in survivorship should receive yearly psychosocial screening.<br>Patients and their families are at high-risk of financial hardship, and targeted referral for supports should be made.<br>Parents and carers are a psychosocially at-risk group and should have early and ongoing assessments.<br>Siblings are an at-risk group and should be provided with appropriate supportive services.<br>Patients and their parents should receive school re-entry and ongoing support to ensure the child remains on track academically.<br>Patients should be provided with opportunities throughout treatment for social interaction.<br>Patients and their families should be provided with psychoeducation, information and anticipatory guidance related to diagnosis, treatment and adaption.<br>Patients should be referred to pain and palliative care services to reduce suffering throughout the disease process.<br>A member of the healthcare team should provide bereavement management support following a child's death.	Fundamentals of paediatric oncology practice: Supportive care	Existence of supportive care guidelines including supportive care (guidelines) for	Psychological or psychosocial care, including provision of/information about social care		
		Every family should be seen by a social worker within one week of diagnosis.	Fundamentals of paediatric oncology practice: Supportive care	Existence of supportive care guidelines including supportive care (guidelines) for	Psychological or psychosocial care, including provision of/information about social care		
		A validated psychosocial screening tool is required to be completed at the time of diagnosis with the results (and ongoing actions) communicated to the MDT and documented in the patient's medical record.	Fundamentals of paediatric oncology practice: Supportive care	Excluded in Review		too specific	
		**Nutritional needs of children with a cancer diagnosis**<br>Paediatric cancer services should give consideration for a nutritional assessment to be undertaken for all new diagnoses to guide the number and type of interventions required and further assessments during treatment.	Fundamentals of paediatric oncology practice: Supportive care	Existence of supportive care guidelines including supportive care (guidelines) for	Nutritional assessment		

		All patients should have a nutritional assessment undertaken at each survivorship consultation.	Fundamentals of paediatric oncology practice: Supportive care	Existence of supportive care guidelines including supportive care (guidelines) for	Nutritional assessment as part of MDT and follow up care		
		**Infection prevention and management:** Patients are required to undergo appropriate infection screening.	Fundamentals of paediatric oncology practice: Supportive care	Excluded in Review		too specific	
		Febrile neutropenia (FN) must be managed according to evidence-based guidelines.	Fundamentals of paediatric oncology practice: Supportive care	Supportive care: Febrile neutropenia (F&N)	Guidelines on how to approach a child with F&N (availability, risk-stratified approach, escalation for fever persistence)		
		Families must receive information and education concerning the prevention and management of infection.	Fundamentals of paediatric oncology practice: Supportive care	Supportive care: Febrile neutropenia (F&N)	Supportive care: education		
		Antimicrobial prophylaxis (viral and fungal) must be prescribed according to trial protocol or institutional guidelines.	Fundamentals of paediatric oncology practice: Supportive care	Excluded in Review		covered in (treatment) protocols	
		Household contacts should be up to date with vaccinations (including live vaccines)	Fundamentals of paediatric oncology practice: Supportive care	Excluded in Review		does not measure quality (in single centres)	
		Annual influenza vaccinations should be provided to the patient and household contacts.	Fundamentals of paediatric oncology practice: Supportive care	Excluded in Review		does not measure quality (in single centres)	
		The paediatric cancer service is required to demonstrate access to an infectious diseases consultant with experience in paediatric oncology	Fundamentals of paediatric oncology practice: Supportive care	An MDT should consist of representatives from the following disciplines/expertise (disciplines involved depend on the patients' needs):	Paediatric infectious diseases specialists		
		In children with FN, antibiotics must be administered within one hour of presentation to hospital, or within 30 minutes for inpatients.	Fundamentals of paediatric oncology practice: Supportive care	Supportive care: Febrile neutropenia (F&N)	Time to antibiotic (TTA) administration		

		All patients should be identified as standard or high-risk of FN and be provided with documentation at diagnosis that identifies their risk category to streamline any required emergency care. This documentation should be updated according to the degree of perceived toxicity during each phase of treatment by a member of the MDT	Fundamentals of paediatric oncology practice: Supportive care	Supportive care: Febrile neutropenia (F&N)	Guidelines on how to approach a child with F&N (availability, risk-stratified approach, escalation for fever persistence)		
		**Palliative care**<br>Palliative care needs should be assessed at all stages of a child's cancer diagnosis. Palliative care can be integrated into the child's management alongside disease-modifying therapy including chemotherapy, radiotherapy, bone marrow transplant and clinical trials. Specialists in palliative care are able to assist the oncology team with advance care planning, symptom management, spiritual care, psychosocial support, linking with community palliative care<br>support services, end-of-life care and bereavement support.	Fundamentals of paediatric oncology practice: Supportive care	Existence of supportive care guidelines including supportive care (guidelines) for	Palliative care (including bereavement)		
		When applicable, palliative care should be provided concurrently with active treatment.	Fundamentals of paediatric oncology practice: Supportive care	Existence of supportive care guidelines including supportive care (guidelines) for	Palliative care (including bereavement)		
		Palliative care should be integrated with care provided by the child's oncologist and other members of the MDT.	Fundamentals of paediatric oncology practice: Supportive care	Supportive care	Palliative care (including bereavement)		
		Referral to palliative care support services should be considered in the context of: - high-risk diagnoses, where three- to five-year event survival is estimated at less than 30 per cent - high-risk disease or multiple relapses - disease progression on treatment - a history of prolonged (more than seven days) or multiple (three or more episodes in a six-month period) intensive care (ICU) admissions - patients without a curative therapeutic approach.	Fundamentals of paediatric oncology practice: Supportive care	Excluded in Review		too specific - criteria for a specific discipline	
		**Fertility**<br>Communicating the options and potential risks to fertility should be discussed at diagnosis, coming off treatment and entry into the survivorship program.	Fundamentals of paediatric oncology practice: Fertility	Existence of supportive care guidelines including supportive care (guidelines) for	Fertility (preservation) discussion		

		An assessment of the risk of infertility is made by the MDT and documented at diagnosis for all patients.<br>Families and, where appropriate, the child or adolescent, should be educated on the potential<br>fertility-related effects of the treatment delivered.<br>Discussions about fertility optimisation and why it may or may not be deemed appropriate should<br>occur as early as clinically possible and prior to treatment commencing.<br>Information should be provided in both verbal and written form regarding potential options, risks and benefits.<br>Families who express an interest in fertility optimisation should be referred and, where clinically feasible,<br>be seen by a fertility service.<br>In those optimisation techniques where efficacy for future fertility cannot be adequately demonstrated,<br>this should be clearly communicated to the child, adolescent and/or family.<br>Families should be aware of the ongoing costs involved in fertility optimisation.<br>All discussions should be documented in the patient's medical record.<br>• Clinical and ethical governance is required in centres offering fertility optimisation.<br>Results regarding semen analyses and tissue biopsies should be communicated to the family as soon<br>as possible, in case the potential for a secondary procedure is possible.<br>Appropriate follow-up during treatment and survivorship is important to discuss results and legalities<br>regarding tissue storage and to monitor reproductive function.	Fundamentals of paediatric oncology practice: Fertility	Existence of supportive care guidelines including supportive care (guidelines) for	Fertility (preservation) discussion		
		**Complementary and alternative medicine in childhood cancer**<br>Complementary and alternative medicine (CAM) refers to a<br>diverse group of practices and products not considered part of evidence-based conventional medicine. CAM is not a<br>substitute for conventional therapy and is not overseen by any health regulating body. In most situations, CAM is integrated into healthcare.	Fundamentals of paediatric oncology practice: Complementary and alternative medicine in childhood cancer	An MDT should consist of representatives from the following disciplines/expertise (disciplines involved depend on the patients' needs):	Complementary and alternative therapies		

		Patients should be encouraged to discuss all CAM with the treating team.	Fundamentals of paediatric oncology practice: Complementary and alternative medicine in childhood cancer	Excluded in Review		not clear how to measure	
		Health services should have a policy governing the use of CAM.	Fundamentals of paediatric oncology practice: Complementary and alternative medicine in childhood cancer	Excluded in Review		too specific - criteria for a specific discipline	
		All discussions of CAM should be shared with the patient's oncologist and/or pharmacy and documented in the patient's medical file.	Fundamentals of paediatric oncology practice: Complementary and alternative medicine in childhood cancer	Excluded in Review		too specific - criteria for a specific discipline	
		**Genetic predisposition to cancer**<br>There should be access to a genetic service with experience in oncology.<br>There should be access to a genetic counsellor in the health service with experience in oncology.<br>All children with cancer should have a complete family history of cancer of at least three generations documented at diagnosis.<br>The emerging family history of cancer should continue to be documented as part of the survivorship program, and consideration of referral to a genetic clinic where new family cancer histories in children or young adults are reported.<br>The health service should have a management strategy that covers the ethical implications of genetic testing in other family members.<br>The genetic clinic should continue to measure the efficacy and yield of findings of referrals to genetic services.	Fundamentals of paediatric oncology practice: Genetic predisposition to cancer	An MDT should consist of representatives from the following disciplines/expertise (disciplines involved depend on the patients' needs):	Genetics specialists		

		**Educating the patient and family** Verbal education to families is paced throughout the initial admission, and time is allowed to process the diagnosis. Education should not be left to the moment of discharge, and families should be aware that education is ongoing and accessible throughout treatment.	Fundamentals of paediatric oncology practice: Educating the patient and family	Existence of supportive care guidelines including supportive care (guidelines) for	Provision of cancer education		
		**Educating the patient and family** Written and/or audio-visual educational information is provided as part of the discharge plan following diagnosis and should also include information targeted to children and adolescents.	Fundamentals of paediatric oncology practice: Educating the patient and family	Existence of supportive care guidelines including supportive care (guidelines) for	Provision of cancer education		
		**Coming off treatment** All patients should attend a formal, multidisciplinary end-of-treatment review. Every patient coming off treatment should be given a full summary of the diagnosis, staging, treatment received and any complications of treatment. Every patient should also receive a tailored surveillance roadmap. The roadmap should identify the recommended timings for clinical tests and investigations as well as referrals to the necessary support services. This should be tailor-made to the individual patient and cover the period from the end-of-treatment to entry into a survivorship program. Copies should be provided to the child/ adolescent and their family, as well as their GP and paediatrician as appropriate.	Fundamentals of paediatric oncology practice: Coming off treatment	Long-term care	Number/Proportion of survivors of childhood cancer with a survivor care plan; ollow-up structure		

		**Survivorship**<br>All children and adolescents who have been treated for cancer or who have undergone an allogeneic stem cell transplant should be referred to a survivorship program.<br>Patients in the survivorship program should follow an approach such as the Children's Oncology Group 2018 Long-term follow-up guidelines to ensure access to appropriate services.<br>The survivorship program should undertake a risk-adapted approach to all patients entering the service for appropriate allocation of resources for those at higher risk of late effects.<br>Paediatric oncology healthcare staff should be available, with access to clinical expertise and resources dependant on the child's risk and current guideline recommendations. This may include representation from areas such as cardiology, endocrinology, fertility, physiotherapy, nutrition, education, psychology, dental, social work, occupational therapy and rehabilitation.<br>All patients should receive tailored educational material in a format appropriate to their level of understanding and language type.<br>The summaries developed at the end-of-treatment must be updated with new information.<br>The surveillance roadmap provided should be updated with new information on entry to the survivorship program, in line with current guidelines and recommendations. This should be made available to the patient and their GP and, if applicable, their paediatrician.	Fundamentals of paediatric oncology practice: Survivorship	Long-term care	Established follow-up structure		

		**Relapse**<br>All patients with relapsed disease are required to be discussed at a paediatric oncology MDM to develop appropriate treatment planning, including decisions about potential clinical trial availability and possible referral to other specialty services including palliative care.<br>The team should present all the information regarding the success rate of conventional relapse treatment plans, regardless of prognosis, and be available to discuss CAM options.<br>The MDT should maintain open and candid communication at all times.<br>Information is sensitively provided to the child/family, in plain language and in a supportive environment.<br>There should be an increased focus on psychosocial support, including exploration of the family's strengths, a focus on enhancing quality of life, ongoing discussion within a multidisciplinary structure and an awareness of maladaptive behaviour, such as emotional or physical withdrawal and refusal to follow through with medical care.<br>Due to the toxicities of many relapse protocols, referral to fertility services should be considered.	Fundamentals of paediatric oncology practice: Relapse			too specific	Focus on relapse
		End-of-life care	Fundamentals of paediatric oncology practice: End-of-life care	An MDT should consist of representatives from the following disciplines/expertise (disciplines involved depend on the patients' needs):	Palliative care specialists		
**Czech Republic**	Criteria defining the status of Comprehensive Cancer Centre[6]	In a Comprehensive Cancer Centre, at least 5 doctors with specialised qualification in the field of medical oncology, each having a full-time job in that Centre.	1) Personnel criteria<br>a) The specialty of medical oncology has the following staff at its disposal:	An MDT should consist of representatives from the following disciplines/expertise (disciplines involved depend on the patients' needs):	Paediatric oncologists		

		In a Children's Cancer Centre (for childhood solid tumours), at least 5 doctors with specialised qualification in the fields of paediatric medicine and medical oncology at the same time, or doctors with specialised qualification in the field of paediatric oncology and haematology, each having a full-time job in that Centre.	1) Personnel criteria a) The specialty of medical oncology has the following staff at its disposal:	An MDT should consist of representatives from the following disciplines/expertise (disciplines involved depend on the patients' needs):	Paediatric oncologists Number of paediatric oncology disciplines with multidisciplinary staffing ratios for paediatric oncology		
		Non-medical health care professionals with qualification according to Act No. 96/2004 Coll., on conditions of obtaining and recognition of qualification for the performance of non-medical occupations in health service and for the due performance of activities related to the provision of health care and amending certain related laws, and to the Decree No. 424/2004 Coll. of the Czech Ministry of Health; in a Children's Cancer Centre, paediatric nurses and a paediatric psychologist, among others.	1) Personnel criteria a) The specialty of medical oncology has the following staff at its disposal:	An MDT should consist of representatives from the following disciplines/expertise (disciplines involved depend on the patients' needs):	Paediatric oncology nurses Psychosocial care/services		
		Doctors with specialised qualification in the field of radiation oncology, at least one doctor per 150 new patients per year; at least 3 doctors with this qualification, each having a full-time job in that Centre.	1) Personnel criteria b) The specialty of radiation oncology has the following staff at its disposal:	An MDT should consist of representatives from the following disciplines/expertise (disciplines involved depend on the patients' needs):	Radiation oncologists Number of paediatric oncology disciplines with multidisciplinary staffing ratios for paediatric oncology		
		At least 2 medical radiological physicists with specialised qualification in the field of radiotherapy, each having a full-time job in that Centre. The required number of radiological physicists also depends on the number of radiotherapy units and on the number of patients treated per year, according to the recommendation of the European Federation of Organisations for Medical Physics from 1997 (hereinafter referred to as the " EFOMP 7/1997").	1) Personnel criteria b) The specialty of radiation oncology has the following staff at its disposal:	An MDT should consist of representatives from the following disciplines/expertise (disciplines involved depend on the patients' needs):	Paediatric radiologists Number of paediatric oncology disciplines with multidisciplinary staffing ratios for paediatric oncology		
		Medical engineers with specialised qualification, radiological physicists, radiological technicians, biomedical engineers, and biomedical technicians in numbers as recommended by EFOMP 7/1997.	1) Personnel criteria b) The specialty of radiation oncology has the following staff at its disposal:			too specific - criteria for a specific discipline	

		Radiological technologists:<br>linear accelerator – 3 technologists per 1 shift per 1 instrument, each having a full-time job,<br>cobalt unit - 2 technologists per 1 shift per 1 instrument, each having a full-time job,<br>- simulator – 2 technologists, each having a full-time job,<br>treatment planning system - 2 technologists, each having a full-time job, if planning is not ensured by other qualified professionals (such as radiological technicians, radiological physicists).	1) Personnel criteria<br>b) The specialty of radiation oncology has the following staff at its disposal:			too specific - criteria for a specific discipline	
		Other staff: doctors undergoing specialist training in radiation oncology, radiological physicists undergoing specialist training in radiotherapy, general nurses, social workers, other health care professionals, and auxiliary and other medical staff. The actual number of these workers depends on the equipment and scope of activity of the respective Centre.	1) Personnel criteria<br>b) The specialty of radiation oncology has the following staff at its disposal:			too specific - criteria for a specific discipline	
		Professional staff for brachytherapy:<br>Doctors with specialised qualification in the field of radiation oncology, their number depending on their scope of activity and the number of patients, but at least 2 doctors, each having a full-time job.<br>A least one full-time medical radiological physicist with specialised qualification in the field of radiotherapy, the number depending on the number of instruments and number of patients treated per year, according to the EFOMP 7/1997<br>- Radiological technologists:<br>simulator or C-arm – at least one technologist,<br>remote-controlled afterloading instrument – at least two technologists.<br>Other staff: medical engineers with specialised qualification, biomedical engineers, biomedical technicians, perisurgical nurses and other workers in numbers as recommended by EFOMP 7/1997.	1) Personnel criteria<br>b) The specialty of radiation oncology has the following staff at its disposal:			too specific - criteria for a specific discipline	

		a) Radiotherapy equipment:<br>two or more linear accelerators (from which at least two dual accelerators).<br>brachytherapy instrument (automatic afterloading system, C-arm, application room),<br>simulator or CT-simulator,<br>3D treatment planning system,<br>equipment for absolute, relative and in vivo dosimetry,<br>modelling laboratory,<br>equipment for specialised radiotherapeutic techiques (IMRT,TBI).	2) Technical criteria			too specific - criteria for a specific discipline	
		b) Equipment for systemic treatment:<br>comprehensive diagnostic possibilities for systemic treatment - establishment of all recommended prediction parameters,<br>central preparation unit for cytostatic drugs,<br>fully-equipped short-stay ward with the possibility of two-shift operation and long-term outpatient treatment<br>close cooperation with ICU (intensive care unit).	2) Technical criteria			too specific - criteria for a specific discipline	
		CCCs and ChCCs have been established on the basis of health care facilities dealing specifically with cancer patients. The core of a Cancer Centre consists of a cancer care facility which is comprehensively equipped to provide radiotherapy and systemic treatment (including drugs which can be only administered in the Cancer Centres), both to hospitalised patients and to outpatients treated at short-stay wards.	3) Other criteria	Facilities and Networks	Radiation therapy		
		interdisciplinary cooperation with adequate surgical, internal and complementary disciplines	3) Other criteria<br>a) CCCs and ChCCs ensure:	Facilities and Networks	Paediatric surgery		
		interdisciplinary cooperation with adequate specialists in paediatrics with respect to surgical and internal disciplines, anaesthesiology and resuscitation, anatomical pathology, medical genetics, radiology and imaging techniques, and with doctors having specialised qualification in the field of paediatric radiology (this item applies to ChCCs only).	3) Other criteria<br>a) CCCs and ChCCs ensure:	Facilities and Networks	Paediatric surgery		
		coordination of professional help to cancer care facilities at lower level in a respective region, and maintenance of professional cooperation with them,	3) Other criteria<br>a) CCCs and ChCCs ensure:	Excluded in Review		does not measure quality (in single centres)	

		education and research in oncology,	3) Other criteria a) CCCs and ChCCs ensure:	Excluded in Review		does not measure quality (in single centres)	
		specialist training in medical oncology, radiation oncology, paediatric oncology and haematology, based on the obtained accreditation.	3) Other criteria a) CCCs and ChCCs ensure:	Excluded in Review		certification of professionals	
		The housed imaging methods in the fields of radiology, sonography, nuclear medicine and endoscopy comply with the contemporary standards,	3) Other criteria b) Requirements on diagnostic methods:	Excluded in Review		too specific - criteria for a specific discipline	
		the laboratory methods of cancer diagnosis are available in the fields of biochemistry, haematology and histopathology, including the perioperative histological diagnosis,	3) Other criteria b) Requirements on diagnostic methods:	Facilities and Networks	Laboratories: hematology, hematopathology, clinical chemistry, transfusion		
		oncogenetic consultations are available in indicated cases.	3) Other criteria b) Requirements on diagnostic methods:	Multidisciplinary team (MDT) and other experts	Genetics specialists		
		An independent inpatient department for cancer patients is available, with 30 beds at least, capable of providing intensive care to these patients	3) Other criteria c) Requirements on medical oncology:	Facilities and Networks	Paediatric Intensive care unit		
		a daily-operated outpatient department for cancer patients is available,	3) Other criteria c) Requirements on medical oncology:	Excluded in Review	Excluded because standard of care		
		an existing unified system for centralised preparation of cytostatic drugs,	3) Other criteria c) Requirements on medical oncology:	An MDT should consist of representatives from the following disciplines/expertise (disciplines involved depend on the patients' needs):	Pharmacists experienced in chemotherapy preparation		
		a short-stay ward to provide chemotherapy to outpatients is available.	3) Other criteria c) Requirements on medical oncology:	Excluded in Review	Excluded because standard of care		
		All needed instrumental equipment is available, particularly instruments for irradiation, dosimetry and planning, as required by the Czech Society for Radiation Oncology, Biology and Physics,	3) Other criteria d) Requirements on radiation oncology:	Facilities and Networks	Radiation therapy	too specific - criteria for a specific discipline	
		an inpatient department is available, dedicated partly or fully to the care of cancer patients undergoing irradiation procedures (this can be shared with the department of medical oncology)	3) Other criteria d) Requirements on radiation oncology:	Facilities and Networks	Radiation therapy		

		a daily-operated outpatient department of radiation oncology is available	3) Other criteria d) Requirements on radiation oncology:	Facilities and Networks	Radiation therapy		
		Ability to provide specialised care with respect to surgical diagnosis and treatment of solid malignant tumours in the following fields of oncology: digestive, thoracic, mammary, dermal, gynaecological, urological, orthopaedic, neurosurgical, otorhinolaryngological, and maxillofacial,	3) Other criteria e) Surgical care is specifically focused on cancer patients, ensuring:	Facilities and Networks	Paediatric surgery		
		assurance of close cooperation among the surgeons and physicians with specialised qualification in the field of anatomical pathology, particularly histopathologists, and the provision of guaranteeing their responsibility for the quality of tissue samples sent for examination.	3) Other criteria e) Surgical care is specifically focused on cancer patients, ensuring:	Excluded in Review	Excluded because standard of care	not clear how to measure	
		Assured availability of specialized outpatient care and second opinions, particularly in the fields of pain management, nutrition, clinical psychology, stoma treatment,	3) Other criteria f) Supportive and palliative care:	An MDT should consist of representatives from the following disciplines/expertise (disciplines involved depend on the patients' needs):	Gastroenterologists Dieticians Psychosocial care/services		
		availability of treatment of infection-related complications,	3) Other criteria f) Supportive and palliative care:		Written policies/ procedures for the management of CVC		
		existence of a well-developed system of palliative care of cancer patients for whom anticancer treatment was terminated, in compliance with the principles of continuity of cancer care, as well as the physician's commitment not to abandon the patient,	3) Other criteria f) Supportive and palliative care:	Existence of supportive care guidelines including supportive care (guidelines) for	Palliative care (including bereavement)		
		the assurance that specialized inpatient palliative care is available.	3) Other criteria f) Supportive and palliative care:	Existence of supportive care guidelines including supportive care (guidelines) for	Palliative care (including bereavement)		
		comprehensive documentation on cancer patients, including the findings of imaging methods (both through conventional and digital media), as well as archived histopathological samples	3) Other criteria g) The Centre guarantees the maintenance of documentation on cancer patients, with particular focus on:	Excluded in Review		given by good clinical practice	

		records on patients' informed consents to treatment,	3) Other criteria g) The Centre guarantees the maintenance of documentation on cancer patients, with particular focus on:	Excluded in Review		given by good clinical practice	
		due documentation on the individual stages of cancer treatment, i.e. operation protocols, records on radiation therapy and chemotherapy, all in compliance with the respective standards	3) Other criteria g) The Centre guarantees the maintenance of documentation on cancer patients, with particular focus on:	Excluded in Review		given by good clinical practice	
		the submitting of a comprehensive Report on Malignant Neoplasm for each cancer patient for whom the responsibility was duly assumed, and the submitting of regular Follow-up Reports,	3) Other criteria g) The Centre guarantees the maintenance of documentation on cancer patients, with particular focus on:	Excluded in Review		given by good clinical practice	
		records on the numbers of cancer patients sorted by individual diagnoses, the readiness to make these numbers public in de-identified form, the submitting of data into clinical registries monitoring the quality of cancer care,	3) Other criteria g) The Centre guarantees the maintenance of documentation on cancer patients, with particular focus on:	Facilities and Networks	Childhood cancer registry		
		evaluation of results of rendered cancer diagnosis and treatment using self-evaluation and local programmes of health care quality control.	3) Other criteria g) The Centre guarantees the maintenance of documentation on cancer patients, with particular focus on:	Excluded in Review		given by good clinical practice	
		follow-up care provided to each cancer patient with respect to the type of his/her disease, the overall condition and preferences; this follow-up care is provided by the CC or, if otherwise agreed, by another specialist.	3) Other criteria h) The Centre guarantees the follow-up care of cancer patients, with particular focus on:	Long-term care	Established follow-up structure		

		all necessary documentation on the patient’s treatment and the possibility of further cooperation between the CC and the follow-up physician.	3) Other criteria h) The Centre guarantees the follow-up care of cancer patients, with particular focus on:	Long-term care	Established follow-up structure		
		in each stage of the patient’s examination, treatment or follow up, the patient knows (and it is obvious from our documentation) who is responsible for his/her treatment and to whom he/she can refer in case of problems or if consultation is needed	3) Other criteria i) The Centre has established responsibility for cancer patients, namely	Excluded in Review		not clear how to measure	
		compliance with the guidelines by expert societies as regards cancer diagnosis and treatment	3) Other criteria j) The Centre guarantees clinical trials and implementation of new procedures:	Excluded in Review		given by good clinical practice	
		a transparent system for the participation of patients in clinical trials and the implementation of new procedures, which involves specifying responsibility, as well as cooperation with the ethics committee.	3) Other criteria j) The Centre guarantees clinical trials and implementation of new procedures:	Treatment	(Number/Proportion of) clinical trial participation		
		the establishment and smooth running of a regional network of cancer care facilities,	3) Other criteria k) The Centre actively participates in the organization of cancer care in the respective region and guarantees (in addition to the highest level of cancer care):	Excluded in Review		does not measure quality (in single centres)	
		consultations to cancer care facilities at lower levels of hierarchy.	3) Other criteria k) The Centre actively participates in the organization of cancer care in the respective region and guarantees (in addition to the highest level of cancer care):	Excluded in Review		does not measure quality (in single centres)	

**Switzerland**	Swiss peadiatric oncology group statutes[7]	The members are obliged to support the objectives of the association.<br>In particular, they are required to a) participate in national and international therapy optimization studies, projects and registries;	Obligations of member institutions	Treatment	Number/Proportion of clinical trial participation		
**Italy**	Check list to apply for a new center or updating the characteristics of an existing one; Associazione Italiana Ematologia Oncologia Pediatrica[8]<br><br>**Legend:**<br>I = Valid for Centers dealing with Immunologic Diseases<br>PDTA = Diagnostic-Therapeutic and Care Pathways<br>FUP = Follow-up	HEADQUARTERS<br>specify	Headquarters				
		NUMBER OF NEW DIAGNOSES PER YEAR<br>Solid tumors<br>Onco-hematologic cancers Non-oncologic hematology<br>Immunologic diseases<br>Number of diagnoses of Leukemias and/or Lymphomas ... Number of diagnoses of Solid Tumors<br>...<br>Number of diagnoses by area of interest (specify)	Number of new diagnoses per year	Volume and Numbers	Number of cases per year and provider/clinic		
		NUMBER OF PATIENTS TAKEN IN BY YEAR<br>Solid tumors<br>Onco-hematologic cancers Non□oncologic hematology<br>Immunologic diseases	Number of patients taken in by year	Volume and Numbers	Number of cases per year and provider/clinic		
		Ordinary hospitalization (daytime and continuous)	Services needed (on-site or off-site)	Excluded in Review		given by good clinical practice	
		Laminar flow or Low Microbial Load (I) beds.	Services needed (on-site or off-site)	Excluded in Review		does not measure quality (in single centres)	
		Outpatient clinic of (patients in treatment and out of treatment)	Services needed (on-site or off-site)	Excluded in Review	Excluded: standard of care		

		CNT-accredited hematopoietic transplantation/cell therapy/gene therapy unit/ GITMO/JACIE	Services needed (on-site or off-site)	Excluded in Review		too specific - criteria for a specific discipline	
		Hematopoietic stem cell transplantation outpatient clinic	Services needed (on-site or off-site)	Facilities and Networks	Stem cell transplant unit		
		Processing unit with processing, preservation, storage and Distribution of hematopoietic stem cells for clinical use, cell product characterization and biological qualification activities	Services needed (on-site or off-site)	Facilities and Networks	Stem cell transplant unit		
		Psycho-oncology service	Services needed (on-site or off-site)	An MDT should consist of representatives from the following disciplines/expertise (disciplines involved depend on the patients' needs):	Psychological or psychosocial care, including provision of/information about social care		
		Pediatric palliative care services	Services needed (on-site or off-site)	Existence of supportive care guidelines including supportive care (guidelines) for	Palliative care (including bereavement)		
		Fertility preservation center	Services needed (on-site or off-site)		Fertility (preservation) discussion		Fertility preservation centre
		Family Genetic Counseling Service and Prenatal Diagnosis (I)	Services needed (on-site or off-site)	An MDT should consist of representatives from the following disciplines/expertise (disciplines involved depend on the patients' needs):	Genetics specialists		
		Laboratory Analysis	Services needed (on-site or off-site)	Facilities and Networks	Laboratories: hematology, hematopathology, clinical chemistry, transfusion		
		Microbiology Laboratory (Bacteriology, Virology, Mycology, Parasitology)	Services needed (on-site or off-site)	Facilities and Networks	Microbiology Institute		

		Laboratory of Cytofluorimetry, Cytogenetics and Molecular Biology	Services needed (on-site or off-site)	Facilities and Networks	Laboratories: hematology, hematopathology, clinical chemistry, transfusion		
		Pathologic anatomy	Services needed (on-site or off-site)	Facilities and Networks	Pathology		
		Immunohematology and transfusion medicine h24	Services needed (on-site or off-site)	Facilities and Networks	Laboratories: hematology, hematopathology, clinical chemistry, transfusion		
		Centralized pharmacy with presence of antineoplastic drug unit (AHPU)	Services needed (on-site or off-site)	Facilities and Networks	Pharmacy		
		Radiology (CT, MRI) and Radiology with expertise in imaging Chest MRI for Pediatric immunology	Services needed (on-site or off-site)	Facilities and Networks	Paediatric radiology		
		Nuclear medicine with diagnostic imaging (PET and scintigraphy)	Services needed (on-site or off-site)	Facilities and Networks	Nuclear medicine		
		Inpatient ward for metabolic radiotherapy	Services needed (on-site or off-site)		Radiation therapy		
		Videofluoroscopy	Services needed (on-site or off-site)	Excluded in Review		too specific - criteria for a specific discipline	
		Digestive Endoscopy	Services needed (on-site or off-site)	Excluded in Review		too specific - criteria for a specific discipline	
		Sedo-analgesia service for invasive maneuvers	Services needed (on-site or off-site)		Paediatric anaesthetics		
		Pediatric intensive care unit	Services needed (on-site or off-site)	Facilities and Networks	Paediatric intensive care unit		

		Cardiologist, infectivologist, pulmonologist, endocrinologist, neurologist, neuropsychiatrist, nephrologist, gastroenterologist, ophthalmologist, physiatrist, physical therapist, nutritionist, dietitian, dermatologist, medical geneticist, otolaryngologist, odontostomatologist, audiometrist	Services needed (on-site or off-site)	An MDT should consist of representatives from the following disciplines/expertise (disciplines involved depend on the patients' needs):	Paediatric cardiologist<br>Paediatric infectious diseases specialists<br>Paediatric pulmonologist<br>Paediatric endocrinologist<br>Paediatric neurologist<br>Paediatric nephrologist<br>Psychosocial care/services<br>Paediatric nephrologist<br>Paediatric gastroenterologist<br>Ophthalmologist<br>Dieticians<br>Genetics specialists<br>Ear–nose–throat specialist		
		Social worker; teachers; professors; volunteers	Services needed (on-site or off-site)	An MDT should consist of representatives from the following disciplines/expertise (disciplines involved depend on the patients' needs):	Ward teachers<br>Psychosocial care/services		
		Pediatricians with documented clinical experience of at least 2 years and with established experience in hematology/oncology/immunology in both outpatient and inpatient settings	Skills and professional development	An MDT should consist of representatives from the following disciplines/expertise (disciplines involved depend on the patients' needs):	Paediatric oncologists		
		Hematologists with documented clinical experience of at least 2 years in the field of immunodeficiencies (I)	Skills and professional development	An MDT should consist of representatives from the following disciplines/expertise (disciplines involved depend on the patients' needs):	Paediatric oncologists		

		Oncologists with documented clinical experience of at least 2 years in the field Pediatric and with established experience in both outpatient and inpatient settings	Skills and professional development	An MDT should consist of representatives from the following disciplines/expertise (disciplines involved depend on the patients' needs):	Paediatric oncologists		
		Medical personnel with documented clinical experience of at least 2 years in the field Pediatric and with established both outpatient and inpatient	Skills and professional development	An MDT should consist of representatives from the following disciplines/expertise (disciplines involved depend on the patients' needs):	Paediatric oncologists		
		Nurses with expertise in oncology of at least 2 years and with established Both outpatient and inpatient experience	Skills and professional development	An MDT should consist of representatives from the following disciplines/expertise (disciplines involved depend on the patients' needs):	Paediatric oncology nurses		
		Nurses with documented clinical expertise of at least 2 years and with established Experience in the management of the immunocompromised patient (I)	Skills and professional development	An MDT should consist of representatives from the following disciplines/expertise (disciplines involved depend on the patients' needs):	Paediatric oncology nurses		
		Surgeons with expertise in minimally invasive techniques, for treatment of solid tumors, with expertise in pediatric oncology and clinical experience and surgery of at least 2 years	Skills and professional development	An MDT should consist of representatives from the following disciplines/expertise (disciplines involved depend on the patients' needs):	Paediatric surgeons		

		Neurosurgeons, for treatment of brain and spinal tumors with expertise in Pediatric neuro-oncology field and clinical and surgical experience of at least 2 years	Skills and professional development	An MDT should consist of representatives from the following disciplines/expertise (disciplines involved depend on the patients' needs):	Neurosurgery		
		Orthopedists, for treatment of sarcomas of bone, with expertise in pediatric orthopedic oncology and clinical and surgical experience of at least 2 years	Skills and professional Development	Supportive care: Central Venous Catheter (CVC)	Orthopaedics		
		Ophthalmologists, for retinoblastoma treatment, with expertise in oncology Pediatric ocular and clinical and surgical experience of at least 2 years	Skills and professional development	An MDT should consist of representatives from the following disciplines/expertise (disciplines involved depend on the patients' needs):	Ophthalmologist		
		Radiotherapists with expertise in pediatric oncology and experience 2-year clinic	Skills and professional development	An MDT should consist of representatives from the following disciplines/expertise (disciplines involved depend on the patients' needs):	Radiation oncologists		
		Health care support staff with proven pediatric experience With a focus on hematology/oncology/immunology	Skills and professional development	An MDT should consist of representatives from the following disciplines/expertise (disciplines involved depend on the patients' needs):			
		Use of pediatric PDTAs and application of protocols and guidelines national and international	Skills and professional Development	Excluded in Review		covered in (treatment) protocols	
		Drafting PDTA transition period from pediatric to adult center	Skills and Professional development				
		Continuing professional development in the specific field of leukemia, lymphoma, and solid tumors, with demonstrated participation in working groups in National scope (AIEOP) and international scope (SIOP, EBMT, COG)	Skills and professional development	Excluded in Review		certification of professionals	

		Staff health care in updating professional continuous through Demonstrated attendance at annual IPINET and ESID meetings every other year (I)	Skills and professional development	Excluded in Review		certification of professionals	
		Adverse event management and safety of care	Application of security measures recommended by The ministry	Excluded in Review		given by good clinical practice	
		Intra- and postoperative complications management	Application of security measures recommended by The ministry	Excluded in Review		given by good clinical practice	
		Operating room technological equipment (appropriateness of surgical instrument use. pediatric, microscope, ultrasonic aspirator, optical and magnetic navigator,	Application of security measures recommended by The ministry	Excluded in Review		too specific - criteria for a specific discipline	
		Intraoperative neurophysiology and expertise in neuroendoscopy)	Application of security measures recommended by The ministry	Excluded in Review		too specific - criteria for a specific discipline	
		Radiotherapy technology equipment and quality control (linear accelerator capable of performing treatments with intensity-modulated techniques, CT simulator, image registration and target volume contouring, treatment planning system for conformal techniques and intensity-modulated techniques using "inverse planning" algorithms, digital systems for the online verification of radiant treatments)	Application of security measures recommended by the ministry	Excluded in Review		too specific - criteria for a specific discipline	
		Proper use of drugs: Single Therapeutic Record, Corporate Procedure for the storage, prescription, preparation distribution and administration of drugs	Application of security measures recommended by The ministry	Excluded in Review		given by good clinical practice	
		Proper use of high-risk or high-attention medications	Application of security measures recommended by The ministry	Excluded in Review		given by good clinical practice	
		CSS Guidelines "Traceability, Collection, Transportation, Storage, and Archiving of Cells, Tissues for Pathological Anatomy Investigations."	Application of security measures recommended by The ministry	Excluded in Review		too specific - criteria for a specific discipline	

		AIFA Accreditation for Phase I Studies.	Application of security measures recommended by The ministry	Excluded in Review		too specific - country specific	
		Data Manager	Application of security measures recommended by the ministry	An MDT should consist of representatives from the following disciplines/expertise (disciplines involved depend on the patients' needs):	Medical secretaries and data managers		
		Study Coordinator	Application of security measures recommended by the ministry	An MDT should consist of representatives from the following disciplines/expertise (disciplines involved depend on the patients' needs):	Medical secretaries and data managers		
**Lithuania**	Order on the approval of the special requirements for the provision of secondary and tertiary level inpatient personal healthcare services in pediatric onco-haematology[9]	3. The following shall be ensured in the secondary level paediatric oncohaematology inpatient services:	Requirements for aspis				
		3.1. the diagnosis and treatment of non-malignant diseases of the blood and immune system, other than rare diseases, in accordance with the diagnostic and treatment protocols and/or diagnostic and treatment methodologies for the relevant disease, and with the testing and treatment methods set out in Annex 1 to the Regulations;<br>3.2. patients with suspected cancer (diagnosis codes according to the International Statistical Classification of Diseases and Related Health Problems, tenth revised and expanded edition "Systemic List of Diseases" (Australian Modification, ICD-10-AM) (hereafter referred to as "ICD-10-AM") C00-C96), a rare disease of the blood or the immune system, for referral to an ASF providing tertiary level paediatric oncohaematology inpatient services for diagnosis and treatment;	Requirements for aspis	Excluded in Review		covered in (treatment) protocols	

		3.3. ology personal healthcare services in accordance with the Description of Requirements for the Provision of Radiology Personal Healthcare Services, approved by the Order of the Minister of Health of the Republic of Lithuania of 24 July 2015 No.V-881 "On the Approval of the Description of Requirements for the Provision of Radiology Personal Healthcare Services" (hereinafter - the Description of Requirements for the Provision of Radiology Personal Healthcare Services);	Requirements for aspis	Excluded in Review		too specific - country specific	
		3.4. Referral of patients aged 18 years or over who are receiving inpatient treatment for an oncological or haematological disease to adult physicians, according to a procedure approved by the head of the NICU, which includes joint meetings between paediatricians and adult physicians, feedback between treating physicians, and recommendations for patient care;	Requirements for aspis	Multidisciplinary team (MDT) and other experts	Adult hematology and oncology		
		3.5. monitoring the health status of patients with oncohaematological diseases in accordance with Order No V-288 of the Minister of Health of the Republic of Lithuania of 28 February 2014 "On the approval of the procedure for long-term monitoring of the health status of patients with chronic diseases".	Requirements for aspis	Excluded in Review		given by good clinical practice	
		4. The following shall be ensured in tertiary level paediatric oncohaematology inpatient services:	Requirements For aspis				
		4.1. diagnosis and treatment of oncological, rare blood and immune system diseases, in accordance with the protocols for diagnosis and treatment of the relevant disease and/or diagnostic and treatment methodologies and using the methods of testing and treatment specified in Annex 2 to the Description, except in cases where the patient requires a method of treatment that is not available in the ASFU, in which case the institution must organise the patient's transfer and ensure his/her transportation to another ASFU where this method of treatment is available;	Requirements for aspis	Excluded in Review		covered in (treatment) protocols	
		4.2. Cultures, genetic tests (can be performed under contract with another healthcare institution);	Requirements for aspis	An MDT should consist of representatives from the following disciplines/expertise (disciplines involved depend on the patients' needs):	Genetics specialists		
		4.3. 24-hour access to medicines for oncological and haematological diseases;	Requirements For aspis	Facilities and Networks	Pharmacy		

		4.4. transfusions of its components irradiated with ionising radiation (to inactivate leukocytes);	Requirements for aspis	Excluded in Review		too specific - criteria for a specific discipline	
		4.5. the provision of personal healthcare services in radiology in accordance with the Requirements for the Provision of Personal Healthcare Services in Radiology;	Requirements for aspis	Facilities and Networks	Paediatric radiology		
		4.6. oncology radiotherapy services (which may be performed under contract with another healthcare institution) in accordance with the description of the procedure of special requirements for the provision of oncology radiotherapy inpatient personal health care services, approved by the Order of the Minister of Health of the Republic of Lithuania of 30 April 2004 No.V-310 "On the Approval of the Description of the Procedure of the Procedure of the Special Requirements for the Provision of Oncology Radiotherapy Inpatient Personal Health Care Services," as well as under general anaesthesia;	Requirements for aspis	Facilities and Networks	Radiation therapy		
		4.7. the option to start inpatient medical rehabilitation services or to be referred to another healthcare facility providing inpatient medical rehabilitation services;	Requirements for aspis	Existence of supportive care guidelines including supportive care (guidelines) for	(Neuro-) Rehabilitation		
		4.8. Referral of patients aged 18 years or over who are receiving inpatient treatment for an oncological or haematological disease to adult physicians, according to a procedure approved by the head of the NICU, which includes joint meetings between paediatricians and adult physicians, feedback between treating physicians, and recommendations for patient care;	Requirements for aspis	Long-term care	Established transition structure		
		4.9. monitoring the health status of patients with oncohaematological diseases in accordance with Order No V-288 of the Minister of Health of the Republic of Lithuania of 28 February 2014 "On the approval of the procedure for long-term monitoring of the health status of patients with chronic diseases".	Requirements for aspis	Long-term care	Established follow-up structure		
		5. The following team of specialists provides inpatient paediatric oncohaematology services at the tertiary level:	Requirements for personal health care Professionals				

		5.1. paediatric oncohaematologist;	Requirements for personal health care professionals	An MDT should consist of representatives from the following disciplines/expertise (disciplines involved depend on the patients' needs):	Paediatric oncologists		
		5.2. disease doctor;	Requirements for personal health care professionals	An MDT should consist of representatives from the following disciplines/expertise (disciplines involved depend on the patients' needs):	Paediatric oncologists		
		5.3. a nurse in general practice;	Requirements for personal health care professionals	An MDT should consist of representatives from the following disciplines/expertise (disciplines involved depend on the patients' needs):	Paediatric oncology nurses		
		5.4. cine psychologist;	Requirements for personal health care Professionals		Psychosocial care/services		
		5.5. a full-time employee.	Requirements for personal health care Professionals	Excluded in Review		too unspecific/broad	
		6. The following team of specialists provides ethical paediatric oncohaematology inpatient services:	Requirements for personal health care Professionals				
		6.1. pediatric oncohaematologist;	Requirements for personal health care professionals	An MDT should consist of representatives from the following disciplines/expertise (disciplines involved depend on the patients' needs):	Paediatric oncologists		

		6.2. a nurse in general practice;	Requirements for personal health care professionals	An MDT should consist of representatives from the following disciplines/expertise (disciplines involved depend on the patients' needs):	Paediatric oncology nurses		
		6.3. cine psychologist;	Requirements for personal health care professionals	An MDT should consist of representatives from the following disciplines/expertise (disciplines involved depend on the patients' needs):	Psychosocial care/services		
		6.4. a full-time employee.	Requirements for personal health care Professionals	Excluded in Review		too unspecific/broad	
		7. In a secondary-level paediatric oncohaematology inpatient service:	Requirements for personal health care Professionals				
		7.1. at least one paediatric oncohaematologist;	Requirements for personal health care professionals	An MDT should consist of representatives from the following disciplines/expertise (disciplines involved depend on the patients' needs):	Paediatric oncologists		
		7.2. During their stay in the NICU (at night, weekends and holidays), patients must be supervised by a paediatric oncohaematologist or a paediatric physician and a general nurse, and the services of a paediatric oncohaematologist shall be available at any time of the day, in accordance with procedures laid down by the NICU manager;	Requirements for personal health care professionals	An MDT should consist of representatives from the following disciplines/expertise (disciplines involved depend on the patients' needs):	Paediatric oncologists Paediatric oncology nurses		

		7.3. When needed, patients can consult with tertiary-level specialists in paediatric oncohaematology inpatient services for their examination and treatment.	Requirements for personal health care professionals	An MDT should consist of representatives from the following disciplines/expertise (disciplines involved depend on the patients' needs):	Paediatric oncologists		
		8. In a tertiary-level paediatric oncohaematology inpatient service:	Requirements for personal health care Professionals				
		8.1. two or more paediatric oncohaematologists with at least 2 years' experience in paediatric oncohaematology services;	Requirements for personal health care professionals	An MDT should consist of representatives from the following disciplines/expertise (disciplines involved depend on the patients' needs):	Paediatric oncologists		
		8.2. 24-hour uninterrupted services of a paediatric oncohaematologist, in accordance with the procedures laid down by the head of the institution;	Requirements for personal health care professionals	An MDT should consist of representatives from the following disciplines/expertise (disciplines involved depend on the patients' needs):	Paediatric oncologists		
		8.3. at least two-thirds of nurses providing paediatric onco-haematology inpatient services must have at least 2 years' experience in paediatric onco-haematology services;	Requirements for personal health care professionals	An MDT should consist of representatives from the following disciplines/expertise (disciplines involved depend on the patients' needs):	Paediatric oncology nurses		
		8.4. Consultation of doctors providing secondary-level paediatric onco-haematology inpatient services on the investigation and treatment of patients is ensured as needed.	Requirements for personal health care professionals	An MDT should consist of representatives from the following disciplines/expertise (disciplines involved depend on the patients' needs):	Paediatric oncologists		

		9. A general nurse involved in secondary and/or tertiary inpatient paediatric onco□haematology services must complete a minimum of 36 hours of training on 'Characteristics of patient care with chemotherapy' within 6 months of commencing work in this field.	Requirements for personal health care professionals	Excluded in Review		certification of professionals	
		10. In the event of a diagnosis or confirmation of oncological disease, the patient's investigation and treatment plan is discussed by a multidisciplinary team of physicians (the "DGS team") consisting of<br>a paediatric oncohaematologist,<br>a paediatric surgeon with experience in operating on children with oncological diseases,<br>a radiotherapy oncologist,<br>a pathologist,<br>a radiologist<br>and/or specialists with other specialised professional qualifications as required.<br>The DGS discusses, as appropriate, the medical condition of patients with rare blood or immune diseases or oncological or haematological diseases in the event of a change in their medical condition (e.g. relapse or progression of disease).	Requirements for personal health care professionals	Multidisciplinary team (MDT) and other experts	MDT established, including regularly scheduled MDT conferences<br>Paediatric oncologists<br>Paediatric surgeons<br>Radiation oncologists<br>Paediatric pathologist<br>Paediatric radiologists		
		11. the patient receives advice from an appropriately qualified healthcare professional(s) when needed.	Requirements for personal health care Professionals	Excluded in Review		not clear how to measure - too subjective	
		12. The facility, which provides inpatient secondary level paediatric oncohaematology<br>services, must include:<br>12.1. a hospital providing inpatient paediatric oncohaematology services:<br>4<br>12.1.1. patient care facilities, which must include:<br>12.1.1.1. with a call button for medical staff (for each patient bed);<br>12.1.1.2. (stationary, foldable, etc.) for the person caring for the patient;<br>12.1.1.3. and/or infusion equipment, programmable auto-injectors (per patient bed	Requirements for facilities and medical devices	Excluded in Review		too specific	

		12. The facility, which provides inpatient secondary level paediatric oncohaematology services, must include: 12.1. a hospital providing inpatient paediatric oncohaematology services: 12.1.2. s providing invasive and/or interventional procedures and allowing the use of different types of anaesthesia when necessary;	Requirements for facilities and medical devices	Facilities and Networks	Paediatric anaesthesiology		
		12. The facility, which provides inpatient secondary level paediatric oncohaematology services, must include: 12.1. a hospital providing inpatient paediatric oncohaematology services: 12.1.3. s that ensure confidential communication between children and/or parents and/or professionals;	Requirements for facilities and medical devices	Excluded in Review	Excluded because standard of care		
		12. The facility, which provides inpatient secondary level paediatric oncohaematology services, must include: 12.1. a hospital providing inpatient paediatric oncohaematology services: 12.1.4. for playing and learning;	Requirements for facilities and medical devices	An MDT should consist of representatives from the following disciplines/expertise (disciplines involved depend on the patients' needs):	Activity/play therapy staff		
		12. The facility, which provides inpatient secondary level paediatric oncohaematology services, must include: 12.1. a hospital providing inpatient paediatric oncohaematology services: 12.1.5. in the atalla - an environment that is age-appropriate for the child;	Requirements for facilities and medical devices	Excluded in Review		too unspecific/broad	
		12. The facility, which provides inpatient secondary level paediatric oncohaematology services, must include: 12.1. a hospital providing inpatient paediatric oncohaematology services: 12.1.6. single wards, mainly double wards; single wards must make up at least 10 % of the total number of wards in the unit, but there must be at least 1 single ward;	Requirements for facilities and medical devices	Excluded in Review		too specific	
		12. The facility, which provides inpatient secondary level paediatric oncohaematology services, must include: 12.2. other facilities necessary for the application of the diagnostic technologies and treatment methods referred to in Annex 1 to the Regulations;	Requirements for facilities and medical devices	Excluded in Review		too unspecific/broad	

		12. The facility, which provides inpatient secondary level paediatric oncohaematology services, must include:<br>12.3. a pediatric intensive care unit (Paediatric intensive care II)	Requirements for facilities and medical devices	Facilities and Networks	Paediatric Intensive care unit		
		13. The facility providing tertiary level paediatric oncohaematology inpatient services must include:<br>13.1. a hospital providing inpatient paediatric oncohaematology services:<br>13.1.1. facilities for the care of the animals, which shall include:<br>13.1.1.1. with a call button for medical staff (for each patient bed);<br>13.1.1.2. ionari, foldable, etc.) for the person caring for the patient;<br>13.1.1.3. an oxygen feeder that delivers oxygen (100% liquefied petroleum gas) and other oxygen equipment (oxygen humidifier, oxygen catheters, etc);<br>13.1.1.4. and/or infusion equipment, programmable auto-injectors (per patient bed);<br>13.1.1.5. i or mobile equipment for monitoring vital signs (blood pressure, heart rate, saturation);	Requirements for facilities and medical devices	Excluded in Review		too specific	
		13. The facility providing tertiary level paediatric oncohaematology inpatient services must include:<br>13.1.2. s providing invasive and/or interventional procedures and allowing for different types of anaesthesia to be administered when needed;	Requirements for facilities and medical devices	Facilities and Networks	Paediatric anaesthetics		
		13. The facility providing tertiary level paediatric oncohaematology inpatient services must include:<br>13.1.3. s that ensure confidential communication between children and/or parents and/or professionals;	Requirements for facilities and medical devices	Excluded in Review	Excluded because standard of care		
		13. The facility providing tertiary level paediatric oncohaematology inpatient services must include:<br>13.1.4. for playing and learning;	Requirements for facilities and medical devices	An MDT should consist of representatives from the following disciplines/expertise (disciplines involved depend on the patients' needs):	Activity/play therapy staff		

		13. The facility providing tertiary level paediatric oncohaematology inpatient services must include:<br>13.1.5. Indoors, an environment that is age-appropriate for the child;	Requirements for facilities and medical devices	Excluded in Review		does not measure quality (in single centres)	
		13. The facility providing tertiary level paediatric oncohaematology inpatient services must include:<br>13.1.6. single wards, mainly double wards, with single wards accounting for at least 10% of the total number of wards in the unit, but at least 1 single ward;	Requirements for facilities and medical devices		Paediatric Intensive care unit		Facility for playing and learning
		13. The facility providing tertiary level paediatric oncohaematology inpatient services must include:<br>13.2. other facilities necessary for the application of the diagnostic technologies and treatment methods referred to in Annex 2 to the Regulations;	Requirements for facilities and medical devices	Excluded in Review		too unspecific/broad	
		13. The facility providing tertiary level paediatric oncohaematology inpatient services must include:<br>13.3. a pediatric intensive care unit (pediatric intensive care III).	Requirements for facilities and medical devices	Facilities and Networks	Paediatric Intensive care unit		
		14. one of the premises referred to in point 13 and one of the premises referred to in point 14 of the Schedule shall contain:<br>14.1. a first aid kit of a health care facility, which complies with the description of the first<br>aid kit of a personal health care facility, approved by Order No V-450 of 11 July 2003 of the<br>Minister of Health of the Republic of Lithuania "On the description of the first aid kit of a personal<br>health care facility The requirements set out in the 'First Aid Kit and the approval of the Competence of Personal Health<br>and Pharmaceutical Professionals in First Aid';	Requirements for facilities and medical devices	Excluded in Review		given by good clinical practice	
		14. one of the premises referred to in point 13 and one of the premises referred to in point 14 of the Schedule shall contain:<br>14.2. a cabinet (if cytotoxic drugs are prepared in the treatment room);	Requirements for facilities and medical devices	Excluded in Review		too specific - criteria for a specific discipline	
		14. one of the premises referred to in point 13 and one of the premises referred to in point 14 of the Schedule shall contain:<br>14.3. a tuvas with long-term temperature monitoring, a platelet storage incubator-mixer	Requirements for facilities and medical devices	Excluded in Review		too specific - criteria for a specific discipline	

		15. secondary and/or tertiary inpatient paediatric onco-haematology services are prescribed by a paediatric onco-haematologist. A referral for secondary and/or tertiary inpatient paediatric onco-haematology services shall be made by a paediatric onco-haematologist, a general practitioner or a physician with another professional qualification. First and emergency medical care shall be provided in accordance with the procedure and scope of emergency medical care as approved by Order No V-208 of 8 April 2004 of the Minister of Health of the Republic of Lithuania "On the approval of the procedure and scope of emergency medical care".	Procedure for the provision of inpatient paediatric oncohaematology services	Excluded in Review		too specific - country specific	
		16. After the oncological diagnosis, the patient is counselled and an individualised examination and treatment plan is drawn up by the DGS team, in which the patient and/or his/her legal representative are invited to participate. The patient aged 16 years and over, or the patient's legal representative under 16 years of age, shall have the right to be informed of the conclusions of the discussion of the DGS team. Subsequent DGS team discussions shall be organised as necessary to continue or adjust the examination and/or treatment of the patient.	Procedure for the provision of inpatient paediatric oncohaematology services	Excluded in Review		given by good clinical practice	
		17. secondary and/or tertiary inpatient paediatric onco-haematology services: 17.1. conditions for the permanent presence of legal representatives with the patient, and for the patient's communication with other family members;	Procedure for the provision of inpatient paediatric oncohaematology services	Excluded in Review		not clear how to measure	
		17. secondary and/or tertiary inpatient paediatric onco-haematology services:<br>17.2. The child and his or her relatives (legal representatives, brothers or sisters) receive psychological support from a medical psychologist or psychotherapist, as required;	Procedure for the provision of inpatient paediatric oncohaematology services	Existence of supportive care guidelines including supportive care (guidelines) for	Psychological or psychosocial care, including provision of/information about social care		
		17. secondary and/or tertiary inpatient paediatric onco-haematology services:<br>17.3. medical rehabilitation services in accordance with the indications and procedures set out in the Order of the Minister of Health of the Republic of Lithuania No V-50 of 17 January 2008 "On the Organisation of Medical Rehabilitation and Sanatorium (Anti-Recidivism) Treatment";	Procedure for the provision of inpatient paediatric oncohaematology services	Existence of supportive care guidelines including supportive care (guidelines) for	(Neuro-) Rehabilitation		

		17. secondary and/or tertiary inpatient paediatric onco-haematology services:<br>17.4. Social support for the patient and his/her relatives (legal representatives, siblings) (starting after the patient is diagnosed with onco-haematological disease, informing and assisting with applications for social support, encouraging participation in quality-of-life activities, etc).	Procedure for the provision of inpatient paediatric oncohaematology services	Existence of supportive care guidelines including supportive care (guidelines) for	Psychological or psychosocial care, including provision of/information about social care		
		18. The paediatric oncohaematologist treating the patient has the right to consult a clinical pharmacologist depending on the patient's medical condition.	Procedure for the provision of inpatient paediatric oncohaematology services	Excluded in Review		given by good clinical practice	
		19. In the case of inpatient services at secondary and/or tertiary level in paediatric onco□haematology, the form No 003/a "History of inpatient treatment" referred to in the Order of the Minister of Health of the Republic of Lithuania of 29 November 1999 No. The form No 066/a-LK 'Statistical card of a person undergoing in-patient treatment', approved by Order No 687 of the Minister of Health of the Republic of Lithuania of 26 November 1998 'On Approval of Forms of Medical Accounting Documents'. Electronic documents shall be processed in accordance with the description of the procedure for the use of the Information System for Electronic Health Services and Cooperation Infrastructure, approved by Order No V-657 of the Minister of Health of the Republic of Lithuania of 26 May 2015 "On the approval of the description of the procedure for the use of the Information System for Electronic Health Services and Cooperation Infrastructure"	Procedure for the provision of inpatient paediatric oncohaematology services	Excluded in Review		too specific - country specific	

**Belgium**	Royal Decree establishing standards the specialized care program for pediatric hemato-oncology and the satellite care program for pediatric hemato-oncology must meet in order to be recognized[10]	Art. 4.§ 1. In order to obtain recognition for a specialised care programme for paediatric haemato-oncology, an existing need must be fully justified. § 2. For the purposes of recognition, this existing need shall be demonstrated at least by the treatment of a minimum of 50 new patients per year under the age of 16 with haemato-oncological diseases or serious non-oncological haematological diseases, which may require a stem cell transplant, either during the year before the application for recognition or as an average during the last three years before the application for recognition.<br><br>In order to remain recognised, the specialised care programme for paediatric haemato-oncology shall demonstrate every three years that it reaches the level of activity referred to in the first paragraph in the last year or as an average during the last three years before the renewal of approval.<br><br>For the purposes of this paragraph, "new patient" means the patient whose treatment, after the initial diagnosis, is mainly carried out in the specialized care program. § 3. In order to be and remain recognised, the specialised paediatric haemato-oncology care programme, if it has a stem cell transplantation unit intended exclusively for the treatment of children, shall carry out a minimum of 10 allogeneic stem cell transplants in children either the year preceding recognition or renewal of approval or as an average during the last three years before the application for recognition or renewal of approval.	2. - Specialized Care Program for Pediatric Hemato-Oncology - Section 2. - Minimum activity level	Volume and Numbers	Number of cases per year and provider/clinic		

		Art. 5.Proof of the minimum level of activity referred to in Article 4, §§ 2 and 3, shall be provided by a copy of the reports of the multidisciplinary oncology consultation referred to in Article 37, for each patient treated within the framework of the care programme.<br><br>For the purposes of determining the level of activity referred to in Article 4, the said reports shall show that the treatment has mainly taken place in the specialised care programme.<br><br>The same report of multidisciplinary oncological consultations can demonstrate both the activity level referred to in Article 4, § 2 and the activity level referred to in Article 4, § 3.<br><br>A copy of these reports shall be kept at the disposal of the approved authority in the hospital for a period of ten years and shall subsequently be destroyed.	2. - Specialized Care Program for Pediatric Hemato-Oncology - Section 2. - Minimum activity level	Excluded in Review		does not measure quality (in single centres)	
		Art. 6.The necessary measures shall be taken at the hospital to ensure the security of the data referred to in Article 5.<br><br>For the purposes of the previous paragraph, and without prejudice to the application of the Law of 8 December 1992 on the protection of privacy with regard to the processing of personal data and its implementing decrees, at least the following measures shall be taken by the controller referred to in the same law:<br>1° the drawing up of a nominative list of the persons to whom access to such health data is authorised and the signing by these persons of a commitment of confidentiality;<br>2° determine the modalities of the written procedures which determine the protection of such health data and which limit their processing to processing in accordance with the intended purpose;<br>3° the development of organizational and technical measures that ensure that only authorized persons are given access to these personal data.	2. - Specialized Care Program for Pediatric Hemato-Oncology - Section 2. - Minimum activity level	Excluded in Review		does not measure quality (in single centres)	
		Art. 7.The specialized care program for pediatric hemato-oncology shall have at least:<br>1° a hospitalization unit with a minimum of 18 beds to be able to accommodate the patients of the care program at all times;<br>2° a unit for day hospitalization;<br>3° an outpatient consultation room;<br>4° an intensive care unit and resuscitation.	2. - Specialized Care Program for Pediatric Hemato-Oncology - Section 3. - Required infrastructure and environmental elements	Facilities and Networks			

		Art. 8.The specialized care program for pediatric hemato-oncology has, within the hospital of which it is part, :<br>1° a room for nurses where they can organize their specific work;<br>2° a diagnostic and therapeutic examination room insofar as this is not already present elsewhere in the hospital;<br>3° a game and educational space;<br>4° a kitchen;<br>5° a sitting area where the parents or supervisors can retreat insofar as this is not already present elsewhere in the hospital;<br>6° separate sanitary facilities for patients, staff and visitors, and a shower room for admitted children and their companion who stays overnight.	2. - Specialized Care Program for Pediatric Hemato-Oncology - Section 3. - Required infrastructure and environmental elements	Excluded in Review	Excluded because standard of care		
		Art. 9.The different types of spaces for children and parents or accompanying persons must be wheelchair accessible.	2. - Specialized Care Program for Pediatric Hemato-Oncology - Section 3. - Required infrastructure and environmental elements	Excluded in Review	Excluded because standard of care		
		Art. 10.For children admitted to traditional hospitalization, the stay in patient rooms is organized in such a way that the children are grouped according to their age as much as possible.	2. - Specialized Care Program for Pediatric Hemato-Oncology - Section 3. - Required infrastructure and environmental elements	Excluded in Review		too unspecific/broad	
		Art. 11.There must be sufficient rooms equipped with a baby bath and a care pillow, in order to ensure the hygienic care of infants. The parents or supervisors must have the opportunity to help care for their child in the room where they are being cared for.	2. - Specialized Care Program for Pediatric Hemato-Oncology - Section 3. - Required infrastructure and environmental elements	Excluded in Review		too unspecific/broad	
		Art. 12.Each room must be able to accommodate a parent or accompanying person, both during the day and at night.	2. - Specialized Care Program for Pediatric Hemato-Oncology - Section 3. - Required infrastructure and environmental elements	Excluded in Review		too unspecific/broad	

		Art. 13.The play and educational space is at least in use during normal working hours. It must be equipped with furniture, toys and other facilities adapted to the target group concerned. This space must have an area of at least 25 m2.	2. - Specialized Care Program for Pediatric Hemato-Oncology - Section 3. - Required infrastructure and environmental elements	Excluded in Review		too specific - country specific	
		Art. 14.Furniture, floor and toys must be disinfectable and washable and must be cleaned using a fixed procedure.	2. - Specialized Care Program for Pediatric Hemato-Oncology - Section 3. - Required infrastructure and environmental elements	Excluded in Review		does not measure quality (in single centres)	
		Art. 15.The stay must be safe for all persons, and in particular for children.<br>The necessary measures are taken to prevent patients from leaving the ward without this being justified.<br><br>In all areas accessible to children, parents or supervisors must be able to be present with their children and attention must be paid to the prevention of accidents or contamination.	2. - Specialized Care Program for Pediatric Hemato-Oncology - Section 3. - Required infrastructure and environmental elements	Excluded in Review		not apropriate quality criteria for paediatric oncology	
		Art. 16.<br>§ 1. The preparation and administration of antitumor medications shall be carried out in accordance with the standards referred to in Chapter III, Section 5 of the Royal Decree of 21 March 2003 setting the standards that the basic oncology care programme and the oncology care programme must meet in order to be recognised.<br>§ 2. All anti-tumor drug treatments for patients of the specialized pediatric hemato-oncology care program who are not staying overnight in the hospital should be provided within the framework of the day hospitalization unit of the specialized care program.	2. - Specialized Care Program for Pediatric Hemato-Oncology - Section 3. - Required infrastructure and environmental elements	Excluded in Review		too specific - country specific	

		Art. 17.§ 1. The specialized pediatric hemato-oncology care program has a stem cell transplantation unit dedicated exclusively to the treatment of children. § 2. The stem cell transplantation unit referred to in paragraph 1 shall satisfy at least the following conditions:<br>1° the unit has sufficient beds set up in individual rooms exclusively for children who need to be isolated as a result of the stem cell transplant;<br>2° the nursing and care staff shall have the necessary evidence of competence and experience in the treatment of paediatric patients, in the care of stem cell transplants with children and shall receive the necessary training and further education in relation to these activities;<br>3° the unit has sufficient qualified staff to be able to carry out the care of the patients, adjusting the number for each patient to the care required by the patient;<br>4° In addition to the nursing supervision of the specialized pediatric hemato-oncology care program, the unit has at least four full-time equivalent nurses.The number shall be adjusted according to the activity level of the stem cell transplantation unit;<br>5° In addition to the medical framework of the specialized pediatric hemato-oncology care program, the unit has at least two full-time equivalent physician-specialists holding the special professional title of physician-specialist in pediatrics and in pediatric hematology and oncology with sufficient experience in stem cell transplantation in children.§ 3. By way of derogation from paragraph 1, the specialised paediatric haemato-oncology care programme shall use a stem cell transplantation unit at the same site in which both adults and children are treated.<br>As regards stem cell transplantation in children, the stem cell transplantation unit referred to in the first paragraph shall fulfil the conditions set out in paragraph 2. § 4. Children belonging to the target group of the specialised care programme and the satellite care programme referred to in Chapter 3 shall, for the purposes of stem cell transplantation, only be treated in a stem cell transplantation unit as referred to in paragraphs 1 to 3.	2. - Specialized Care Program for Pediatric Hemato-Oncology - Section 3. - Required infrastructure and environmental elements	Multidisciplinary team (MDT) and other experts	Stem cell transplant unit<br>Number of paediatric oncology disciplines with multidisciplinary staffing ratios for paediatric oncology		

		Art. 18.The hospital with an approved specialised paediatric haemato-oncology care programme must have:<br>1° a recognised tertiary care programme for children;<br>2° a recognized care program for oncology.For hospitals in which surgical and medical benefits are provided exclusively for children, a written cooperation agreement is concluded with a hospital that has a care program for oncology;<br>3° a recognized intensive care function adapted to the treatment of children;<br>4° an approved pediatric liaison function;<br>5° an approved medical imaging service in which a CT scan has been drawn up and which has doctors with special experience in oncology and pediatrics;<br>6° an approved hospital blood bank with the possibility of irradiation of blood products.If this is not the case, the hospital shall conclude a written cooperation agreement with a hospital that has the intended function;<br>7° an approved hospital pharmacy.<br>By way of derogation from the first paragraph, 1°, it is sufficient for the hospital to operate a tertiary care programme for children in association.	2. - Specialized Care Program for Pediatric Hemato-Oncology - Section 3. - Required infrastructure and environmental elements	Facilities and Networks	Paediatric Intensive care unit<br>Paediatric radiology<br>Laboratories: hematology, hematopathology, clinical chemistry, **transfusion**<br>Pharmacy		
		Art. 19.The specialised care programme may, within the hospital or through a cooperation agreement concluded by the hospital in this respect, call on:<br>1° an approved service in which a magnetic resonance tomograph is drawn up with doctors with special experience in oncology and paediatrics;<br>2° an approved radiotherapy service with doctors with special experience in oncology and paediatrics;<br>3° a pathological anatomy laboratory equipped with the technology of molecular biology;<br>4° an accredited laboratory for clinical biology with the techniques for immunoflow cytometry and molecular biology;<br>5° a laboratory for cytogenetic technology.If this is not the case, the hospital will conclude a written cooperation agreement with an approved genetics centre.	2. - Specialized Care Program for Pediatric Hemato-Oncology - Section 3. - Required infrastructure and environmental elements	Facilities and Networks	Radiation therapy<br>Laboratories: hematology, hematopathology, clinical chemistry, transfusion		

		Art. 20.The radiotherapy service referred to in Article 19(2) shall additionally meet the following conditions for the treatment of patients in the specialised care programme:<br>1° at least one medical specialist holder of the special professional title of medical specialist in radiation oncology of the medical team of the service has sufficient experience in the field of radiotherapy in children;<br>2° the service has adapted equipment, infrastructure, a medical team and technical staff for the treatment of children to carry out treatments with external radiation and brachytherapy;<br>3° there is a smooth cooperation with the anesthesiology department for the performance of radiation treatments under anesthesia in uncooperative children;<br>4° the service has the necessary material for total body irradiation adapted for children in the context of conditioning for transplants.	2. - Specialized Care Program for Pediatric Hemato-Oncology - Section 3. - Required infrastructure and environmental elements	Excluded in Review	Radiation therapy	too specific - criteria for a specific discipline	
		Art. 21.The size, number and nature of the establishment, equipment and equipment are adapted to the number and specific needs of all children.	2. - Specialized Care Program for Pediatric Hemato-Oncology - Section 3. - Required infrastructure and environmental elements	Excluded in Review		given by good clinical practice	
		Art. 22.At least the following materials are available:<br>1° infusion pumps with the possibility of setting a maximum volume to be infused;<br>2° spray pumps;<br>3° cardio-respiratory monitoring;<br>4° saturation meter (with adapted probe);<br>5° blood pressure monitor (with adapted cuff);<br>6° aspiration material;<br>7° aerosol device;<br>8° CPR material for children of all ages, including CPR guidelines;<br>9° the materials necessary for the administration and wetting of oxygen, adapted to the age and needs of the child.	2. - Specialized Care Program for Pediatric Hemato-Oncology - Section 3. - Required infrastructure and environmental elements	Excluded in Review		too specific	

		Art. 23.The medical coordination of the specialised care programme for paediatric haemato-oncology shall be carried out by a specialist physician holding the special professional title of specialist physician in paediatrics and in paediatric haematology and oncology.<br><br>The medical coordinator referred to in the first paragraph shall:<br>1° the general coordination of the specialized care program;<br>2° ensure the link between the specialised care programme and the satellite care programmes with which a cooperation agreement has been concluded;<br>3° monitor the maintenance and compliance with the quality manual, including with regard to the referral of patients;<br>4° ensure cooperation between the various actors of the specialised paediatric haemato-oncology care programme, the children's care programme and the general practitioners responsible for the children;<br>5° coordinating participation in national and international scientific research.<br>The coordinator referred to in the first paragraph shall be full-time and exclusively associated with the care programme. He is appointed by the administrator on the recommendation of the medical department head of the hospital's children's care program.	2. - Specialized Care Program for Pediatric Hemato-Oncology - Section 4. - Medical and non-medical support and expertise - Subsection 1. - Coordination	Excluded in Review		refers to a shared or sattelite care centre, not the main centre	

		Art. 24.The coordinator referred to in Article 23 shall be assisted in the performance of his or her duties by an administrative coordinator who shall be attached to the care programme full-time and exclusively.<br><br>The administrative coordinator referred to in the first paragraph shall:<br>1° recording the data in accordance with the modalities of the quality manual referred to in Article 28;<br>2° transfer of the registered data to the College of Paediatric Hemato-Oncology as referred to in Article 56, and to the Cancer Registry Foundation referred to in Article 45d of Royal Decree No.<br>78 of 10 November 1967 on the pursuit of the health professions;<br>3° participate in the multidisciplinary oncology consultation of the target group and participate in the preparation of its reports.<br>The administrative coordinator shall perform those tasks under the responsibility of the medical coordinator as referred to in Article 23.<br><br>The administrative coordinator is a master's or a bachelor's degree who has a special knowledge of the treatment of children with haemato-oncological diseases and/or clinical studies.<br><br>In addition, he must prove that he has successfully completed training in the field of cancer registration organized by the Cancer Registry Foundation referred to in the second paragraph, 2°.	2. - Specialized Care Program for Pediatric Hemato-Oncology - Section 4. - Medical and non-medical support and expertise - Subsection 1. - Coordination	Multidisciplinary team (MDT) and other experts	Medical secretaries, data manager		
		Art. 25.The nursing coordination of the specialized care program for hemato-oncology is done by a graduate nurse or bachelor of nursing with a special professional title in pediatrics and in neonatology, with an experience of at least 5 years in pediatric hemato-oncology.<br><br>The coordinator referred to in the first paragraph shall be full-time and exclusively associated with the care programme.	2. - Specialized Care Program for Pediatric Hemato-Oncology - Section 4. - Medical and non-medical support and expertise - Subsection 1. - Coordination	Excluded in Review		certification of professionals	

		Art. 26.§ 1. The specialized care program for pediatric hemato-oncology has a medical team consisting of at least 4 full-time equivalent physician-specialists holder of the special professional title of physician-specialist in pediatrics and in pediatric hematology and oncology.<br><br>At least one medical specialist as referred to in the first paragraph is on call 24 hours a day. § 24. The number of medical specialists referred to in the first paragraph shall be adjusted according to the volume of activity of the specialised care programme.	2. - Specialized Care Program for Pediatric Hemato-Oncology - Section 4. - Medical and non-medical support and expertise - Subsection 2. - Medical support	An MDT should consist of representatives from the following disciplines/expertise (disciplines involved depend on the patients' needs):	Paediatric oncologists		
		Art. 27.The specialized care program for pediatric hemato-oncology must be able to call on medical specialists 24 hours a day who have special experience in the treatment of children in the fields of psychiatry, cardiology, nephrology, pneumology, gastroenterology, neurology, endocrinology, neonatology, general surgery, orthopedic surgery, neurosurgery, urology, thoracic surgery, abdominal surgery, surgery of the head and neck, plastic and reconstructive surgery, anesthesia, pain treatment, infectiology and radiotherapy.	2. - Specialized Care Program for Pediatric Hemato-Oncology - Section 4. - Medical and non-medical support and expertise - Subsection 2. - Medical support		Psychosocial care/services<br>Paediatric cardiologist<br>Paediatric nephrologist<br>Paediatric pulmonologist<br>Paediatric gastroenterologist<br>Paediatric neurologist<br>Paediatric endocrinologist<br>Paediatric surgeons<br>Paediatric anaesthesiology Pain management experts<br>Paediatric infectious diseases specialists<br>Radiation oncologists		
		Art. 28.The specialized pediatric hemato-oncology care program has at least 22 full-time equivalent nurses who are adapted to the needs of the patients according to number and qualification.<br><br>At least 50 % of the nursing staff referred to in the first paragraph shall be graduate nurses or bachelors of nursing with a special professional title in paediatrics and in neonatology or in oncology.	2. - Specialized Care Program for Pediatric Hemato-Oncology - Section 4. - Medical and non-medical support and expertise - Subsection 3. - Nursing support		Paediatric oncology nurses	certification of professionals	

		Art. 29.The administration of chemotherapy is carried out exclusively by or under the supervision of graduate nurses or bachelors of nursing with a special professional title in oncology.	2. - Specialized Care Program for Pediatric Hemato-Oncology - Section 4. - Medical and non-medical support and expertise - Subsection 3. - Nursing support	Excluded in Review		certification of professionals	
		Art. 30.The nursing staff has received training in the field of evaluation and treatment of pain in children.	2. - Specialized Care Program for Pediatric Hemato-Oncology - Section 4. - Medical and non-medical support and expertise - Subsection 3. - Nursing support	Excluded in Review		certification of professionals	
		Art. 31.For psychosocial support, the specialised paediatric haemato-oncology care programme must have a psychosocial team composed of at least 2.5 full-time equivalent psychologists, 1.5 full-time equivalent social workers or nurses specialised in social health care or nurses with at least 5 years' experience in social health care on the date of publication of this decision and 2 full-time equivalent pedagogical staff.<br><br>The number of psychosocial counsellors is adapted to the workload of the specialised care programme.<br><br>Psychosocial support should be ensured at all stages of the condition.<br><br>The members of the psychosocial team must have received training in the evaluation and treatment of children with haemato-oncological diseases.	2. - Specialized Care Program for Pediatric Hemato-Oncology - Section 4. - Medical and non-medical support and expertise - Subsection 4. - Other framework	Multidisciplinary team (MDT) and other experts	Number of paediatric oncology disciplines with multidisciplinary staffing ratios for paediatric oncology<br>Psychosocial care/services		

		Art. 32.The specialist paediatric haemato-oncology care programme must have at least 0.5 full-time equivalent dietitian, 1 full-time equivalent physiotherapist or occupational therapist, 0.5 full-time equivalent speech therapist, 1 full-time equivalent hospital pharmacist with additional training in clinical pharmacy and 1.5 full-time equivalent logistics support staff.	2. - Specialized Care Program for Pediatric Hemato-Oncology - Section 4. - Medical and non-medical support and expertise - Subsection 4. - Other framework	Multidisciplinary team (MDT) and other experts	Number of paediatric oncology disciplines with multidisciplinary staffing ratios for paediatric oncology<br>Dieticians<br>Occupational therapists<br>Pharmacists experienced in chemotherapy preparation		
		Art. 33.The specialized pediatric hemato-oncology care program provides continuing training for the staff participating in the activities of the specialized care program.	2. - Specialized Care Program for Pediatric Hemato-OncologySection 4. - Medical and non-medical support and expertise - Subsection 5. - General provision	Excluded in Review		certification of professionals	
		Art. 34.The hospital that has an approved specialized care program for pediatric hemato-oncology shall conclude a written cooperation agreement with a hospital that has a satellite care program for pediatric hemato-oncology as referred to in Chapter 3 if it receives a request to do so.<br><br>Cooperation agreements that do not result in effective referrals and referrals as provided for in the multidisciplinary paediatric haemato-oncology manual referred to in Article 36 shall be considered non-existent.<br><br>The cooperation agreement referred to in the first paragraph shall ensure, inter alia:<br>1° accessible and continuous quality assurance;<br>2° cooperation in the field of national and international scientific research;<br>3° the organisation of joint multidisciplinary paediatric haemato-oncology consultations for patients of the satellite care programme;<br>4° the joint recording of the data relating to the patients of the satellite care programme.	2. - Specialized Care Program for Pediatric Hemato-Oncology - Section 5. -Quality standards	Excluded in Review		refers to a shared or sattelite care centre, not the main centre	

		Art. 35.Within the medical team of the specialized care program, a physician-specialist is designated as liaison officer who coordinates cooperation within the hospital, in particular with the children's care program, as well as with other hospitals, in particular hospitals that are approved for a satellite care program for pediatric hemato-oncology, in the field of care for children with hemato-oncological diseases.	2. - Specialized Care Program for Pediatric Hemato-Oncology - Section 5. -Quality standards			refers to a shared or sattelite care centre, not the main centre	

		Art. 36.The hospital accredited for a specialized pediatric hemato-oncology care program uses a multi-disciplinary pediatric hemato-oncology manual consisting of:<br>1° a list of all medical specialists and all other persons participating in the activities of the specialized care program and their respective functions;<br>2° the multidisciplinary guidelines on diagnosis, multidisciplinary treatment, rehabilitation, follow-up of late effects and palliative care for all haemato-oncological and non-oncological haematological diseases in patients under 16 years of age.<br>In particular, the handbook contains, inter alia, guidelines on the measures to be taken to protect the patient's future fertility;<br>3° the modalities for the cooperation, referral and referral of patients from satellite care programmes for paediatric haemato-oncology and from general hospitals to the specialised paediatric haemato-oncology care programme;<br>4° the modalities of cooperation with one or more satellite care programmes referred to in Chapter 3;<br>5° the modalities of cooperation with other specialized care programs for pediatric hemato-oncology that have particular expertise in specialized diagnostic methods, certain specific oncological pathologies or certain complex treatments or specialized techniques;<br>6° the cooperation modalities with the care program for children of the hospital;<br>7° the referral modalities to oncology care programs in the event that the specialized care program itself cannot offer certain care modalities or when the patients exceed or will exceed the age of the target group of the specialized care program, to ensure, among other things, the follow-up of the late effects in the context of the transition consultation;<br>8° the guidelines and procedures for the prevention and specific treatment of pain in children;<br>9° the modalities of continuing training of the staff associated with the specialised care programme;<br>10° the modalities of cancer registration;<br>11° the modalities of the qualitative evaluation of the activity of the care program.<br>The manual is available in the hospital for inspection by all doctors, nurses and all other healthcare providers, including referring GPs as well as by the inspector of the accrediting authority	2. - Specialized Care Program for Pediatric Hemato-Oncology - Section 5. -Quality standards	Multidisciplinary team (MDT) and other experts	Covered in different sections of the review; manual per se is not a criterion but includes quality criteria wich are alredy covered		

		Art. 37.§ 1. The specialized care program for pediatric hemato-oncology organizes at least one multidisciplinary pediatric hemato-oncology consultation for each patient. § 2. The multidisciplinary paediatric haemato-oncology consultation shall include at least:<br>1° a medical specialist holder of the special professional title of medical specialist in paediatrics and paediatric haematology and oncology;<br>2° a graduate nurse or bachelor's degree in nursing with a special professional title in paediatrics and neonatology;<br>3° a member of the psychosocial team;<br>4° a medical specialist holder of the special professional title of medical specialist in paediatrics;<br>5° a medical specialist holder of the special professional title of medical specialist in pathological anatomy and/or in clinical biology;<br>6° a medical specialist holder of the special professional title of medical specialist in X-ray diagnosis;<br>7° a medical specialist holder of the special professional title of medical specialist in radiation oncology and a medical specialist holder of the special professional title of medical specialist in surgery, if the patient's pathology requires recourse to these specialties.<br>The participants in 1° to 3° are part of the care program. § 3. Each multidisciplinary consultation is presented in a report, which includes: the date on which the consultation took place, the participants in the consultation on the basis of an attendance list and a summary of the outcome of the consultation.	2. - Specialized Care Program for Pediatric Hemato-Oncology - Section 5. -Quality standards	An MDT should consist of representatives from the following disciplines/expertise (disciplines involved depend on the patients' needs):	Paediatric oncologists<br>Paediatric oncology nurses<br>Psychosocial care/services<br>Paediatric pathologist<br>Paediatric radiologists<br>Radiation oncologists		
		Art. 38.Oncological treatment plans shall be drawn up for each patient in accordance with the guidelines of the multidisciplinary paediatric haemaediatric oncology manual as part of the multidisciplinary paediatric haemato-oncology consultation referred to in Article 37.<br><br>If the treatment plan deviates from the guidelines of the multidisciplinary paediatric haemaediatric oncology manual, this shall be explicitly stated in the treatment plan.	2. - Specialized Care Program for Pediatric Hemato-Oncology - Section 5. -Quality standards	Excluded in Review		covered in (treatment) protocols	
		Art. 39.At the end of treatment, a report shall be drawn up for each patient on the follow-up within the specialised care programme for paediatric haemato-oncology, in particular with a view to communicating it to the doctor responsible for the transition consultation referred to in Article 40.	2. - Specialized Care Program for Pediatric Hemato-Oncology - Section 5. -Quality standards	Long-term care	Number/Proportion of survivors of childhood cancer with a survivor care plan<br>Established follow-up structure		

		Art. 40.At the moment that the patient no longer meets the criteria of the target group of the specialized care program of the specialized care program as described in Article 3 during treatment within the specialized care program for pediatric hemato-oncology, a transition consultation is organized between, on the one hand, the treating medical team of the specialized care program and, on the other hand, the medical team of the oncology care program that will care the patient in the future in particular with regard to the late effects of the treatments.<br>A report of this transition consultation is drawn up and transmitted to the patient's general practitioner and the medical team of the oncology care program or any other medical team that observes the long-term follow-up of the patient.	2. - Specialized Care Program for Pediatric Hemato-Oncology - Section 5. -Quality standards	Long-term care	Established transition structure		
		Art. 41.The specialized care program for pediatric hemato-oncology participates in national and international scientific research.	2. - Specialized Care Program for Pediatric Hemato-Oncology - Section 5. -Quality standards	Treatment	Number/Proportion of clinical trial participation		
		Art. 42.§ 1. Each specialized pediatric hemato-oncology care program must participate in a cancer registry, as determined by the Cancer Registry Foundation and the Board of Pediatric Hemato-Oncology as referred to in Article 56.<br><br>The College of Pediatric Hemato-Oncology draws up a model for the cancer registration in which the minimum parameters to be registered are determined that allow to measure the activity and quality of care offered by the specialized care program.<br><br>Referrals between the specialized paediatric haemato-oncology care programmes and the satellite care programmes shall be recorded in the database of the care programme. § 2. Each specialized pediatric hemato-oncology care program should also periodically evaluate the degree of implementation of the multidisciplinary guidelines described in the multidisciplinary pediatric hemato-oncology manual. Based on the periodic evaluation of the implementation rate of these guidelines, a regular re-evaluation of the multidisciplinary paediatric haemato-oncology manual shall be carried out.	2. - Specialized Care Program for Pediatric Hemato-Oncology - Section 6. - Quality monitoring	Facilities and Networks	Childhood cancer registry		

		Art. 43.The satellite care programme for paediatric haemato-oncology shall comply with the same provisions as apply to the paediatric haemaediatric haemato-oncology specialised care programme in terms of target group, nature and content of care.	3. - Satellite Care Program for Pediatric Hemato-Oncology - Section 1. - Target group, nature and content of care	Excluded in Review		refers to a shared or sattelite care centre, not the main centre	
		Art. 44.The satellite care program for pediatric hemato-oncology must comply with the same provisions as apply to the specialized care program for pediatric hemato-oncology with the exception of Article 4, § 3, and with the understanding that in order to be and remain recognized, the treatment of a minimum of 20 new patients must be demonstrated.<br><br>If part of a patient's treatment after an initial diagnosis in a satellite care program is in a specialized pediatric hemato-oncology care program, the patient is counted in the activity level of the satellite care program and the specialized care program, to the extent that part of the treatment takes place in the satellite care program. If this is not the case, the patient is only counted in the specialized care program for pediatric hemato-oncology.	3. - Satellite Care Program for Pediatric Hemato-Oncology - Section 2. - Minimum activity level	Excluded in Review		refers to a shared or sattelite care centre, not the main centre	
		Art. 45.The satellite care programme for paediatric haemato-oncology shall comply with the same provisions as apply to the specialised paediatric haemato-oncology care programme in terms of infrastructure and environmental elements, provided that the hospitalisation unit referred to in Article 7 has a minimum of 6 beds, the satellite care programme shall not have a stem cell transplantation unit as referred to in Article 17 nor shall it have a Paediatric liaison function as referred to in Article 18, 4°.	3. - Satellite Care Program for Pediatric Hemato-Oncology - Section 3. - Required infrastructure and environmental elements	Excluded in Review		refers to a shared or sattelite care centre, not the main centre	
		Art. 46.The satellite care programme for paediatric haemato-oncology shall comply with the same provisions as apply to the specialised paediatric haemato-oncology care programme in terms of coordination, with the exception of Article 24.	3. - Satellite Care Program for Pediatric Hemato-Oncology - Section 4. - Medical and non-medical support and expertise - Subsection 1. - Coordination	Excluded in Review		refers to a shared or sattelite care centre, not the main centre	

		Art. 47.The satellite care programme for paediatric haemato-oncology shall comply with the same provisions as apply to the specialised paediatric haemato-oncology care programme, provided that the medical team consists of at least 2 full-time equivalent medical specialists, holder of the special professional title of specialist physician in paediatrics and in paediatric haematology and oncology.	3. - Satellite Care Program for Pediatric Hemato-Oncology - Section 4. - Medical and non-medical support and expertise - Subsection 2. - Medical support	Excluded in Review		refers to a shared or sattelite care centre, not the main centre	
		Art. 48.The satellite care programme for paediatric haemato-oncology shall comply with the same provisions as apply to the specialised paediatric haemato-oncology care programme in terms of nursing care, provided that the nursing team consists of at least 10 full-time equivalent nurses.	3. - Satellite Care Program for Pediatric Hemato-Oncology - Section 4. - Medical and non-medical support and expertise - Subsection 3. - Nursing support	Excluded in Review		refers to a shared or sattelite care centre, not the main centre	
		Art. 49.The satellite care programme for paediatric haemato-oncology shall comply with the same provisions as apply to the specialised paediatric haemato-oncology care programme with regard to psychosocial support, provided that the psychosocial support team is composed of at least 1 full-time equivalent psychologist, 1 full-time equivalent social worker or nurse specialising in social health care or nurse with a experience of at least 5 years in social health care on the date of publication of this decision and 1 full-time equivalent pedagogical employee.	3. - Satellite Care Program for Pediatric Hemato-Oncology - Section 4. - Medical and non-medical support and expertise - Subsection 4. - Other framework	Excluded in Review		refers to a shared or sattelite care centre, not the main centre	
		Art. 50.The satellite care program for pediatric hemato-oncology should also have at least 0.5 full-time equivalent dietitian, 0.5 full-time equivalent physiotherapist or occupational therapist, 0.5 full-time equivalent speech therapist, 0.5 full-time equivalent hospital pharmacist with additional training in clinical pharmacy and 0.5 full-time equivalent logistics support staff.	3. - Satellite Care Program for Pediatric Hemato-Oncology - Section 4. - Medical and non-medical support and expertise - Subsection 4. - Other framework	Excluded in Review		refers to a shared or sattelite care centre, not the main centre	

		Art. 51.The satellite care programme for paediatric haemato-oncology provides continuing training for staff participating in the activities of the satellite care programme.	3. - Satellite Care Program for Pediatric Hemato-Oncology - Section 4. - Medical and non-medical support and expertise - Subsection 5. - General provision	Excluded in Review		refers to a shared or sattelite care centre, not the main centre	
		Art. 52.The hospital that has an approved satellite care program for pediatric hemato-oncology shall conclude a written cooperation agreement with at least one specialized pediatric hemato-oncology care program as referred to in Chapter 2.<br><br>Cooperation agreements that do not result in effective referrals and referrals as provided for in the multidisciplinary paediatric haemato-oncology manual referred to in Article 36 shall be considered non-existent.<br><br>In addition to the provisions referred to in Article 33, the cooperation agreement shall contain provisions concerning the referral of patients in need of a stem cell transplant.	3. - Satellite Care Program for Pediatric Hemato-Oncology - Section 4. - Medical and non-medical support and expertise - Section 5. -Quality standards	Excluded in Review		refers to a shared or sattelite care centre, not the main centre	
		Art. 53.The hospital that has a satellite care program for pediatric hemato-oncology enters into one or more written cooperation agreements with a hospital that is recognized for a specialized care program for pediatric hemato-oncology and that has a pediatric liaison function.	3. - Satellite Care Program for Pediatric Hemato-Oncology - Section 4. - Medical and non-medical support and expertise - Section 5. -Quality standards	Excluded in Review		refers to a shared or sattelite care centre, not the main centre	
		Art. 54.The satellite care programme for paediatric haemato-oncology shall otherwise comply with the same provisions as apply to the specialised paediatric haemato-oncology care programme set out in Articles 34 to 40 in terms of quality standards.	3. - Satellite Care Program for Pediatric Hemato-Oncology - Section 5. -Quality standards	Excluded in Review		refers to a shared or sattelite care centre, not the main centre	
		Art. 55.The satellite care programme for paediatric haemato-oncology shall comply with the same quality monitoring provisions as apply to the paediatric haemato-oncology specialised care programme.	3. - Satellite Care Program for Pediatric Hemato-Oncology - Section 6. - Quality monitoring	Excluded in Review		refers to a shared or sattelite care centre, not the main centre	

			4. - Satellite Care Program for Pediatric Hemato-Oncology - Section 4. - Medical and non-medical support and expertise - Section 6. - Quality monitoring				
**France**	Document pediatric oncology: Accreditation criteria for cancer treatment of children and adolescents under 18 years of age[11]	**I. In addition to the obligations attached to authorizations under Article R. 6123-87 of the French Public Health Code, the following quality criteria are met:**					
		1. The establishment has at its disposal or by agreement :<br>- pediatric imaging facilities, with the possibility of deep sedation;<br>- an operating area with the equipment, medical devices and skills required to care for children;<br>- a pediatric intensive care unit.<br>The procedures for initial patient packaging and transfer to this unit are formalized in writing.		Facilities and Networks	Paediatric radiology<br>Paediatric anaesthetics<br>Paediatric surgery a<br>Intensive care unit		
		2. The establishment guarantees that each patient can benefit from a fertility preservation strategy that includes access, on site or by agreement, to a structure authorized to carry out autologous preservation of gametes and germ tissue.		Existence of supportive care guidelines including supportive care (guidelines) for	Fertility (preservation) discussion		
		3. The facility is a member of an inter-regional hospital organization for pediatric oncology, identified by the French National Cancer Institute. The organization's mission is to organize and run inter-regional pediatric multidisciplinary consultations, which replace the multidisciplinary consultations covered by the accreditation criteria adopted on December 20, 2007		Multidisciplinary team (MDT) and other experts		too specific - country specific	

		4. The establishment submits the file of each patient under 18 years of age to the inter□regional paediatric multidisciplinary consultation meeting, held under the following conditions:<br>- each patient's file is recorded and always discussed, including in the event of therapeutic reorientation or relapse;<br>- the doctor who submits the patient's file is the one who will provide or coordinate the treatment.<br>In clinical situations requiring the administration of an initial emergency treatment, the discussion takes place after this treatment has been administered.		Multidisciplinary team (MDT) and other experts		too specific - country specific	
		5. The pediatric interregional multidisciplinary consultation meeting :<br>- validates the therapeutic indication for surgery, chemotherapy or radiotherapy,<br>- proposes the technical facilities, without prejudice to the patient's and family's freedom of choice, that it considers appropriate for the planned procedures and continuity of care, as well as the care pathway based on existing channels.		Multidisciplinary team (MDT) and other experts		too specific - country specific	
		6. For adolescents aged 16 to 18, the inter-regional pediatric pluridisciplinary consultation meeting may propose treatment in a facility that treats cancer in adult patients, within an establishment authorized for this type of care. Such referral requires the explicit agreement of the patient and his or her family. All criteria apply to this structure, with the exception of criteria 1, 3 and 14, 15 and 17. Any change in treatment is discussed at an inter-regional pediatric consultation meeting.				too specific - country specific	
		7. The facility organizes the reception and presence of parents and visits from siblings, and formalizes an organization to ensure the parents' accommodation.		Excluded in Review		does not measure quality (in single centres)	
		8. The facility provides psychological care for the patient and, where appropriate, for family and friends.		Existence of supportive care guidelines including supportive care (guidelines) for	Psychological or psychosocial care, including provision of/information about social care		
		9. The establishment assesses the family's social needs, informs them of these and assists them in applying for social benefits.		Existence of supportive care guidelines including supportive care (guidelines) for	Psychological or psychosocial care, including provision of/information about social care		

		10. The establishment provides the conditions for maintaining schooling and setting up an educational project.		Existence of supportive care guidelines including supportive care (guidelines) for	Provision of school education		
		11. The facility organizes and coordinates the continuity of patient care in conjunction with local facilities and professionals working in the home.		Long-term care			
		**II. The criteria by therapeutic practice, adopted by deliberation of the Board of Directors of the Institut National du Cancer on December 20, 2007, apply to pediatric oncology under the following conditions:**					
		**Cancer surgery**					
		12. General accreditation criteria for cancer surgery nos. 1 to 8 apply to the care of children and adolescents under 18.		Excluded in Review		too specific - country specific	
		**Chemotherapy**					
		13. General approval criteria for the practice of chemotherapy n° 1 to 4, 6 to 8 and 10 to 15 apply to the care of children and adolescents under 18.		Excluded in Review		too specific - country specific	
		14. An anesthetist experienced in pediatric anesthesia is available to set up long-term intravenous devices.		An MDT should consist of representatives from the following disciplines/expertise (disciplines involved depend on the patients' needs):	Paediatric anaesthesiology		
		15. The establishment includes at least one pediatrician with the training or experience required by regulations governing cancer treatment.		An MDT should consist of representatives from the following disciplines/expertise (disciplines involved depend on the patients' needs):	Paediatric oncologists		
		**External radiotherapy**					
		16. The criteria for the practice of external radiotherapy apply to the care of children and adolescents under the age of 18.		Facilities and Networks	Radiation therapy		

		17. Treatment of patients under the age of 16, with the exception of total body irradiation, is carried out in radiotherapy centers:<br>- including at least one radiotherapist who regularly participates in the interregional pediatric multidisciplinary consultation meeting;<br>- performing at least 12 treatments per year; only treatments performed on different patients aged under 16, excluding total body irradiation and palliative treatments, are counted.		Facilities and Networks	Radiation therapy		
		18. Palliative treatments can be carried out in all radiotherapy centers, on the advice of the inter-regional pediatric multidisciplinary consultation meeting		Existence of supportive care guidelines including supportive care (guidelines) for	Palliative care (including bereavement)		
**France**	Decree no. 2022-689 of April 26, 2022 on the conditions for setting up cancer treatment facilities[12]	"III. - Mention C providing oncology surgery for children and adolescents under the age of eighteen.	General provisions		Paediatric surgery		
		"3o Mention C providing the same treatments for children and adolescents under the age of eighteen, in addition to external radiotherapy or brachytherapy treatments for adults.	General provisions		Radiation therapy		
		Mention C providing systemic drug treatment for cancer in children and adolescents under the age of eighteen, including intensive drug treatment leading to foreseeable aplasia lasting more than eight days and the management of this foreseeable aplasia, coordination of the entire care pathway for the minor patient under care, and expertise and referral in pediatric oncology for other health establishments and outpatient medicine contributing to this care pathway.	General provisions			covered in (treatment) protocols	

		"Art. R. 6123-91-3. - I. - Without prejudice to the application of the provisions of I of article R. 6123-91, the establishment authorized to treat cancer in children and adolescents under the age of eighteen is a member of an inter□regional pediatric oncology referral hospital organization, identified by the Institut national du cancer in application o f article L. 1415-2. In particular, this organization is responsible for organizing and ensuring inter-regional multidisciplinary consultation for patients under the age of eighteen, which meets the same obligations as the multidisciplinary consultations mentioned i n article R. 6123-91-1.<br><br>"Art. R. 6123-91. - I. - Authorization may only be granted if the applicant is a member of the specific regional cancer program recognized by the Institut national du cancer.<br>"II. - The authorization holder satisfies the approval criteria defined by the Institut National du Cancer in application of 2o of article L. 1415-2 with regard to the quality of cancer care.<br>"Art. R. 6123-91-1. - Authorization may be granted only if the applicant :<br>"1o Has an organization, set up where appropriate jointly with other cancer treatment authorization holders, which ensures that each patient is informed of the diagnosis and of a therapeutic proposal based on multidisciplinary consultation in accordance with the care guidelines defined by the Institut national du cancer in application of 2o of article L. 1415-2 and translated into a personalized care program given to the patient;<br>"2o Ensures the organization of the multidisciplinary consultations mentioned in 1o. When the applicant or the holder of t h e cancer treatment activity d o e s not perform all of the cancer treatment modalities and therapeutic procedures respectively mentioned i n article R. 6123-86-1 and 1o o f article R. 6123-90-2, multidisciplinary consultation is organized with other authorization holders performing at least the cancer treatment modalities mentioned in article R. 6123-86-1.	Cross-disciplinary quality measures in cancer research	Multidisciplinary team (MDT) and other experts			
		"II. - Treatment proposals for children and adolescents under the age of eighteen are systematically submitted t o an inter-regional multidisciplinary pediatric oncology consultation meeting, in compliance with the conditions set out in the previous paragraph.	Cross-disciplinary quality measures in cancer research	Multidisciplinary team (MDT) and other experts	MDT established, including regularly scheduled MDT conferences		

		"III. - For adolescents between the a g e s o f sixteen and eighteen, the inter-regional paediatric multidisciplinary consultation meeting may propose treatment in a facility authorized to treat cancer in adult patients. Such referral requires t h e informed and explicit agreement of the patient and his or her family.	Cross-disciplinary quality measures in cancer research		Established transition structure		
		The provisions o f articles R. 6123-92-9 (1) and R. 6123-14-2 (2) and the Institut National du Cancer's approval criteria for cancer treatment in children and adolescents under the age of eighteen then apply to the structure providing the treatment. Any change in treatment is discussed at an inter-regional pediatric multidisciplinary consultation meeting.<br>"Art. R. 6123-92-9. - For the post-operative management of t h e patients they treat, the holder o f the authorization for thoracic oncology surgery with A2 mention mentioned in I of article R. 6123-87-1, the holder of the authorization for complex oncology surgery with B mention mentioned in II of article R. 6123-87-1 and the holder of the authorization for surgery on children and adolescents under the age of eighteen with C mention mentioned in III<br>of this same article also have, on site:<br>"1o A continuous monitoring unit ;<br>"2o The organization of continuity of care for these patients, guaranteeing the permanent presence, on site or on call, of a physician specialized in anesthesia-intensive care or intensive care medicine.<br>"When providing care to patients with cancer of the esophagus or gastro-esophageal junction, the holder of a complex digestive oncology surgery authorization with B1 designation also guarantees access, on the site or in adjacent buildings, to an intensive care unit referred to in 1 or 2 o f article R. 6123-34-1, capable of managing acute respiratory syndromes, or to a resuscitation unit.<br>"The holder of a B2 authorization for complex thoracic oncology surgery also guarantees access to an intensive care unit on site or in adjacent buildings.<br>"When the intensive care unit or the resuscitation unit located in the neighboring building is owned by another legal entity, an agreement is mandatory.	Cross-disciplinary quality measures in cancer research	Multidisciplinary team (MDT) and other experts	MDT established, including regularly scheduled MDT conferences		

		"Art. R. 6123-93-7. - Notwithstanding the provisions of article R. 6123-88-1, palliative treatments for children and adolescents under the age of eighteen may be provided in external radiotherapy centers with A or B classification, after consultation with the inter-regional pediatric multidisciplinary consultation meeting.<br>"Art. R. 6123-88-1. - The "External radiotherapy, brachytherapy" modality includes the following entries:<br>"1o Mention A providing external radiotherapy treatment for adults ;<br>"2o Mention B for brachytherapy treatments in adults ;<br>"3o Mention C providing the same treatments for children and adolescents under the age of eighteen, in addition to external radiotherapy or brachytherapy treatments for adults.	Special provisions for external radiotherapy and brachytherapy	Facilities and Networks			
		"Art. R. 6123-94-1-1. - Establishments authorized to provide systemic cancer drug treatments with a C rating must meet the obligations imposed on holders of medical authorizations for children and adolescents mentioned in articles R. 6123-3 and R. 6123-10.	Special provisions for systemic drug treatments for cancer	Excluded in Review		does not measure quality (in single centres)	
**France**	Decree no.2022-693 of April 26, 2022 on the technical operating conditions for cancer treatment services[13]	**"Cancer treatment activity**					
		"Art. D. 6124-131. - I. - The therapeutic plan envisaged for each cancer patient under care, as well as significant changes in therapeutic orientation, including the discontinuation of cancer treatment, are the subject of a collegial discussion at a multidisciplinary consultation meeting. [...] "In clinical situations requiring the administration of emergency treatment in children or adolescents, discussion at an inter-regional pediatric oncology multidisciplinary consultation meeting takes place after the treatment has been administered.	Cross-disciplinary quality measures in cancer research	Multidisciplinary team (MDT) and other experts	MDT established, including regularly scheduled MDT conferences		

		"Art. D. 6124-131-5. - I. - The holder of an authorization to treat cancer in children and adolescents under the age of eighteen shall take all necessary steps to ensure that :<br>"1o Where appropriate, to support and encourage the continuation of schooling or education and the implementation of an educational project ;<br>"2o If necessary, psychological support for parents and relatives;<br>"3o Reception and, if necessary, assistance with accommodation for parents, either in a parent-child room or by other means.	Cross-disciplinary quality measures in cancer research	Existence of supportive care guidelines including supportive care (guidelines) for	Provision of school education<br>Psychological or psychosocial care, including provision of/information about social care		
		"II. - The holder of a cancer treatment authorization for the care of children and adolescents under the age of eighteen ensures the collection and analysis of data from professional practices, with a view to improving practices and managing risks.	Cross-disciplinary quality measures in cancer research		Number/Proportion of clinical trial participation<br>Childhood cancer registry		
		"Art. D. 6124-132. - I. - The holder of the surgical oncology authorization has available on the site : "1o At least one inpatient area for unscheduled treatmen to patients, if required; "2o At least one interventional sector for oncology surgery. "II. - The interventional sector of the holder of a n authorization with mention C mentioned in III of article R. 6123-87-1 includes equipment and medical devices adapted to the care of children. "The holder of an authorization with mention C has access, on site or by agreement, to imaging adapted to children, with the possibility of deep sedation.	Conditions specific to the "oncological surgery" modality	Excluded in Review		too specific - criteria for a specific discipline	
		III. - The holder of the surgical oncology authorization has an organization on site that allows for minimally invasive interventional procedures for the following indications provided for in I or II o f article R. 6123-87-1:<br>[…]<br>Oncological surgery on children and adolescents with the mention C.	Conditions specific to the "oncological surgery" modality	Facilities and Networks	Paediatric surgery		
		"The holder of an authorization for oncological surgery on children and adolescents under the age of 18 with the C designation has surgeons who hold a qualification in the specialty in which they operate or a specialist qualification in pediatric surgery, as well as nursing staff who are competent in oncology or have experience in the management of childhood cancer.	Conditions specific to the "oncological surgery" modality	An MDT should consist of representatives from the following disciplines/expertise (disciplines involved depend on the patients' needs):	Paediatric surgeons<br>Paediatric oncology nurses		

		"Art. D. 6124-134-5. - I. - The consultation for a first prescription of a systemic drug treatment for cancer mentioned in 2 of article R. 6123-131, is carried out, on the authorized site, during a one-to-one interview with the patient, by a prescribing physician from the team of the holder of the authorization for systemic drug treatments for cancer practicing according to the titles or qualifications mentioned in articles D. 6124-134-1 and D. 6124-134-2 when the treatment concerns adults, and article D. 6124-134-3 when the treatment concerns children and adolescents under 18, subject to the situation mentioned in II of article R. 6123-91-3. "This individual interview may be carried out by teleconsultation or advanced consultation in accordance with the provisions of article R. 6123-94.	Conditions specific to the modality: systemic drug treatments for cancer"	Excluded in Review		does not measure quality (in single centres)	
**Poland**	Standards of the department of pediatric oncology and hematology[14]	In order to maintain this level of services in the treatment of childhood cancer, in addition to the appropriate level of funding, the following conditions must be met [1-6]:					
		1. Adequate early diagnosis of the disease, which is associated with a lower clinical stage and significantly higher chances of cure. Achieving this condition is possible only with the cooperation of primary con- tact p2. The final diagnosis and treatment of children with cancer must be carried out only in highly specialized centers for pediatric hematology and oncology, corresponding to standards and having appropriate premises, equipment and medical staff: **experienced in conducting chemotherapy pediatric oncologists/hematologists, nurses, psychologists, educators, social workers.** It is necessary to have round-the-clock cooperation of doctors of other specialties (e**specially pediatric surgeons, anesthesiologists, ENT specialists**) and availability of **imaging and laboratory tests.** Full a**vailability of blood prepara- tion and radiation therapy i**s also essential.hysicians (**pediatricians, family doctors**) with knowledge of the early signs of childhood cancer and the possibility of referral to an appropriate regional pediatric oncology center in the area.		Multidisciplinary team (MDT) and other experts	Paediatric oncologists<br>Paediatric oncology nurses<br>Psychosocial care/services<br>Ward teachers<br>Paediatric surgeons<br>Paediatric anaesthesiology/- gists<br>Paediatric radiology<br>Laboratories: hematology, hematopathology, clinical chemistry, transfusion<br>Radiation therapy		

		2. The final diagnosis and treatment of children with cancer must be carried out only in highly specialized centers for pediatric hematology and oncology, corresponding to standards and having appropriate premises, equipment and medical staff: **experienced in conducting chemotherapy pediatric oncologists/hematologists, nurses, psychologists, educators, social workers.** It is necessary to have round-the-clock cooperation of doctors of other specialties (**especially pediatric surgeons, anesthesiologists, ENT specialists**) and availability of imaging and laboratory tests. Full **availability of blood prepara- tion and radiation therapy** is also essential.			Paediatric oncologists<br>Paediatric oncology nurses<br>Psychosocial care/services<br>Ward teachers<br>Paediatric surgeons<br>Paediatric anaesthesiology/- gists<br>Paediatric radiology<br>Laboratories: hematology, hematopathology, clinical chemistry, transfusion<br>Radiation therapy		
		The European Commission in 2017 implemented a decision to establish European Reference Networks, including the European Reference Network for Pediatric Oncology (ERN PaedCan; European Reference Network). A prerequisite for joining the Network is, among other things, meeting certain standards of care for a child with cancer. The Polish Society of Pediatric Oncology and Hematolo- gy counts on the approval of these standards as binding in Poland by the Minister of Health, by institutions that finance health services, and, consequently, a l s o by hospital di- rections. As a result, all children with cancer in Poland should be treated only in those wards that meet such standards, as only in such conditions can modern treatment methods be used optimally.					
		1. He provides modern high-dose chemotherapy for all childhood cancers in accordance with current therapeutic protocols (leukemias, lymphomas, solid tumors).	I. Conditions that the hematology department must meet and/or pediatric oncology	Excluded in Review		covered in (treatment) protocols	
		2. Annually, at least 30 new children with cancer are diagnosed and treated, and patients are monitored after treatment.	I. Conditions that the hematology department must meet and/or pediatric oncology	Volume and Numbers	Number of cases per year and provider/clinic		

		3. It has premises of a standard corresponding to international standards and those recommended by the Polish Society of Pediatric Oncology and Hematology. The inpatient part should have:	I. Conditions that the hematology department must meet and/or pediatric oncology				
		3.1. 1-2-bed patient rooms.	I. Conditions that the hematology department must meet and/or pediatric oncology	Excluded in Review		does not measure quality (in single centres)	
		3.2. Each room with sanitary facilities.	I. Conditions that the hematology department must meet and/or pediatric oncology	Excluded in Review		does not measure quality (in single centres)	
		3.3. The ability to provide a full sanitary regime for each patient in neutropenia.	I. Conditions that the hematology department must meet and/or pediatric oncology	Supportive care: Febrile neutropenia (F&N)			
		3.4. Equipment to monitor para- meters of vital signs in patients requiring such management.	I. Conditions that the hematology department must meet and/or pediatric oncology	Excluded in Review			

		4. It has a separate part for day treatment (the Day Care Unit) in the form of separate rooms with a separate recovery room and with the possibility of chemotherapy and blood product treatment.	I. Conditions that the hematology department must meet and/or pediatric oncology	Facilities and Networks			
		5. It runs an outpatient clinic that enables it to provide continuity of care for treated children.	I. Conditions that the hematology department must meet and/or pediatric oncology				
		6. In addition to the equipment typical of general pediatric wards, it must be equipped with an adequate number of syringe and volume infusion pumps.	I. Conditions that the hematology department must meet and/or pediatric oncology	Excluded in Review		too specific	
		7. It has access to full laboratory and imaging diagnostics around the clock, as well as provision for blood products.	I. Conditions that the hematology department must meet and/or pediatric oncology	Facilities and Networks	Laboratories: hematology, hematopathology, clinical chemistry, transfusion Paediatric radiology		
		8. Cytotoxic drugs and extra-literal nutrition fluids are prepared by the hospital pharmacy.	I. Conditions that the hematology department must meet and/or pediatric oncology	Facilities and Networks	Pharmacy		
		9. It has the ability to carry out full diagnostics of cancer and hematopoietic diseases in children and adolescents in accordance with **current requirements.**	I. Conditions that the hematology department must meet and/or pediatric oncology	Excluded in Review		too unspecific/broad	

		10. He has the full cooperation of pediatric surgery, intensive care and other specialties around the clock.	I. Conditions that the hematology department must meet and/or pediatric oncology	Facilities and Networks	Paediatric surgery Paediatric Intensive care unit		
		11. He is provided with access to radiation therapy.	I. Conditions that the hematology department must meet and/or pediatric oncology	Facilities and Networks	Radiation therapy		
		12. It employs appropriate professional staff with practical experience in pediatric hematology/oncology.	I. Conditions that the hematology department must meet and/or pediatric oncology	An MDT should consist of representatives from the following disciplines/expertise (disciplines involved depend on the patients' needs):	Paediatric oncologists Paediatric oncology nurses		
		13. It has a quality assurance system, including Standard Operating Procedures available in written or electronic form.	I. Conditions that the hematology department must meet and/or pediatric oncology	Excluded in Review		given by good clinical practice	
		1. The following benchmarks should be considered for determining staffing in pediatric onco- logy and hematology centers:	Ii. Staff working in the department of hematology and/or pediatric oncology				
		1.1. For staff on inpatient wards: average annual occupancy of oncology beds (number of days of care divided by 365).	Ii. Staff working in the department of hematology and/or pediatric oncology	Volume and Numbers	Number of cases per year and provider/clinic		
		1.2. For staff in the day ward and outpatient clinic: the number of admissions to treat sick children per year.	Ii. Staff working in the department of hematology and/or pediatric oncology	Volume and Numbers	Number of cases per year and provider/clinic		
		2. This should take into account the special in□tensity of treatment provided both i n t h e inpatient setting and in day wards and in the pediatric oncology outpatient clinic. The intensity of treatment in an inpatient setting may be comparable to intensive care units, as care for patients u n d e r g o i n g i n t e n s i v e chemotherapy often requires minute supervision throughout the day.	Ii. Staff working in the department of hematology and/or pediatric oncology				

		3. The following groups of employees should be employed in the hematology and/or pediatric oncology department:	Ii. Staff working in the department of hematology and/or pediatric oncology	An MDT should consist of representatives from the following disciplines/expertise (disciplines involved depend on the patients' needs):			
		3.1. Manager - Head of Department.	Ii. Staff working in the department of hematology and/or pediatric oncology	An MDT should consist of representatives from the following disciplines/expertise (disciplines involved depend on the patients' needs):	Paediatric oncology practitioner-in-charge/lead clinician (also with expertise in late effects)		
		3.2. Doctors.	Ii. Staff working in the department of hematology and/or pediatric oncology	An MDT should consist of representatives from the following disciplines/expertise (disciplines involved depend on the patients' needs):	Paediatric oncologists		
		3.3. Nurses.	Ii. Staff working in the department of hematology and/or pediatric oncology	An MDT should consist of representatives from the following disciplines/expertise (disciplines involved depend on the patients' needs):	Paediatric oncology nurses		
		3.4. Psychologists.	Ii. Staff working in the department of hematology and/or pediatric oncology	An MDT should consist of representatives from the following disciplines/expertise (disciplines involved depend on the patients' needs):	Psychosocial care/services		

		3.5. Social workers.	Ii. Staff working in the department of hematology and/or pediatric oncology	An MDT should consist of representatives from the following disciplines/expertise (disciplines involved depend on the patients' needs):	Psychosocial care/services		
		3.6. Nutritionist.	Ii. Staff working in the department of hematology and/or pediatric oncology	An MDT should consist of representatives from the following disciplines/expertise (disciplines involved depend on the patients' needs):	Dieticians		
		3.7. Pedagogues.	Ii. Staff working in the department of hematology and/or pediatric oncology	An MDT should consist of representatives from the following disciplines/expertise (disciplines involved depend on the patients' needs):	Ward teachers		
		3.8. Play therapy workers.	Ii. Staff working in the department of hematology and/or pediatric oncology	An MDT should consist of representatives from the following disciplines/expertise (disciplines involved depend on the patients' needs):	Activity/play therapy staff		
		3.9. Physiotherapists.	Ii. Staff working in the department of hematology and/or pediatric oncology	An MDT should consist of representatives from the following disciplines/expertise (disciplines involved depend on the patients' needs):	Physiotherapists.		

		3.10. Medical technicians.	Ii. Staff working in the department of hematology and/or pediatric oncology	An MDT should consist of representatives from the following disciplines/expertise (disciplines involved depend on the patients' needs):	Laboratory technicians		
		3.11. Medical secretaries and documentarians.	Ii. Staff working in the department of hematology and/or pediatric oncology	An MDT should consist of representatives from the following disciplines/expertise (disciplines involved depend on the patients' needs):	Medical secretaries and data managers		
		4. The head-Ordinator of the pediatric hema□tology and/or oncology department should be a physician with many years of professional practice in pediatric on- cology and hematology, with specialization in this field.	Ii. Staff working in the department of hematology and/or pediatric oncology	An MDT should consist of representatives from the following disciplines/expertise (disciplines involved depend on the patients' needs):	Paediatric oncology practitioner-in-charge/lead clinician (also with expertise in late effects)(13, 28)		
		5. Staffing in the inpatient ward - the standards for staffing in the inpatient ward of pediatric hematology and/or oncology are shown in Table I (based on the standards of Eu- ropean Union countries).	Ii. Staff working in the department of hematology and/or pediatric oncology	Multidisciplinary team (MDT) and other experts	Number of paediatric oncology disciplines with multidisciplinary staffing ratios for paediatric oncology		
		Table I. Staffing norms in pediatric oncology and hematology departments: inpatient stay Table I. Required employment in in-patient pediatric on□cology and hematology ward. Professional group Required employment standards Doctors 0.34/1 bed, Nurses 1.75/1 bed, Psychosocial staff 0.2/1 bed, Medical technicians 0.2/1 bed, Physiotherapists 0.1/1 bed, Secretaries, documentarians 0.2/1 bed, Nutritionist, educators, play therapy workers Available according to age structure and patient needs	Ii. Staff working in the department of hematology and/or pediatric oncology	Multidisciplinary team (MDT) and other experts	Number of paediatric oncology disciplines with multidisciplinary staffing ratios for paediatric oncology		

		5.1. Doctors. review pediatric 59 Jerzy Kowalczyk, Danuta Perek, Valentina Balwierz et al. 2018/Vol. 47/No. 2 There should be 0.34 physician FTEs for each average annual b e d occupancy in pediatric oncology. This ratio is based on the lowest norms used in EU countries and takes into account direct diagnostic a n d treatment procedures and patient c a r e , as well as the time allocated for multidisciplinary discussions regarding patients, and for expanding one's own knowledge. Consideration should also be given to the need for very detailed and repeated discussions with patients and their families. In the treatment with highly toxic preparations, w h i c h are part of chemotherapy, it is necessary to ensure absolute safety. This involves a correspondingly increased time commitment - something that has not been sufficiently taken into account so far.	Ii. Staff working in the department of hematology and/or pediatric oncology	Multidisciplinary team (MDT) and other experts	Number of paediatric oncology disciplines with multidisciplinary staffing ratios for paediatric oncology		
		5.2. Nursing staff. 1.75 FTE per 1 average annual occupied bed in a pediatric hematology and/or oncology unit. In units where treatment is carried out under sterile conditions (bone marrow transplantation units), it is necessary to introduce a 3:1 ratio and the total number of nursing staff should be increased accordingly	Ii. Staff working in the department of hematology and/or pediatric oncology	Multidisciplinary team (MDT) and other experts	Number of paediatric oncology disciplines with multidisciplinary staffing ratios for paediatric oncology		
		5.3. Psychosocial care staff. The staff of the psychosocial team (psy□chologists, educators, social workers, play therapy staff, medical supervisor) should be employed at a rate of 1 FTE per 5 average annual occupancy beds in the ward, or 0.2 FTE per 1 bed.	Ii. Staff working in the department of hematology and/or pediatric oncology	Multidisciplinary team (MDT) and other experts	Number of paediatric oncology disciplines with multidisciplinary staffing ratios for paediatric oncology		
		5.4. Medical and technical staff. 1 FTE per 5 average annual bed occupancy, or 0.2 FTE per bed. This staff is needed to perform specialized laboratory tests on patients with cancer and hematopoietic diseases, such as cy□tomorphologic, immunologic and gene tests on bone marrow, cerebrospinal fluid, drug level determinations, blood coagulation tests and others. Most of these laboratory tests are performed by the Hospital's Central Laboratory, but the workload standards for this group of employees must be taken into account, depending on the work intensity of the hematology/pediatric oncology department.	Ii. Staff working in the department of hematology and/or pediatric oncology	Multidisciplinary team (MDT) and other experts	Number of paediatric oncology disciplines with multidisciplinary staffing ratios for paediatric oncology		

		5.5. Physiotherapists. 1 position per 10 average annual bed occupancy, or 0.1 FTE per bed.	Ii. Staff working in the department of hematology and/or pediatric oncology	Multidisciplinary team (MDT) and other experts	Number of paediatric oncology disciplines with multidisciplinary staffing ratios for paediatric oncology		
		5.6. Nutritionist.	Ii. Staff working in the department of hematology and/or pediatric oncology	Multidisciplinary team (MDT) and other experts	Number of paediatric oncology disciplines with multidisciplinary staffing ratios for paediatric oncology		
		5.7. Medical secretaries and dock workers. 1 position per 5 average annual occupancy beds of the pediatric hematology and oncology department, or 0.2 FTE per bed. With the very in- tensive treatment of cancer patients, it becomes extremely important to have very accurate and ongoing documentation. This is very time-consuming. In addition, there is a need to maintain documentation for financial settlements of services and this should be handled by people familiar with the specifics of the pediatric hematolo- gy and oncology department. It is unacceptable to use the working time of doctors or nurses to prepare this documentation. It should be carried out in consultation with and under the direction of the doctor, but performed by a medical secretary or documentalist. Today, virtually all therapeutic protocols are conducted in the form of academic, non-commercial clinical trials, and as a r e s u l t , it is necessary to report clinical data, adverse events and reports to the study coordinator within a statutorily defined time frame, and these time-consuming tasks must be performed by medical secretaries or documentation staff.	Ii. Staff working in the department of hematology and/or pediatric oncology	Multidisciplinary team (MDT) and other experts	Number of paediatric oncology disciplines with multidisciplinary staffing ratios for paediatric oncology		
		6. Staffing standards for a day unit or outpatient clinic. Staffing standards for a day unit or outpatient clinic for pediatric hematology/oncology are shown in Table II. Within the entire pediatric hematology and on- cology department, it would also be advisable to hire 1 IT specialist for statistical data processing and electronic processing.	Ii. Staff working in the department of hematology and/or pediatric oncology	Multidisciplinary team (MDT) and other experts	Number of paediatric oncology disciplines with multidisciplinary staffing ratios for paediatric oncology		

		Table II. Employment norms in out-patient pediatric oncology and hematology wards: day stay and outpatient<br>Table II. Required employment in out-patient pediatric oncology and hematology wards<br>Professional group<br>Required<br>employment<br>standards<br>Doctors 1.14/1500 tips<br>Nurses 1.05/1500 tips<br>Psychosocial staff 0.5/1500 tips<br>Medical technicians 0.6/1500 tips<br>Physiotherapists 0.2/1500 tips<br>Secretaries, documentarians 0.6/1500 tip	Ii. Staff working in the department of hematology and/or pediatric oncology	Multidisciplinary team (MDT) and other experts	Number of paediatric oncology disciplines with multidisciplinary staffing ratios for paediatric oncology		

Supplemental Table S4: Availability of different types of care by country

	N centres	Allogeneic SCT	Any clinical trials	Autologous SCT	Brachytherapy	Cellular therapies[1]	Inpatient chemotherapy	Neurosurgery	Phase I/II trials	Orthopaedic surgery	Outpatient chemotherapy	Photon therapy	Proton therapy	Solid tumour surgery[2]	Survivorship clinic
Australia	9														
Austria	5														
Belgium	7														
Canada	18														
Czech Republic	2														
Denmark	4														
Estonia	2														
Finland	5														
France	30														
Germany	60														
Greece	7														
Iceland	1														
Ireland	1														
Italy	40														
Latvia	1														
Lithuania	2														
Netherlands	1														
New Zealand	2														

Norway	4														
Poland	18														
Portugal	4														
Slovakia	3														
Slovenia	1														
Spain	40														
Sweden	6														
Switzerland	9														
UK	21														
US	200														
**Number of responses on availabilities of types of care within a country (n)**															
All centres n		**3**	**15**	**7**	**5**	**3**	**28**	**12**	**3**	**12**	**28**	**15**	**1**	**18**	**16**
Selected centres n		**23**	**11**	**19**	**13**	**21**	**0**	**16**	**21**	**16**	**0**	**12**	**13**	**10**	**11**
Not available (sent abroad) n		**2**	**1**	**2**	**9**	**4**	**0**	**0**	**4**	**0**	**0**	**1**	**12**	**0**	**0**
Not available (also not abroad) n		**0**	**1**	**0**	**1**	**0**	**0**	**0**	**0**	**0**	**0**	**0**	**2**	**0**	**1**

[1]E.g., CAR-T therapy; [2]Not CNS, not orthopaedic; Abbreviations: SCT, stem cell transplantation

Supplemental Table S5: Detailed information about quality criteria by countries, reasons for exclusion marked in italic

Country	Origin of quality criteria	Quality criteria implemented in daily practice?	Name of document or website with quality criteria in original language	Name of document or Website with quality criteria translated to English	Author, publisher, or organization that provides quality criteria (publication year of document)	Type of document or website	Link to website with (document with) quality criteria
**Australia**	The leading region in the state of Victoria produced/ published statewide standards of care across all tumour groups. These are adopted nationally. Additional national network adopting standards of care.	Expert did not know	Victorian paediatric oncology care pathways providing optimal care for children and adolescents – acute leukaemia, central nervous system tumours and solid tumours[5]	-	Paediatric Integrated Cancer Service (2019)	Care pathways	https://www.vics.org.au/pics-about-us
**Belgium**	National local/hospital intern	Never came into effect, currently under revision	Koninklijk besluit houdende vaststelling van de normen waaraan het gespecialiseerd zorgprogramma voor pediatrische hemato-oncologie en het satellietzorgprogramma voor pediatrische hemato-oncologie moeten voldoen om te worden erkend[10]	Royal Decree establishing the standards that the specialized care program for pediatric hemato-oncology and the satellite care program for pediatric hemato-oncology must meet in order to be recognized	Federale overheidsdienst, veiligheid van de voedelketen en leefmilieu<br><br>De Minister van Volksgezondheid, Mevr. L. ONKELINX (2014)	Government document (Royal Decree)	https://etaamb.openjustice.be/nl/koninklijk-besluit-van-02-april-2014_n2014024119.html
**Canada**	Provincial Group (Pediatric Oncology Group of Ontario)	No	Measuring the Quality of a Childhood Cancer Care Delivery System: Assessing Stakeholder Agreement[15]	-	Bradley et al. (2013)	Research article	https://www.valueinhealthjournal.com/article/S1098-3015(13)01701-4/fulltext<br><br>https://www.pogo.ca/
**Czech Republic**	National	Yes	Criteria defining the status of Comprehensive Cancer Centre[6]	-	Ministry of health (2008)	Government document (website format)	https://www.linkos.cz/english-summary/national-cancer-control-programme/czech-cancer-centre-network/criteria-defining-the-status-of-comprehensive-cancer-centre/
**Denmark**	National	Yes	Dansk Børnecancer Register. National årsrapport for perioden	Danish childhood cancer registry, annual report for the period	Dansk Børnecancer Register (2023)	Report	https://www.sundhed.dk/content/cms/87/16287_dbcr-aarsrapport-2022.pdf

			01.01.2022 - 31.12.2022[2]	01.01.2022 - 31.12.2022			
**Estonia**	*Local/hospital intern*	Yes	-	-	-	-	Not publicly available
**Finland**	*Local/hospital intern*	Yes	-	-	-		Not publicly available
**France**	National	Yes	La lutte contre les cancers pédiatriques en France Enjeux, actions et perspectives[16]	The fight against pediatric cancers in France -Challenges, actions and prospects	The French National Cancer Institute (Institut national du cancer, INCa) (2022)	Report *No quality criteria*	https://www.e-cancer.fr/Professionnels-de-sante/L-organisation-de-l-offre-de-soins/Cancerologie-pediatrique
			Critères d'agrément pour la pratique du traitement des cancers des enfants et adolescents de moins de 18 ans[11]	Accreditation criteria for cancer treatment of children and adolescents under 18 years	INCa (2007)	Document with accreditation criteria	https://www.e-cancer.fr/Professionnels-de-sante/L-organisation-de-l-offre-de-soins/Traitements-du-cancer-les-etablissements-autorises/Les-autorisations-de-traitement-du-cancer#toc-les-six-mesures-transversales-de-qualit-
			Proposition d'évolution des critères d'agrément des établissements de santé pour le traitement du cancer[17]	Proposed changes to accreditation criteria for cancer treatment facilities	INCa (2020)	Report/ expert appraisal	
			Décret n°2022-689 du 26 avril 2022 relatif aux conditions d'implantation de l'activité de soins de traitement du cancer[12]	Decree no. 2022-689 of April 26, 2022 on the conditions for setting up cancer treatment facilities	Ministry of Health and Solidarity (Ministére des solidarités et de la santé) (2022)	Government document	https://www.legifrance.gouv.fr/jorf/id/JORFTEXT000045668512
			Décret n°2022-693 du 26 avril 2022 relatif aux conditions techniques de fonctionnement de l'activité de soins de traitement du cancer[13]	Decree no.2022-693 of April 26, 2022 on the technical operating conditions for cancer treatment services	Ministry of Health and Solidarity (Ministére des solidarités et de la santé) (2022)	Government document	https://www.legifrance.gouv.fr/jorf/id/JORFTEXT000045668609
**Germany**	Local/hospital intern, national	Yes	Datenblatt Kinderonkologie (Version D 2.1): Kennzahlenbogen, Datendefizite[18]	Data sheet paediatric oncology (version D 2.1): key data sheet, data deficits	OnkoZert (Status 23 Nov 2022)	Collection forms for certification	https://www.onkozert.de/organ/kinder/
			Richtlinie zur Kinderonkologie, KiOn-RL[19]	Guideline on paediatric oncology, KiOn-RL	The Federal Joint Committee (Gemeinsamer Bundesausschuss, G-BA) (2006, updated 2022)	Government document	https://www.g-ba.de/richtlinien/47/
**Greece**	National	Yes	ΝΟΜΟΣ ΥΠ' ΑΡΙΘΜ. 4461 Μεταρρύθμιση της Διοικητικής Οργάνωσης των υπηρεσιών ψυχικής	LAW NO. 4461 Reform of the Administrative Organization of Mental	The Government of the Hellenic Republic (2017)	Government document,	https://www.taxheaven.gr/law/4461/2017

			υγείας, Κέντρα Εμπειρογνω_μοσύνης σπάνιων και πολύπλοκων νοσημάτων, τροποποίηση συνταξιοδοτικών ρυθμίσεων του ν. 4387/2016 και άλλες διατάξεις.[20]	Health Services, Centres of Expertise for Rare and Complex Diseases, amendment of the pension regulations of Law No. 4387/2016 and other provisions.		*No quality criteria specific for pediatric oncology*	
**Italy**	National	Yes	Check list per la candidatura di un nuovo Centro o per l'aggiornamento delle caratteristiche di un già Centro AIEOP[8]	Checklist for applying for a new centre or updating the characteristics of an existing AIEOP Center	Associazione Italiana Ematologia Oncologia Pediatrica (AIEOP) (AIEOP regulations: 2009, updated 2023)	Appendix of the regulations for members of the national pediatric oncology society	https://www.aieop.org/web/wp-content/uploads/2023/04/ALLEGATO-3-Autocertificazione-Centri.pdf
**Lithuania**	Local/hospital intern, national	Yes	Įsakymas dėl vaikų onkohematologijos antrinio ir tretinio lygio stacionarinių asmens sveikatos priežiūros paslaugų teikimo specialiųjų reikalavimų aprašo patvirtinimo[9]	Order on the approval of the special requirements for the provision of secondary and tertiary level inpatient personal healthcare services in paediatric onco-haematology	Lietuvos respublikos sveikatos apsaugos ministras (2006, updated 2021)	Government document	https://e-seimas.lrs.lt/portal/legalAct/lt/TAD/TAIS.281201/asr
**Netherlands**	National	Yes	Verantwoorde en veilige zorg voor kinderen met kanker in Nederland - Normering Kinderoncologie[3]	Responsible and safe care for children with cancer in the Netherlands -Pediatric Oncology Standards	Stichting Kinderoncologie Nederland (SKION) and Vereniging Ouders, Kinderen en Kanker (VOKK)	Pediatric oncology standards document	-
**Norway**	*Local/hospital intern*	Yes	-	-	-	-	Not publicly available
**Poland**	Local/hospital intern, national based on European/worldwide standards of care	Yes	Onkologia dziecięca w Polsce – stan obecny, osiągnięcia i potrzeby[14]	Paediatric oncology in Poland – current state, achievements and needs	Hematoonkologia.pl	Website about pediatric oncology<br><br>(Standards for Pediatric Oncology and Hematology can be found on website, login necessary)	https://onkologia-dziecieca.pl/chorzy/news/id/3279-onkologia-dziecieca-w-polsce-stan-obecny-osiagniecia-i-potrzeby
**Slovakia**	National	Yes	-	-	-	-	Not publicly available
**Slovenia**	National, Inernational (*SIOP, ESMO, JACIE*)	Yes	-	-	-	-	National not publicly available<br><br>*No quality criteria available from SIOP and ESMO, JACIE sub-specialty specific*
**Sweden**	National	Yes	-	-	-	-	Expert did not know

**Switzerland**	National	Yes	Schweizerische Pädiatrische Onkologie Gruppe (SPOG) Statuten[7]	Swiss paediatric oncology group (SPOG) Statutes	Swiss paediatric oncology group (1991, updated 2022)	Regulations for members of the national paediatric oncology society	https://spog.ch/ueber-uns/ueber-spog/
			Bereichsspezifische Qualitätskriterien, die im Bewerbungsverfahrensverfahren für Hochspezialisierte Medizin (HSM) Leistungsaufträge geprüft werden	Quality criteria examined in the application process for Highly Specialized Medicine service mandates	Conference of Cantonal Health Directors (Konferenz der kantonalen Gesundheitsdirektorinnen und -direktoren, GDK) and HSM expert body	Requirements catalog for the application for HSM services mandate	Not publicly available, will be available here: https://www.gdk-cds.ch/de/hochspezialisierte-medizin/bereiche/hochspezialisierte-paediatrische-onkologie
**United Kingdom**	National, NICE	Yes	Cancer services for children and young people[21]	-	National Institute for Health and Care Excellence (NICE) (2014)	Quality standard document	https://www.nice.org.uk/guidance/qs55
**United States**	Local/hospital intern, national	Yes	Optimal Resources for Cancer Care – 2020 Standards[4] (Category: Pediatric Cancer Program (PCP))	-	American college of surgeons (2020, updated 2023)	Document with standards that serve as qualification criteria for Commission on Cancer (CoC) accreditation	https://www.facs.org/for-medical-professionals/news-publications/news-and-articles/cancer-programs-news/040722/coc/

Supplemental Table S6: Responses about certifications of paediatric oncology centres, including reasons for exclusion from the final results

Country	Possibility to certify paediatric oncology centres	Is a certification mandatory?	Name of the organization/program where one can apply for the certification/accreditation	How often does the re-certification/ accreditation take place?	Reason for exclusion from results
Austria	Yes	No	Among others: International Organization for Standardization (ISO), The Joint Accreditation Committee ISCT-Europe & EBMT (JACIE)	Regular intervals, ISO (yearly), but different types of audits	ISO: General certification<br>JACIE: Subspecialty-specific certification for bone marrow transplantation<br>Both: Not specific to paediatric oncology centres
Czech Republic	Yes	Expert did not know	JACIE<br>Spojená akreditační komise (SAK) - Joint accreditation committee of the Czech Republic	Every 3 years	JACIE: Subspecialty-specific certification<br>SAK: General certification for healthcare institutions<br>Both: Not specific paediatric oncology centres
Finland	Yes	No	Social and Health Quality Service (SHQS), Finnish Medicines Agency (FIMEA)	Every 3 years for FIMEA. SHQS internal audit annually	FIMEA: Subspecialty-specific certification<br>SHQS: Not specific to paediatric oncology centres
France	Yes	Yes	Not actual certification but authorization by regional health agencies in agreement with Société Française de Lutte contre les Cancers et les Leucémies de l'Enfant et de l'Adolescent (SFCE) et The French National Cancer Institute (Institut National du Cancer, INCa): national cancer Institut*	I think every 4-5 years	-
Germany	Yes	No	OnkoZert	Yearly	-
Greece	Yes	No	Ministry of Health, Certification of Centers of expertise in accordance with European Reference networks accreditation	Every 5 years	European Reference networks accreditation: Is a prerequisite for joining the ERN PaedCan
Ireland	Yes	No	Organisation of European Cancer Institutes (OECI)	Not sure	Not specific to paediatric oncology centres
Italy	Yes	Yes	Associazione Italiana Ematologia Oncologia Pediatrica (AIEOP)**	Expert did not know	-
Poland	Yes	Yes	Ministry of Health decides on the need for the new pediatric oncology center, then the local authorities take care of finding trained staff, location and hospital builds procedures.	Depends on the centre, if the hospital as a whole needs accreditation then the whole process starts. I should say every 3- 5 years.	-
Portugal	Yes	No	Directorate General of Health	3-5 years	General certification
Switzerland	Yes	No	OnkoZert	Yearly	-
United Kingdom	Yes	Yes	Only for NHS (National Health service) hospitals - accredited centres determined by the government	3-5 yearly	General certification
United States	Yes	Expert did not know	Commission on cancer	-	-

*Translated to English
**Self-certification checklist for applying for a new centre or updating the characteristics of an existing AIEOP centre (see Table 3)

Supplemental Table S7: Overview of epidemiological data and the availability of quality criteria and certification possibilities from the survey, listed by number of new paediatric cancer patients per year

Country	Population aged 0-19 (thousands)*	Number of new paediatric cancer cases/year	Number of treatment centres (n)	Quality criteria	Certification
US	83 524	17000	200	Yes	Yes
France	15 120	2500	30	Yes	Yes
Italy	10 342	2300	40	Yes	Yes
Germany	15 433	2000	60	Yes	Yes
UK	15 634	1800	21	Yes	No
Australia	6 264	1500	9	Yes	No
Spain	9 166	1400-1500	40	No	No
Poland	7 695	1200	18	Yes	Yes
Canada	8 054	1000	18	Yes	No
Netherlands	3 735	600	1	Yes	No
Belgium	2 585	450	7	Yes	No
Czech Republic	2 173	400	2	Yes	No
Portugal	1 908	400	4	No	No
Switzerland	1 730	400	9	Yes	Yes
Greece	2 017	350	7	No	Yes
Sweden	2 448	350	6	No	No
Austria	1 717	300	5	No	No
Finland	1 155	210	5	No	No
Norway	1 234	200	4	No	No
Denmark	1 290	180-200	4	Yes	No

Ireland	1 319	180-200	1	No	No
Slovakia	1 129	180	3	No	No
New Zealand	1 288	160	2	No	No
Slovenia**	418	70	1	No	No
Lithuania	1 002	60-70	2	Yes	No
Latvia	384	50-70	1	No	No
Estonia	285	50	2	No	No
Iceland	91	14	1	No	No

Quality criteria and certification “yes”

Quality criteria and certification “no”

* Estimate 2021 from: https://population.un.org/wpp/Download/Standard/Population/[22]

References

1. Organization for Economic Co-operation and Development (OECD). Health at a Glance 2021: OECD Indicators. Paris: OECD Publishing; 2021. Available from: https://www.oecd-ilibrary.org/social-issues-migration-health/health-at-a-glance-2021_ae3016b9-en [accessed 2023 March 15].
2. Dansk Børnecancer Register. Dansk Børnecancer Register. National årsrapport for perioden 01.01.2022 - 31.12.2022. 2023. Available from: https://www.sundhed.dk/content/cms/87/16287_dbcr-aarsrapport-2022.pdf [accessed 2023 Oct 05].
3. Stichting Kinderoncologie Nederland (SKION), Vereniging Ouders, Kinderen en Kanker (VOKK). Verantwoorde en veilige zorg voor kinderen met kanker in Nederland - Normering Kinderoncologie. 2012.
4. American college of surgeons. Optimal Resources for Cancer Care - 2020 Standards. Chicago; 2023. Available from: https://www.facs.org/quality-programs/cancer-programs/commission-on-cancer/standards-and-resources/2020/ [accessed 2023 Oct 05].
5. Paediatric Integrated Cancer Service. Victorian paediatric oncology care pathway: Providing optimal care for children and adolescents — Acute leukaemia, central nervous system tumours and solid tumours. Melbourne, Australia: Paediatric Integrated Cancer Service; 2019. Available from: https://dokumen.tips/documents/victorian-paediatric-oncology-care-pathways-victorian-paediatric-oncology-care-pathways.html?page=1 [accessed 2023 Oct 05].
6. Czech Cancer Centre Network. Criteria defining the status of Comprehensive Cancer Centre: Czech Cancer Centre Network; 2008. Available from: https://www.linkos.cz/english-summary/national-cancer-control-programme/czech-cancer-centre-network/criteria-defining-the-status-of-comprehensive-cancer-centre/ [accessed 2023 Aug 15].
7. Schweizerische Pädiatrische Onkologie Gruppe (SPOG). SPOG Statuten. 2022. Available from: https://spog.ch/wp-content/uploads/2023/07/SPOG_Statuten_d_20221124.pdf [accessed 2023 Sep 04].
8. Associazione Italiana Ematologia Oncologia Pediatrica (AIEOP). Check list per la candidatura di un nuovo Centro o per l'aggiornamento delle caratteristiche di un già Centro AIEOP Available from: https://www.aieop.org/web/wp-content/uploads/2023/04/Regolamento-AIEOP.pdf [accessed 2023 Oct 05].
9. Lietuvos respublikos sveikatos apsaugos ministras. Įsakymas dėl vaikų onkohematologijos antrinio ir tretinio lygio stacionarinių asmens sveikatos priežiūros paslaugų teikimo specialiųjų reikalavimų aprašo patvirtinimo. Vilnius: Lietuvos Respublikos sveikatos apsaugos ministerija; 2021. Available from: https://e-seimas.lrs.lt/portal/legalAct/lt/TAD/TAIS.281201/asr [accessed 2023 Oct 05].
10. Federale overheidsdienst volksgezondheid, veiligheid van de voedselketen en leefmilieu. Koninklijk besluit houdende vaststelling van de normen waaraan het gespecialiseerd zorgprogramma voor pediatrische hemato-oncologie en het satellietzorgprogramma voor pediatrische hemato-oncologie moeten voldoen om te worden erkend 2014. Available from: https://etaamb.openjustice.be/nl/koninklijk-besluit-van-02-april-2014_n2014024119.html [accessed 2023 Aug 15].
11. Institut national du cancer. Critères d'agrément pour la pratique du traitement des cancers des enfants et adolescents de moins de 18 ans. Available from: https://www.proinfoscancer.org/sites/default/files/2012_criteres_agrement_oncopediatrie_inca_pro.pdf [accessed 2023 Oct 05].
12. Ministére des solidarités et de la santé. Décret no 2022-689 du 26 avril 2022 relatif aux conditions d'implantation de l'activité de soins de traitement du cancer. 2022. Available from: https://www.legifrance.gouv.fr/jorf/id/JORFTEXT000045668512 [accessed 2023 Oct 05].
13. Ministére des solidarités et de la santé. Décret no 2022-693 du 26 avril 2022 relatif aux conditions techniques de fonctionnement de l'activité de soins de traitement du cancer. 2022. Available from: https://www.legifrance.gouv.fr/loda/id/JORFTEXT000045668609 [accessed 2023 Oct 05].
14. Kowalczyk J, Perek D, Balwierz W, Matysiak M, Szczepański T, Młynarski W, et al. Standardy oddziału onkologii i ematologii dziecięcej - Standards of pediatric oncology/hematology ward. Przegląd Pediatryczny. 2018;47(2):57-61
15. Bradley NM, Robinson PD, Greenberg ML, Barr RD, Klassen AF, Chan YL, et al. Measuring the quality of a childhood cancer care delivery system: assessing stakeholder agreement. Value Health. 2013;16(4):639-46
16. Institut national du cancer. La lutte contre les cancers pédiatriques en France. Enjeux, actions et perspectives. 2022.

17. Institut national du cancer. Propositions d'évolution des critères d'agrément des établissements de santé pour le traitement du cancer. 2020. Available from: https://www.e-cancer.fr/Expertises-et-publications/Catalogue-des-publications/Proposition-d-evolution-des-criteres-d-agrement-des-etablissements-de-sante-pour-le-traitement-du-cancer [accessed 2023 Oct 05].
18. OnkoZert. Datenblatt Kinderonkologie. Available from: https://www.onkozert.de/organ/kinder/ [accessed 2023 Sep 04].
19. Gemeinsamer Bundesausschuss. Richtlinie zur Kinderonkologie. Berlin; 2022. Available from: https://www.g-ba.de/richtlinien/47/ [accessed 2023 June 25].
20. NOMO NO 4461 Reform of the Administrative Organization of Mental Health Services, Centres of Expertise for Rare and Complex Diseases, amendment of the pension regulations of Law No. 4387/2016 and other provisions. E Press of the Government of the Hellenic Republic. Athens: The Government of the Hellenic Republic 2017. p. 417-94.
21. National Institute for Health Care Excellence (NICE). Cancer Services for Children and Young People. London; 2014. Report No.: 978-1-4731-0454-9 Available from: https://www.nice.org.uk/Guidance/QS55 [accessed 2023 June 25].
22. United Nations, Department of Economic and Social Affairs, Population Division. World Population Prospects 2022, Online Edition. 2022. Available from: https://population.un.org/wpp/Download/Standard/Population/ [accessed 2023 Dec 14].
